# Early Childhood Movement- and Arts-Based Experiences and Executive Functions: A Systematic Review and Meta-Analysis

**DOI:** 10.3390/children13091134

**Published:** 2026-08-24

**Authors:** Gustavo Vega-Fernandez, Juan José Valenzuela-Fuenzalida, Yasna Jeria-Pizarro, Gustavo Oyanedel-Amaro, Mathias Orellana-Donoso, Gloria Cifuentes-Suazo, Juan J. Cabezas-Salgado, Christopher Blackwood-Espinoza, José E. León-Rojas, Juan Sanchis-Gimeno, Alejandro Bruna-Mejias

**Affiliations:** 1 Laboratory of Epidemiology and Morphological Sciences, Instituto de Biología, Pontificia Universidad Católica de Valparaíso, Valparaíso 2373223, Chile; gustavo.vega@pucv.cl; 2 Departamento de Morfología, Facultad de Medicina, Universidad Andrés Bello, Viña del Mar 2520000, Chile; juan.valenzuela.f@unab.cl (J.J.V.-F.); alejandro.bruna@unab.cl (A.B.-M.); 3 Departamento de Ciencias Química y Biológicas, Facultad de Ciencias de la Salud, Universidad Bernardo O’Higgins, Santiago 8370993, Chile; 4 Facultad de Ciencias de la Salud, Universidad Autónoma de Chile, Santiago 8910060, Chile; yasna.jeria@cloud.uautonoma.cl; 5 Faculty of Health and Social Sciences, University of the Americas, Santiago 8370040, Chile; g.oyanedelamaro@gmail.com; 6 Escuela de Medicina, Universidad Finis Terrae, Santiago 7501015, Chile; morellanad@uft.edu; 7 Facultad de Medicina y Ciencia, Universidad San Sebastián, Lota 2465, Santiago 7510157, Chile; 8 Carrera de Odontología, Facultad de Medicina, Universidad Católica de la Santísima Concepción, Av. Alonso de Ribera 2850, Concepción 4090541, Chile; gloria.cifuentes@ucsc.cl; 9 Facultad de Medicina, Universidad Católica del Maule, Talca 3460000, Chile; jcabezas@ucm.cl; 10 Escuela de Ciencias de la Salud, Universidad Viña del Mar, Viña del Mar 2520000, Chile; christopher.blackwood@docente.uvm.cl; 11 Cerebro, Emoción y Conducta (CEC) Research Group, Escuela de Medicina, Universidad de las Américas (UDLA), Quito 170521, Ecuador; 12 GIAVAL Research Group, Faculty of Medicine, University of Valencia, 46001 Valencia, Spain; juan.sanchis@uv.es; 13 Departamento de Ciencias y Geografía, Facultad de Ciencias Naturales y Exactas, Universidad de Playa Ancha, Valparaíso 2360072, Chile

**Keywords:** early childhood, preschool, executive function, physical activity, motor competence, music, arts, dance, meta-analysis

## Abstract

**Highlights:**

**What are the main findings?**

- Across 15 Study IDs and 29 model-ready effects, average benefits were observed for inhibition, working memory and cognitive flexibility, but all prediction intervals crossed the null, and GRADE certainty was low.
- The quantitative core comprised 12 primarily structured programmes and three guided-play/structured-hybrid programmes; no free or unstructured-play intervention entered the meta-analysis.

**What are the implication of the main findings?**

- Benefits should not be generalised to movement, play or arts participation per se; pedagogical structure and deliberately embedded executive demands may influence outcomes.
- Future trials should report task progression, complexity, teacher guidance, child agency, implementation fidelity and cluster-adjusted analyses sufficiently to identify active programme components.

**Abstract:**

Background/Objectives: Movement- and arts-based experiences are frequently promoted as developmentally appropriate contexts for supporting cognition in early childhood, but effects may differ across executive-function domains, pedagogical formats and study contexts. Methods: This PRISMA 2020 systematic review and meta-analysis was registered in PROSPERO (CRD420261363924). PubMed/MEDLINE, Web of Science, Scopus, ERIC, PSICODOC, CINAHL and SPORTDiscus were searched from inception through April 2026. One immediate-post effect per Study ID and core domain was retained. Random-effects models used restricted maximum likelihood with Hartung–Knapp adjustment. Study-level risk of bias was assessed with RoB 2 or ROBINS-I, missing-evidence risk with ROB-ME and certainty with GRADE. Intervention content, pedagogical format and embedded cognitive demands were also coded descriptively. Results: The review included 130 reports representing 125 unique studies. Fifteen Study IDs contributed 29 core effects: 10 for inhibition (n = 675), 10 for working memory (n = 697) and nine for cognitive flexibility (n = 642). Twelve core programmes were primarily structured, and three used guided-play/structured-hybrid formats; no free/unstructured-play intervention entered the meta-analysis. Mean effects favoured the intervention for inhibition (Hedges’ g = 0.52, 95% CI 0.15 to 0.90), working memory (g = 0.42, 95% CI 0.09 to 0.75) and cognitive flexibility (g = 0.60, 95% CI 0.22 to 0.98). Heterogeneity was substantial, and all 95% prediction intervals crossed the null. Estimates remained favourable after excluding Serious risk-of-bias judgments. Age-exclusion and ICC-sensitivity analyses retained positive effects; a worst-case exclusion of unadjusted class- or cluster-assigned studies reduced precision for inhibition and working memory. SMD-specific modified-precision regressions did not indicate small-study associations for inhibition (*p* = 0.529) or working memory (*p* = 0.619); cognitive flexibility was not formally tested because k = 9. ROB-ME indicated some concerns, and GRADE certainty was low for all three domains. Conclusions: Favourable average effects should not be attributed to movement or arts participation per se. Pedagogical structure and embedded cognitive demands may influence outcomes, but substantial inconsistency, unit-of-analysis uncertainty and low certainty preclude identification of a superior model. Funding: There was no external funding.

## 1. Introduction

Early childhood is a developmental period in which the cognitive systems that support goal-directed behaviour become increasingly differentiated, observable and responsive to everyday learning contexts. During the preschool years, children make marked gains in the capacity to inhibit prepotent responses, hold and manipulate information, and flexibly shift between rules or task demands [1,2,3,4]. These executive functions are central to school readiness because they support attention regulation, behavioural control, problem solving and adaptive participation in structured educational settings [3,5]. For this reason, identifying developmentally appropriate experiences that may support executive-function growth before or during the transition to school has become a priority for developmental, educational and public-health research [6,7].

Executive functions are commonly conceptualised as a set of interrelated but distinguishable cognitive processes. The most frequently examined core components are inhibitory control, working memory and cognitive flexibility [2,3]. Inhibitory control refers to the ability to suppress dominant or distracting responses; working memory involves maintaining and manipulating information over short intervals; and cognitive flexibility supports shifting between rules, perspectives or response sets [2,3]. Broader self-regulation overlaps with these processes but also includes motivational, emotional and behavioural dimensions that are not identical to task-based executive-function performance [4,5]. This distinction is methodologically important: interventions may influence specific executive-function tasks without producing equivalent changes in broader behavioural self-regulation, and pooled analyses should not treat these constructs as interchangeable.

Movement-based experiences are a plausible context for supporting executive-function development because many structured physical activities require more than energy expenditure. Active play, sport, motor-skill practice, gymnastics, exergaming and cognitively enriched physical activity often require children to monitor rules, inhibit impulsive actions, remember sequences, coordinate actions with peers, adapt to feedback and update motor plans in real time [8,9,10,11]. These task demands create a theoretically meaningful bridge between motor engagement and executive control. The strongest rationale is therefore not that physical activity is uniformly beneficial for cognition but that structured and cognitively demanding movement may provide repeated opportunities to practise executive-function-relevant processes in embodied, socially meaningful contexts [6,7,8].

Arts-based experiences may engage executive functions through partly overlapping but distinct pathways. Music training, rhythm activities, dance, drama and music-movement interventions require sequencing, auditory-motor coupling, imitation, turn-taking, sustained attention, error monitoring and flexible adaptation to changing cues [12,13,14,15,16]. These demands are particularly relevant during early childhood, when children are learning to coordinate action, attention, memory and social rules within structured group activities. Nevertheless, arts participation is not a single exposure. Music lessons, dance, creative drama and mixed movement-arts programmes differ in cognitive load, instructional structure, duration, intensity and social context. Accordingly, broad claims that arts improve cognition are not sufficiently precise. A domain-specific synthesis is needed to determine whether particular executive-function outcomes appear more responsive to these experiences than others [7,17].

For interpretation, intervention content and pedagogy were treated as related but non-equivalent dimensions. Content was classified as movement/motor, arts, or mixed movement-arts. Pedagogical format was classified as structured, guided play or free/unstructured play. Structured interventions were adult-designed programmes with predefined objectives, scheduled sessions, explicit instructions and a planned activity sequence or progression. Guided play retained meaningful child choice or agency within an adult-defined goal, environment or set of task constraints, with scaffolding when required. Free or unstructured play was predominantly child-initiated and child-directed, without a standardised instructional sequence, externally prescribed executive-function goal or planned progression.

The terms cognitively engaging and cognitively enriched were used only when an activity deliberately embedded executive demands beyond physical or artistic execution alone. Qualifying features included maintaining or updating rules and sequences, inhibiting prepotent responses, switching actions or strategies, responding to changing cues, solving movement-related problems, coordinating dual tasks and making decisions under task-imposed constraints. These dimensions are orthogonal: a motor or arts programme may be structured or guided, and only some programmes explicitly embed executive demands. This framework therefore avoids treating an intervention label as a mechanism and provides a pedagogical basis for interpreting between-study heterogeneity.

Existing reviews have typically examined physical activity, motor competence, music training and cognitive training separately, often across broad age ranges that combine preschool and school-aged children [6,7,8,12,17]. This creates two important limitations. First, it obscures whether evidence from older children can be applied to the early-childhood period, when executive functions are still emerging and measurement is especially sensitive to task demands. Second, it limits comparison across exposure families that may share cognitively demanding features, such as rule use, sequencing, inhibition and flexible adaptation, despite differing in content and delivery. To date, the literature has lacked an early-childhood-focused synthesis that integrates movement-based, arts-based and mixed movement-arts experiences while preserving domain-specific executive-function outcomes.

Therefore, this systematic review and meta-analysis aimed to synthesise evidence on early childhood movement- and arts-based experiences in relation to executive-function outcomes. Quantitative synthesis was prespecified separately for inhibitory control, working memory and cognitive flexibility. Broader behavioural self-regulation, hot executive function/delay gratification, overall task-based composites and spatial working-memory tasks were retained as secondary outcomes and were pooled only when construct, estimand and independence criteria were satisfied.

## 2. Materials and Methods

### 2.1. Protocol and Reporting

The review was prospectively registered in PROSPERO as version 1.0 on 13 April 2026 (CRD420261363924; https://www.crd.york.ac.uk/PROSPERO/view/CRD420261363924, accessed on 9 August 2026) and was reported according to PRISMA 2020 [18]. The original standalone protocol is publicly accessible through the PROSPERO record and has been preserved without retrospective modification. Version 1.0 prespecified the central review question, the birth-to-72-month exposure window, the E1/E2/E3 exposure framework, broad executive-function and visuospatial outcomes and random-effects synthesis when evidence was sufficiently comparable. Version 2.0 was published on 14 July 2026 after completion of the review and documented the final title, seven-database set, operational eligibility wording, outcome hierarchy, data-extraction process and synthesis plan; these details are therefore not all presented as prospective prespecification. Minor amendments during peer-review revision made the protocol public, documented the definitive 15-Study-ID/29-effect dataset and subsequent sensitivity analyses, and corrected the review end date to 10 July 2026. The original-versus-amended elements and their timing are summarised in Supplementary Table S10.

### 2.2. Eligibility Criteria

Eligible studies included children exposed to movement-, physical activity-, motor-, arts-, music-, dance-, drama- or mixed movement-arts experiences initiated, delivered or measured from birth through 72 completed months. Outcomes could be assessed later when the eligible exposure occurred within this early-childhood window; age at exposure, rather than age at outcome assessment alone, governed eligibility. The broad window was chosen because executive functions undergo rapid developmental change throughout early childhood and because the review sought to capture early experiences with both concurrent and later cognitive outcomes. Recognising that exposure timing and measurement reliability may differ across this interval, exact study-level exposure ages were extracted and examined in sensitivity analyses. Core quantitative outcomes were inhibitory control, working memory and cognitive flexibility. Broader behavioural self-regulation, hot executive function/delay gratification, overall task-based composites and spatial working-memory tasks were retained as secondary outcomes and synthesised only when their constructs, estimands and sampling units were compatible. Reviews, protocols, commentaries, conference abstracts, clinical-only samples without separable typically developing data, and studies without an eligible exposure-outcome pair were excluded.

### 2.3. Information Sources and Search

The final search covered PubMed/MEDLINE, Web of Science, Scopus, ERIC, PSICODOC, CINAHL and SPORTDiscus from database inception through April 2026, without date or language restrictions. Search terms combined concepts for early childhood or preschool age, physical activity or movement, motor competence, arts or music or dance or drama, and executive-function or cognitive outcomes. The originally registered strategy and database-adapted reproducibility strings for all seven final databases are reproduced in Supplementary File S1. The historical archive did not preserve complete date-stamped platform exports, exact source-specific execution dates or per-database yields for all sources; the final database-adapted strings are therefore labelled as reproducibility reconstructions rather than verbatim historical execution logs. Google Scholar, reference-list checking and forward citation chasing were used only for contextual checking and were not retained as formal PRISMA identification sources.

### 2.4. Study Selection and Study-Level Consolidation

Records were deduplicated in Zotero and managed for screening in Rayyan. Title/abstract screening and full-text eligibility assessment were conducted independently by two reviewers using predefined criteria. Disagreements were resolved by discussion and, when needed, third-reviewer methodological adjudication. Non-English reports were assessed with translation support when required. Thirteen retrieval-stage reports could not be obtained or did not contain sufficient accessible full-text material for an eligibility decision; because the archived project log did not preserve a reliable report-level split between these two circumstances, they are reported as a combined category rather than retrospectively reclassified. Reports were subsequently consolidated at the study level to avoid double-counting companion reports, repeated samples or multiple reports from the same project. For quantitative synthesis, each Study ID contributed at most one selected effect per outcome domain and timepoint.

### 2.5. Data Extraction, Conceptual Classification and Effect-Size Computation

Data were extracted independently by two reviewers using structured forms, with discrepancies resolved by consensus and, when required, third-reviewer adjudication. Extracted variables included report and Study ID, exact age at exposure, design, allocation and analysis unit, clustering and reported adjustment, exposure family, comparator, outcome domain and instrument, timepoint, numerical data, effect estimate, variance and information required for risk-of-bias assessment. The complete reviewer-facing extraction register, including study-level age/design information and the selected-effect rationale, is provided in Supplementary Table S11.

To address intervention heterogeneity, the 15 core programmes were additionally coded from the primary reports along three descriptive dimensions: content family (movement/motor, arts, or mixed), pedagogical format (structured, guided play, free/unstructured play, or hybrid), and cognitive-engagement status. Structured, guided-play and free-play formats followed the operational definitions introduced above. Cognitive engagement was coded as explicit when the programme deliberately embedded rule maintenance or updating, response inhibition, switching, sequencing, changing-cue response, problem solving, dual-task coordination or decision making; it was coded as implicit/insufficiently operationalised when such demands were plausible but not described clearly enough to verify enrichment.

Adult guidance, child agency, task progression or complexity, feedback/scaffolding and implementation-fidelity reporting were also recorded. Information not stated in the source was coded as not reported rather than inferred from the intervention label. Because the categories were sparse, partly post hoc and not balanced across domains, this coding was used for descriptive interpretation and not for an underpowered moderator meta-analysis. Study-level decisions are reported in Supplementary Table S13.

For the core intervention synthesis, one immediate-post effect was retained per Study ID and executive-function domain. The hierarchy prioritised direct construct correspondence; a design-appropriate, source-preferred adjusted estimand when available; immediate-post timing; independence within Study ID, domain, timepoint and contrast; and the validated or clearly described task most representative of the domain. Deterministic composites were not counted in addition to their component outcomes; lower-is-better errors, interference scores or response times were sign-reversed; and alternative eligible tasks were reserved for outcome-selection sensitivity analyses. Effect magnitude, confidence-interval width and statistical significance were not selection criteria. For example, inhibition was measured using Stop Signal accuracy, Go/No-Go response time, Day/Night Stroop scores and related task-error measures; because these instruments use different scales, eligible outcomes were converted to Hedges’ g before synthesis. Positive g values consistently favour the intervention after directional harmonisation. Source-preferred adjusted mean differences, odds ratios, hurdle-model estimates and other non-convertible outcomes retained their source-specific interpretation and were synthesised narratively rather than forced into the SMD model.

### 2.6. Risk of Bias and Certainty of Evidence

Randomised and cluster-randomised intervention studies were assessed using RoB 2, and nonrandomised or quasi-experimental intervention studies were assessed using ROBINS-I [19,20]. Certainty of evidence was evaluated by outcome using GRADE [21] across risk of bias, inconsistency, indirectness, imprecision and publication bias. Longitudinal observational exposure evidence was appraised using ROBINS-E [22]. Risk of bias due to missing evidence was assessed separately for inhibition, working memory and cognitive flexibility using ROB-ME [23]. Risk-of-bias sensitivity analyses excluded studies with an overall Serious judgment. Study-level risk-of-bias assessments were completed within the review workflow; ROB-ME and GRADE were finalised through structured review-team evidence matrices and methodologist adjudication and are not represented as duplicate independent assessments. Allocation-unit and cluster-adjustment limitations identified in the study-level audit were incorporated into the interpretation of risk of bias, imprecision and certainty.

### 2.7. Statistical Analysis

Domain-specific random-effects meta-analyses used restricted maximum likelihood with Hartung–Knapp adjustment [24]. The frozen model-ready dataset contained 29 effects from 15 unique Study IDs. Inhibition, working memory and cognitive flexibility were analysed separately because several projects contributed the same participant sample to more than one domain; no omnibus model treated all 29 rows as mutually independent. Heterogeneity was described using Cochran’s Q, tau-squared, I-squared and 95% prediction intervals. Robustness was examined by excluding Serious risk-of-bias judgments and through leave-one-study-out analyses. Because standardized mean differences and their usual standard errors are mathematically correlated, conventional SMD-versus-standard-error funnel regression was not used. For domains with at least 10 independent effects, small-study associations were examined using the sample-size precision term sqrt(1/nI + 1/nC) in a REML meta-regression with Hartung–Knapp inference [25]. Formal asymmetry testing was withheld when k < 10.

### 2.8. Post-Peer-Review Age and Unit-of-Analysis Sensitivity Analyses

During peer-review revision, primary reports for the 15 core Study IDs were audited for age at exposure, design, allocation unit, analysis unit and cluster adjustment. Exposure ages were classified as 0–23, 24–47, 48–72 months or mixed. Because only two studies included children younger than 48 months and none began before 36 months, developmental heterogeneity was evaluated through exclusion sensitivity rather than an underpowered subgroup meta-analysis or meta-regression. For class- or cluster-assigned studies whose frozen core-effect variance could not be verified as cluster-adjusted, sensitivity analyses inflated the variance using DE = 1 + (m − 1) × ICC under ICC assumptions of 0.01, 0.05 and 0.10. Observed classroom ICCs reported by ST026 were used for such studies. An intentionally conservative scenario excluded all eight class- or cluster-assigned studies lacking verifiable adjusted precision. These analyses assessed robustness and did not replace the primary models with a single arbitrary ICC assumption.

### 2.9. Synthesis Without Meta-Analysis

Evidence not included in a meta-analysis was synthesised using a structured approach aligned with the Synthesis Without Meta-analysis (SWiM) reporting guideline [26]. The corpus was grouped into eight design-specific cells: the core chronic intervention meta-analysis; other chronic interventions; acute independent or quasi-experimental studies; acute paired or crossover studies; longitudinal observational studies; cross-sectional 24 h movement or guideline studies; cross-sectional motor-competence or fitness studies; and cross-sectional activity, sport, play, arts or contextual studies. The synthesis unit was a design-specific unit nested within a Study ID, allowing mixed-design projects to contribute to more than one cell without double-counting studies or reports. No *p*-value vote counting, combination of *p* values or cross-design pooling was used. Direct adjusted findings were prioritised over indirect, contextual, subgroup or model-derived results. Broader behavioural self-regulation, hot executive function/delay gratification, overall task-based executive-function composites and spatial working-memory tasks were retained as secondary outcomes. They were not pooled unless at least two independent studies used conceptually comparable constructs and commensurate estimands. Composite scores derived from core tasks already represented in the domain-specific models were not counted as independent evidence.

## 3. Results

### 3.1. Study Selection

**Figure 1:**
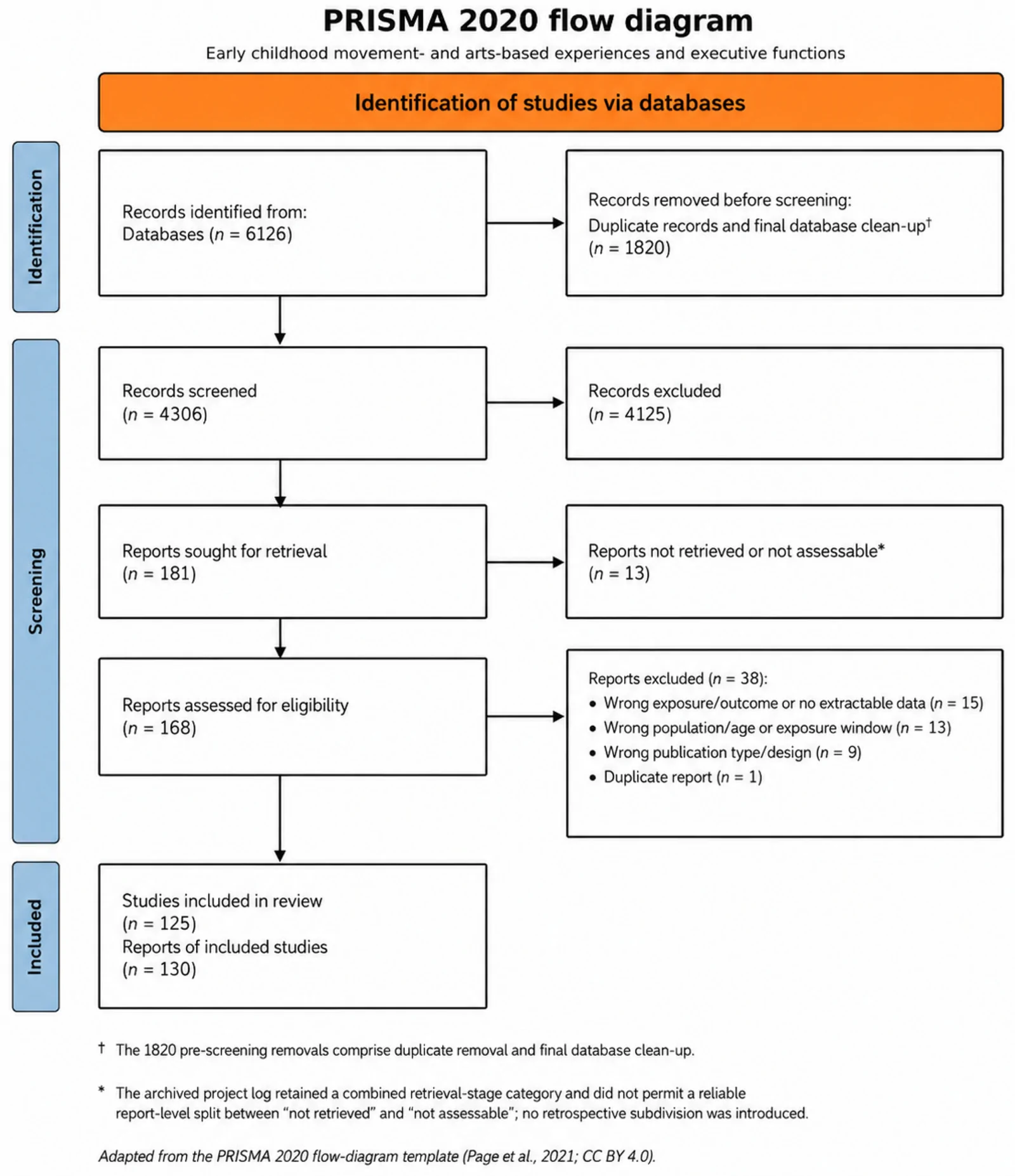
PRISMA 2020 flow diagram [18]. The review included 130 reports representing 125 unique studies; 15 unique Study IDs contributed 29 core executive-function effects to the intervention meta-analysis. The 13 retrieval-stage losses are reported as the combined archived category “not retrieved or not assessable”.

Database searching identified 6126 records. After duplicate removal and final clean-up, 4306 records entered title/abstract screening. Of these, 4125 were excluded and 181 reports were sought for retrieval. Thirteen reports were not retrieved or were not assessable, leaving 168 reports for full-text eligibility assessment. Thirty-eight reports were excluded after full-text assessment; report-level reasons are provided in Table S2. The remaining 130 reports represented 125 unique studies. Fifteen unique Study IDs contributed 29 core executive-function effects: 10 for inhibition, 10 for working memory and nine for cognitive flexibility. Broader self-regulation/hot-executive-function and spatial working-memory results were retained separately and were not added to the core effect count. Figure 1 shows the study-selection process, and Table 1 summarises the report-, study- and effect-level analytic counts.

### 3.2. Characteristics of the Primary Intervention Evidence

The final intervention-evidence audit encompassed 21 candidate Study IDs [11,13,15,27,28,29,30,31,32,33,34,35,36,37,38,39,40,41,42,43,44], of which 15 contributed to the core models. The core set comprised six individually randomised studies, three cluster-randomised studies and six nonrandomised or quasi-experimental studies. By domain, inhibition included five individually randomised studies, two cluster-randomised studies and three nonrandomised/quasi-experimental studies; working memory included four, one and five; and cognitive flexibility included three, two and four, respectively (Table 2). Thirteen core studies were confined to older-preschool samples, principally 48–72 months. Only ST076 and ST096 included children younger than 48 months, and no core exposure began before 36 months. Study-level age, design, allocation-unit and clustering information is reported in Supplementary Table S11. Structured physical-activity and motor programmes included gymnastics, coordination-based training, structured physical activity, mini-trampoline training, group-play moderate-to-vigorous physical activity, exergaming and cognitively engaging movement programmes. Music-, dance-, music-movement- and gesture-based programmes were also represented. Several Study IDs contributed to more than one executive-function domain; these cross-domain rows were treated as dependent at the project level.

Pedagogical profile: All 15 core interventions were adult-designed. Twelve were primarily structured programmes, and three were coded as guided-play/structured hybrids (ST057, ST082 and ST096); no free or unstructured-play intervention contributed to the quantitative core. Free play occurred as a comparator or in non-core acute/observational evidence rather than as a pooled intervention. Explicit rule, sequence, memory, switching, cue-response or decision demands were described in a majority of programmes, but several reports described the activity content and dose without sufficient detail to verify cognitive enrichment. Reporting of task progression, teacher scaffolding, child agency and implementation fidelity was uneven (Supplementary Table S13). The sparse and overlapping categories did not support a defensible pedagogical subgroup meta-analysis.

### 3.3. Risk of Bias

Design-appropriate RoB 2 or ROBINS-I judgments were available for all 15 Study IDs in the quantitative core. Seven studies were rated as having Some concerns, five as Moderate risk and three as Serious risk. Studies with Serious judgments were retained in the primary models for transparency and excluded in prespecified risk-of-bias sensitivity analyses.

### 3.4. Primary Chronic Intervention Meta-Analysis

The final quantitative core comprised 29 domain-specific effects from 15 unique Study IDs. Inhibition, working memory and cognitive flexibility were analysed separately. Summary estimates, heterogeneity, prediction intervals, risk-of-bias sensitivity analyses, ROB-ME judgments and GRADE certainty are presented in Table 3.

**Figure 2:**
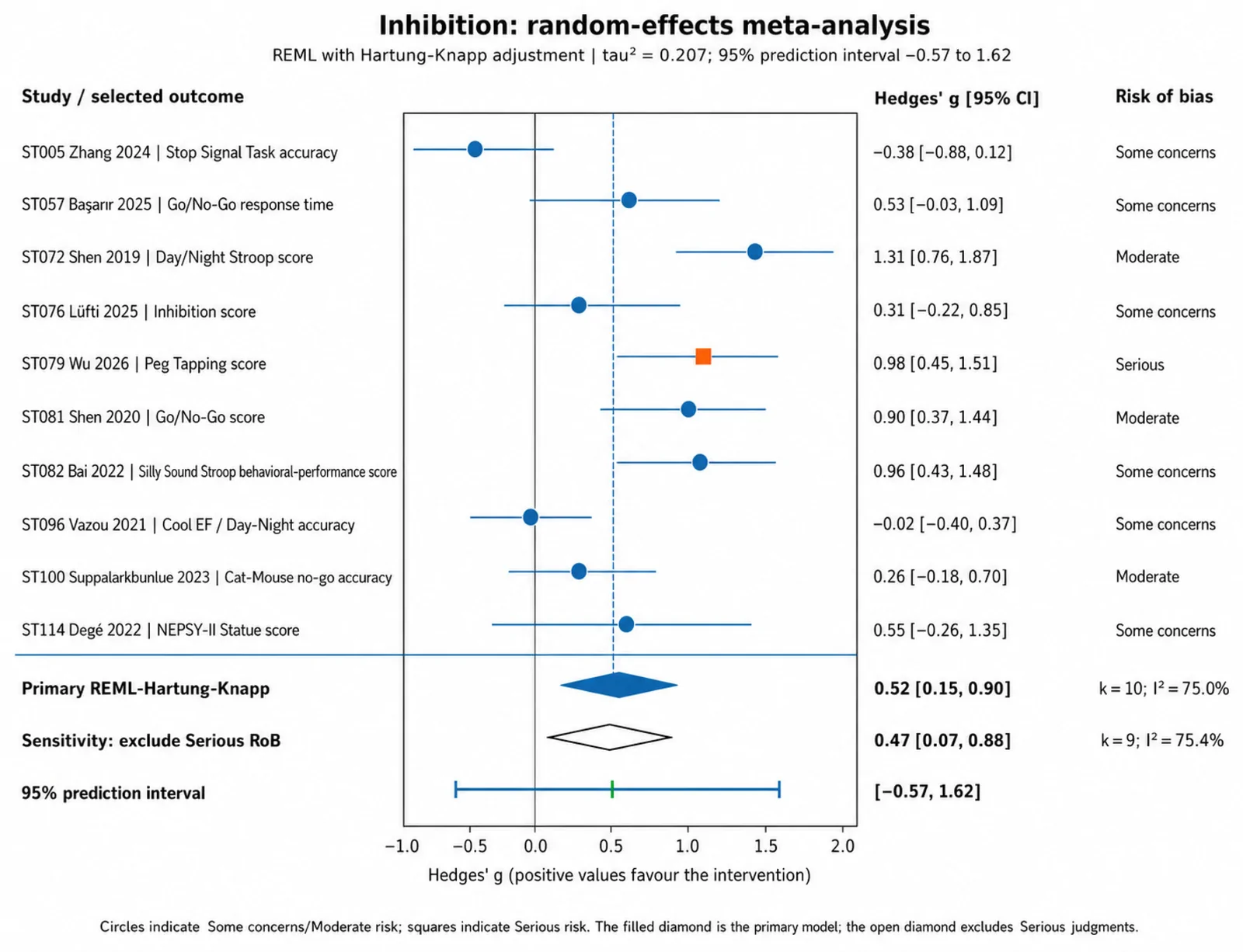
Forest plot for inhibition outcomes. Study labels use final numbered references [13,15,27,28,31,32,35,39,40,44]. Random-effects REML model with Hartung–Knapp adjustment: g = 0.52 (95% CI 0.15 to 0.90), k = 10, I^2^ = 75.0%, τ^2^ = 0.207. The 95% prediction interval was −0.57 to 1.62. The filled diamond shows the primary model; the open diamond excludes the Serious risk-of-bias study.

**Inhibition:** The pooled estimate favoured the intervention (g = 0.52, 95% HKSJ CI 0.15 to 0.90; k = 10; I^2^ = 75.0%; τ^2^ = 0.207), with a 95% prediction interval from −0.57 to 1.62. Excluding the single Serious-risk study retained a favourable estimate (g = 0.47, 95% CI 0.07 to 0.88; k = 9) (Figure 2).

**Figure 3:**
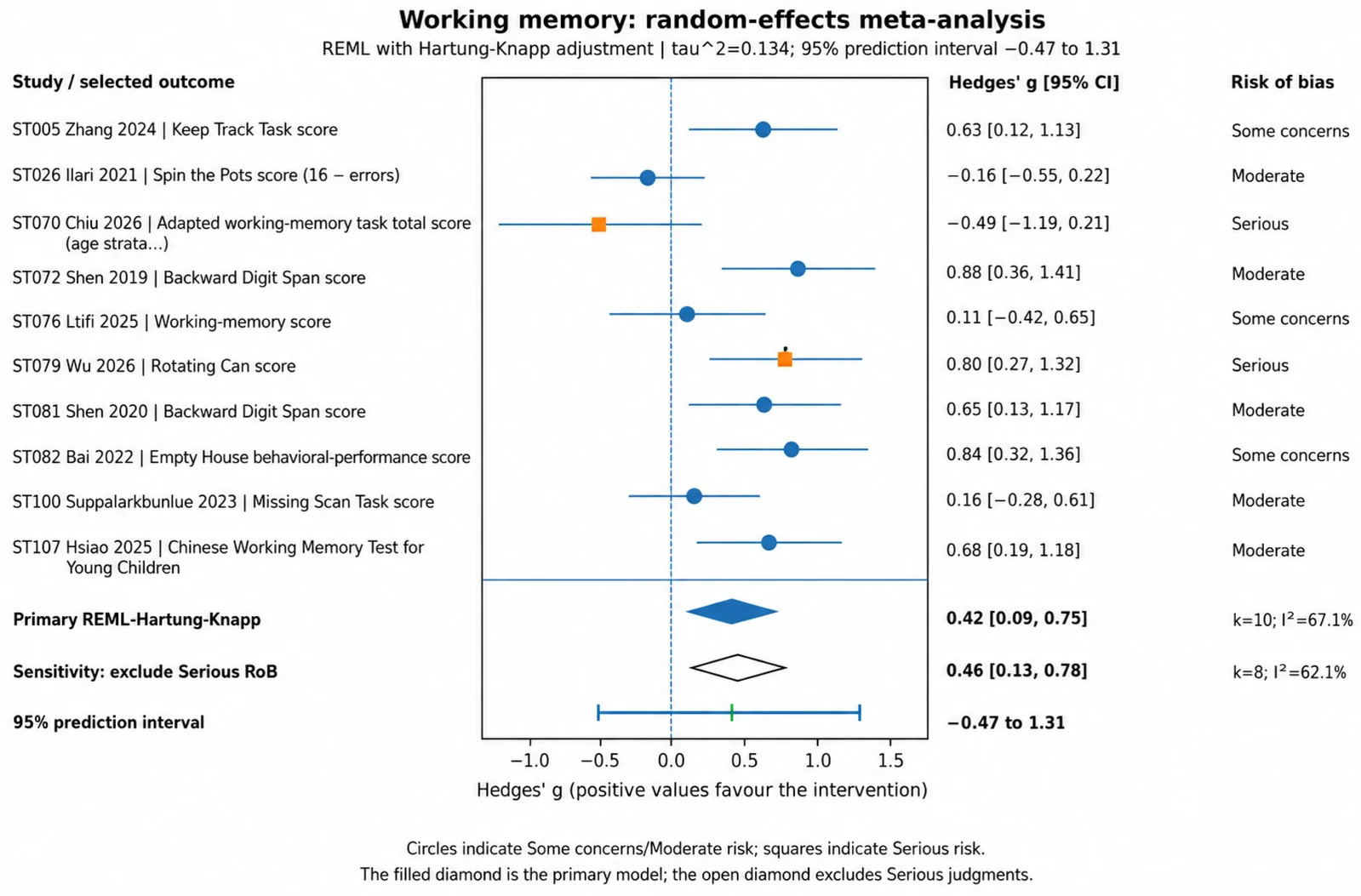
Forest plot for working-memory outcomes. Study labels use final numbered references [13,15,27,31,32,36,38,39,40,42]. Random-effects REML model with Hartung–Knapp adjustment: g = 0.42 (95% CI 0.09 to 0.75), k = 10, I^2^ = 67.1%, τ^2^ = 0.134. The 95% prediction interval was −0.47 to 1.31. The open diamond excludes Serious risk-of-bias studies.

**Working memory:** The pooled estimate favoured the intervention (g = 0.42, 95% HKSJ CI 0.09 to 0.75; k = 10; I^2^ = 67.1%; τ^2^ = 0.134), with a 95% prediction interval from −0.47 to 1.31. Excluding two Serious-risk studies produced g = 0.46 (95% CI 0.13 to 0.78; k = 8) (Figure 3).

**Figure 4:**
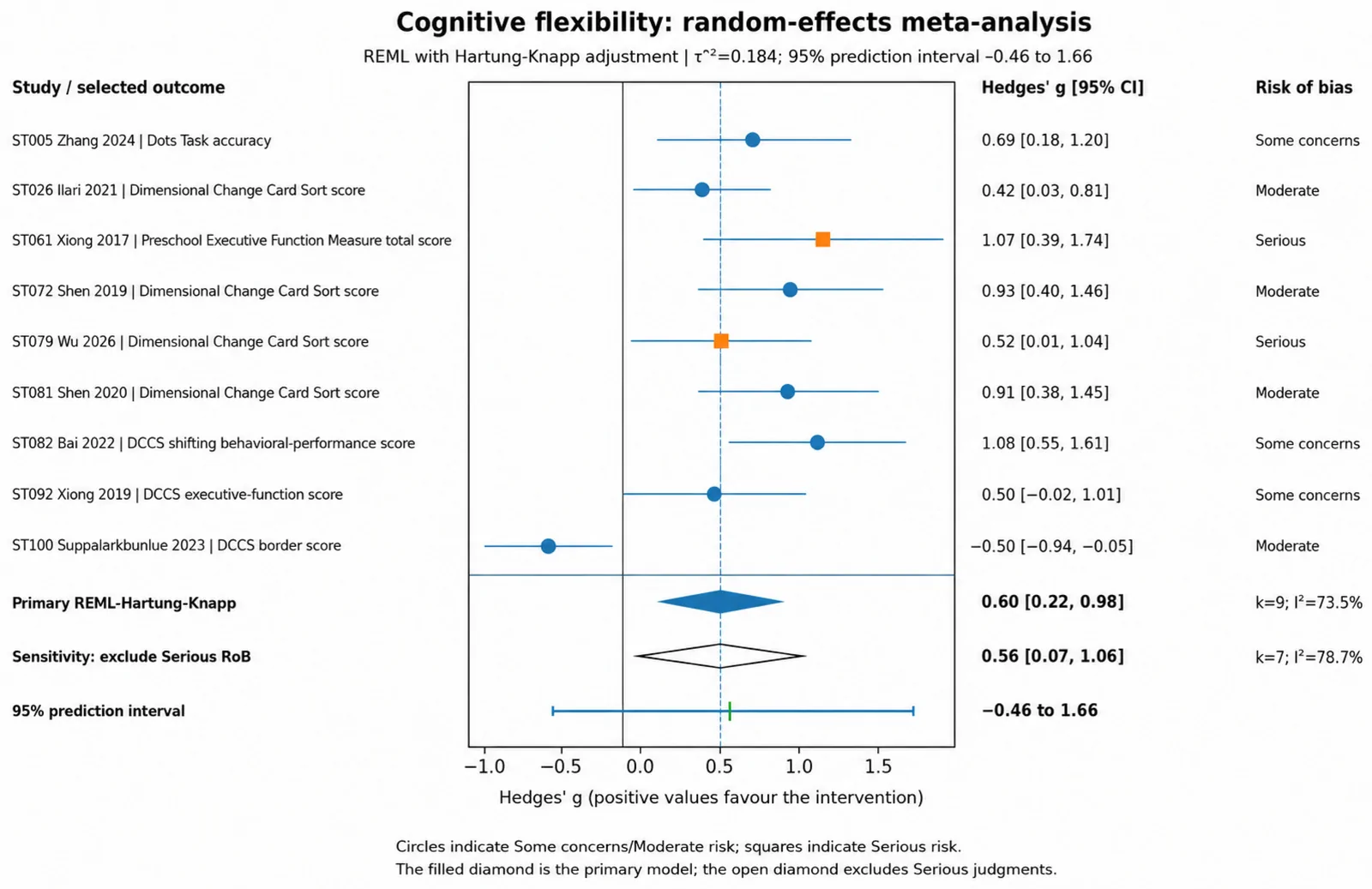
Forest plot for cognitive-flexibility outcomes. Study labels use final numbered references [13,15,27,30,32,33,38,39,40]. Random-effects REML model with Hartung–Knapp adjustment: g = 0.60 (95% CI 0.22 to 0.98), k = 9, I^2^ = 73.5%, τ^2^ = 0.184. The 95% prediction interval was −0.46 to 1.66. The open diamond excludes Serious risk-of-bias studies.

**Cognitive flexibility:** The pooled estimate favoured the intervention (g = 0.60, 95% HKSJ CI 0.22 to 0.98; k = 9; I^2^ = 73.5%; τ^2^ = 0.184), with a 95% prediction interval from −0.46 to 1.66. Excluding two Serious-risk studies retained a favourable but less precise estimate (g = 0.56, 95% CI 0.07 to 1.06; k = 7) (Figure 4). Across all 29 leave-one-study-out analyses, no pooled HKSJ confidence interval crossed the null.

Age sensitivity: Excluding the two younger-inclusive studies (ST076 and ST096) yielded g = 0.63 (95% HKSJ CI 0.18 to 1.08; k = 8) for inhibition; excluding ST076 yielded g = 0.45 (0.09 to 0.82; k = 9) for working memory; cognitive flexibility was unchanged because neither study contributed to that domain. Because ST057 reported a 5–6-year range but a rounded mean of 6.03 years, a separate exclusion sensitivity was conducted and retained a favourable inhibition estimate (g = 0.53, 95% CI 0.10 to 0.95; k = 9).

Unit-of-analysis sensitivity: Eight class- or cluster-assigned studies did not provide a verifiable cluster-adjusted variance for the frozen core outcome. Variance inflation under ICC values of 0.01, 0.05 and 0.10 retained positive HKSJ effects in all domains. Under the most conservative ICC = 0.10 scenario, estimates were g = 0.45 (95% CI 0.08 to 0.81) for inhibition, g = 0.41 (0.09 to 0.72) for working memory and g = 0.53 (0.21 to 0.86) for cognitive flexibility. In an extreme exclusion of all eight affected studies, point estimates remained positive, but confidence intervals crossed the null for inhibition (g = 0.38, −0.26 to 1.02; k = 5) and working memory (g = 0.39, −0.13 to 0.92; k = 5); cognitive flexibility remained favourable (g = 0.52, 0.18 to 0.87; k = 3). Full study-level decisions and scenarios are reported in Supplementary Tables S11 and S12.

**Secondary outcomes:** The secondary intervention outcomes were not suitable for a common quantitative synthesis. Zhang et al. [27] reported mixed hot-executive-function findings: snack-delay performance favoured the intervention, wrapped-gift time favoured the control condition and wrapped-gift strategy was inconclusive. In the Move for Thought study [35], the cluster-adjusted time-by-condition estimate for teacher-rated behavioural control was not statistically significant (adjusted mean difference 0.24, 95% CI −0.04 to 0.52; *p* = 0.094). In the CHAMP trial [34], a random-effects hurdle model indicated greater odds of a non-zero post-test HTKS score in the intervention group (OR 2.98, 95% CI 1.53 to 5.81), whereas cognitive self-regulation outcomes were null. The Bai et al. overall EF behavioural-performance composite [32] averaged z scores from the same three task-based domains already included in the core models and was not treated as independent evidence. The Dot Matrix result reported by Shen et al. [13] was reclassified as visuospatial working memory. Substituting Dot Matrix for Backward Digit Span yielded g = 0.45 (95% HKSJ CI 0.09 to 0.81; k = 10), compared with g = 0.42 (0.09 to 0.75) in the primary working-memory model; the inference was unchanged.

### 3.5. Structured Narrative Synthesis

#### 3.5.1. Other Chronic Intervention Evidence

Twenty-two design-specific units (70 results) could not be meaningfully pooled because interventions, comparators, outcome instruments and reporting formats differed substantially. Among units with some concerns rather than high, serious or critical risk of bias, benefits were confined to selected outcomes, including reaction-time or behavioural inhibition, executive-function growth, executive-control networks, a small inhibition effect, and HTKS [45,46,47,48,49]. In the same priority subset, three trials were broadly null across multiple executive-function domains [50,51,52]. Arts and multicomponent programmes produced selective gains in inhibition, working memory or visuospatial tasks, but other reports found null group-by-time effects or unstable flexibility findings. Large pre-post changes in uncontrolled studies [53,54] and strong findings in severely confounded group comparisons [55,56] were treated as very low-confidence supportive evidence. Overall, chronic non-pooled interventions suggested possible task-specific benefits, especially for inhibition and working memory, but not a consistent improvement across executive-function domains or intervention types.

#### 3.5.2. Acute Independent or Quasi-Experimental Evidence

Five units (19 results) provided limited evidence of immediate benefits. A small block-randomised experiment improved HTKS and cognitive-control errors [57], whereas other studies reported faster go/no-go responses or neural activation without parallel working-memory or flexibility benefits [58], better sorting performance but inconclusive inhibition because of task-specific missingness [59], or a working-memory benefit limited to combined physical and cognitive engagement with post hoc flexibility findings [60]. Indoor versus outdoor free play produced mixed attention, inhibition and self-regulation findings [61]. The pattern was therefore outcome- and condition-specific, with no evidence of a broad acute executive benefit.

#### 3.5.3. Acute Paired or Crossover Evidence

Four units (14 results) were dominated by order, practice, age and subgroup effects. Working-memory differences were order-dependent [62]; one very small study was largely null [63]; active play produced lower Bear/Dragon performance, particularly in younger children [64]; and another crossover study found better DCCS performance and a subgroup-specific reaction-time benefit but null Corsi working memory [65]. These findings did not support a general immediate cognitive advantage; the paired designs require within-person effect methods rather than independent-group Hedges’ g.

#### 3.5.4. Longitudinal Observational Evidence

Nineteen units (74 results) were synthesised separately; 13 had high ROBINS-E and six had some concerns. In the lower-concern subset, most prospective associations were null, small or mixed: movement-guideline adherence yielded limited HTKS or Statue signals [66], sport participation was largely null [67,68], movement-pattern measures were mostly null [69], vigorous activity predicted shifting but sport results were unexpected [70], and low physical activity at age six predicted poorer adolescent working memory while most other associations were null [71]. More favourable patterns in high-concern studies involved active-play opportunities, fitness, motor competence, outdoor activity or lower screen exposure [72,73,74,75,76], but these coexisted with nonlinear, sex-specific and null findings. Overall, prospective evidence supported, at most, context-specific associations between active or motor environments and later executive function; consistency and causal confidence were low.

#### 3.5.5. Cross-Sectional 24-Hour Movement or Guideline Evidence

Twenty units (79 results) did not identify a coherent optimal movement composition. Several lower-concern compositional analyses linked relative MVPA to better inhibition or executive-function ratings [77,78,79] or found composition-level associations with working memory, flexibility or self-regulation [80,81,82]. However, the directions of sleep, sedentary time and light activity coefficients varied, full models were frequently null [83,84], and some traditional and compositional models disagreed. High-concern pilot or guideline studies were largely null or mixed [85,86,87,88,89]. Goldilocks zones and isotemporal substitutions were treated as model-derived predictions rather than causal recommendations.

#### 3.5.6. Cross-Sectional Motor-Competence or Fitness Evidence

Twenty units (70 results) showed the most recurrent observational pattern: higher motor competence or fitness was associated with better inhibition and working memory, with more selective associations for cognitive flexibility and self-regulation. This pattern was present in several adjusted or larger studies [90,91,92,93,94,95] and in age-adjusted task analyses [96]. Nevertheless, findings based on profiles, small strata, sex-specific analyses and mediation models [97,98,99,100,101] were conditional or at high concern for bias; one study was mostly null [102], and one provided indirect pathways only [103]. Thus, motor competence was consistently correlated with executive function, but temporal direction and shared developmental causes could not be resolved.

#### 3.5.7. Cross-Sectional Activity, Sport, Play, Arts or Contextual Evidence

Seventeen units (74 results) were highly heterogeneous. Slot et al. [104] was retained as contextual E2 dramatic/sociodramatic pretend-play evidence because the exposure was operationalised using an adapted scale of dramatic and sociodramatic play quality; it was cross-sectional, was interpreted non-causally and did not contribute to the quantitative core. In the lower-concern subset, pretend play and objective activity were associated with selected self-regulation or executive-function outcomes [104,105], family or child activity was null for working memory [106], sport associations varied by skill type and domain [107], and locomotor-skill benefits coexisted with adverse MVPA associations [108,109]. High-concern arts and sport comparisons showed selective auditory or visuospatial working-memory, inhibition or flexibility advantages [16,110,111,112], but dance was null [113], a music-by-activity interaction was inconclusive [114], and one accelerometry study reported worse inhibition and global executive function with higher MVPA [115]. The evidence therefore supported context-specific associations, not a general executive advantage of participation in sport, play or arts.

### 3.6. Cross-Stream Interpretation

The quantitative intervention core indicated favourable average effects for inhibition, working memory and cognitive flexibility, but all prediction intervals crossed the null, and the non-pooled intervention and observational streams were substantially less consistent. Favourable findings outside the core were usually confined to selected tasks or contexts. Broader behavioural self-regulation and hot executive function were construct- and model-dependent, and the favourable Dot Matrix finding represented an alternative spatial working-memory task rather than an independent visuospatial domain. Motor competence or fitness showed the clearest observational convergence with the intervention evidence, but cross-sectional design and developmental confounding limited mechanistic inference. Acute benefits were not robust across designs, and movement-composition findings did not define a reliable optimal daily allocation. The overall evidence is therefore compatible with context-dependent average executive-function benefits rather than a uniform effect of all movement or arts exposures.

The complete evidence base comprised 130 included reports. Reports not cited individually in the concise main-text synthesis are catalogued in Supplementary Table S1 [116,117,118,119,120,121,122,123,124,125,126,127,128,129,130,131,132,133,134,135,136,137,138,139,140,141,142,143,144,145,146,147,148,149,150,151].

### 3.7. Small-Study Effects and Certainty of Evidence

SMD-specific modified-precision regressions were not statistically notable for inhibition (slope *p* = 0.529) or working memory (*p* = 0.619). Cognitive flexibility was not formally tested because only nine independent effects were available. ROB-ME was judged as Some concerns for all three domains: hard-gate exclusions were based on the inability to recover a unique model-ready effect and variance without assumptions rather than on effect direction or statistical significance, but incomplete model-ready reporting, search-archive limitations and the possibility of unidentified studies prevented a Low-risk judgment. GRADE certainty remained Low for inhibition, working memory and cognitive flexibility, with one-level downgrades for risk of bias and inconsistency. The age and variance-inflation sensitivities did not reverse the direction of the estimates, but the extreme cluster-exclusion analysis materially widened confidence intervals for inhibition and working memory; this unit-of-analysis uncertainty was retained in the certainty interpretation.

## 4. Discussion

### 4.1. Principal Findings

This review integrated 130 reports representing 125 unique studies and separated the evidence into design-specific streams. The corrected primary intervention synthesis comprised 29 effects from 15 unique Study IDs. Average effects favoured the intervention for inhibition, working memory and cognitive flexibility, and all three estimates remained favourable after excluding Serious risk-of-bias judgments. Study-level age auditing showed that no core exposure began before 36 months and only two studies included children younger than 48 months; excluding these studies did not materially weaken the inhibition or working-memory estimates. Variance-inflation scenarios for potentially unadjusted class- or cluster-assigned studies also retained positive effects, although an extreme exclusion of all affected studies substantially reduced precision for inhibition and working memory. The pedagogical audit showed that the quantitative core predominantly represented adult-designed structured programmes, with only three guided-play/structured hybrids and no free-play intervention. Substantial heterogeneity persisted, and every 95% prediction interval crossed the null. The appropriate interpretation is therefore that some structured or guided movement- and arts-based programmes may yield favourable average effects, while the magnitude and even direction expected in a new setting may vary according to content, pedagogy, cognitive demand, comparator, measurement task, age distribution, unit of allocation and study quality.

Evidence for broader self-regulation and hot executive function was construct- and model-dependent. Favourable findings were observed for selected delay-gratification and behavioural-regulation outcomes, but companion outcomes were null or adverse, and the source-preferred analyses ranged from adjusted mean differences to a hurdle-model odds ratio. These results do not support a single pooled global/self-regulation effect. Likewise, the favourable Dot Matrix result represents spatial working memory within a study already contributing another working-memory task, rather than evidence for an independent visuospatial domain.

### 4.2. Interpretation Across Intervention and Exposure Streams

The favourable average effects for inhibition and working memory are compatible with the idea that structured movement experiences can create repeated opportunities to practise rule maintenance, response suppression, updating, sequencing and goal-directed motor control. Many primary interventions involved structured games, coordination demands, motor planning, exergaming, gymnastics, group play or mastery-oriented motor tasks rather than a simple increase in energy expenditure [6,7,8,9,10,11,27,28,29,30,31,32,33,34,35,36,37]. This distinction matters because cognitively engaging activity is not equivalent to low-complexity activity.

Arts-based and mixed movement-arts programmes offer a complementary interpretation. Music, rhythm, dance, drama, music-movement and gesture-based learning can require auditory-motor coupling, sequencing, imitation, turn-taking, action monitoring and flexible rule use [12,13,14,15,16,38,39,40,41,42,43,44]. Nevertheless, the review does not support the claim that any arts exposure is sufficient to improve executive function. Programme content, pedagogical format, cognitive demand, duration, comparator and developmental context varied markedly.

The structured/guided/free-play distinction is therefore consequential. Structured programmes standardised goals, sessions and task sequences; guided-play or hybrid programmes retained child agency within adult-defined constraints; free play was child-directed and lacked a standardised progression. These formats should not be treated as interchangeable, and cognitively engaging should not be used as a synonym for physically active, enjoyable or complex. In the quantitative core, free play was not an intervention category, and only three studies used guided-play/structured hybrids, so the meta-analysis cannot determine whether one pedagogical model is superior. Evidence involving free play outside the core was mixed and context-dependent. The most defensible interpretation is that executive demands embedded in delivery may matter, but the current evidence cannot isolate a causal programme component.

The non-pooled chronic intervention evidence suggested selective and task-specific benefits rather than a consistent improvement across domains [45,46,47,48,49,50,51,52,53,54,55,56]. Acute studies were similarly mixed: some conditions improved selected outcomes, while order, practice, age, subgroup and task-specific effects limited generalisation [57,58,59,60,61,62,63,64,65]. These findings do not establish a broad immediate executive advantage from a single episode of activity or play.

Prospective observational evidence was mostly null, small or mixed in the lower-concern subset, and more favourable findings often came from studies with greater risk-of-bias concerns [66,67,68,69,70,71,72,73,74,75,76]. Cross-sectional 24-hour movement analyses did not identify a coherent optimal daily composition, and model-derived substitutions should not be interpreted as causal prescriptions [77,78,79,80,81,82,83,84,85,86,87,88,89]. Motor competence or fitness showed the clearest observational convergence with inhibition and working memory [90,91,92,93,94,95,96,97,98,99,100,101,102,103], but temporal direction and shared developmental causes remain unresolved. Associations involving sport, play, arts and routine activity were heterogeneous and context-specific [104,105,106,107,108,109,110,111,112,113,114,115].

### 4.3. Heterogeneity, Certainty and Methodological Strengths

Substantial heterogeneity was expected because interventions and exposures differed in content family, dose, cognitive demand, pedagogical format, delivery setting, age distribution, comparator and outcome instrument. Pedagogical differences included the degree of adult direction and child agency, explicit rule or memory demands, task progression, complexity, feedback/scaffolding and implementation fidelity. Programmes carrying similar labels therefore did not necessarily represent equivalent learning experiences. The core contained six individually randomised studies, three cluster-randomised studies and six nonrandomised or quasi-experimental studies; design composition also differed by domain (Table 2). The pooled estimates are average effects across heterogeneous preschool intervention models, not estimates for a single programme or pedagogical approach. Risk of bias further limits inference. Behavioural outcomes are difficult to blind, implementation fidelity and instructional components were unevenly reported, and nonrandomised comparisons remained vulnerable to confounding and selection bias [19,20]. Eight class- or cluster-assigned studies did not provide verifiable adjusted precision for the frozen core effect. ICC-based variance inflation did not reverse the results, but the extreme exclusion analysis showed that precision in inhibition and working memory depends partly on these studies.

Certainty was rated Low for inhibition, working memory and cognitive flexibility because risk of bias and inconsistency materially limited confidence in the magnitude and transportability of the effects [21]. ROB-ME indicated Some concerns for each synthesis. The age audit, design-specific counts and cluster-sensitivity analyses strengthened transparency but did not justify upgrading certainty. The review’s strengths include seven-database coverage, public access to the original protocol, explicit report-to-study consolidation, one selected effect per Study ID/domain/timepoint, a reviewer-facing extraction and outcome-selection register, separation of acute, chronic, longitudinal and cross-sectional designs, prediction intervals, risk-of-bias, age, unit-of-analysis and leave-one-study-out sensitivity analyses, and structured SWiM synthesis without *p*-value vote counting or cross-design pooling [18,26].

### 4.4. Limitations and Implications

The most important limitations are substantial between-study heterogeneity, the inclusion of nonrandomized intervention designs, diverse executive-function tasks and comparators, incomplete model-ready reporting for 15 candidate domain rows and inconsistent reporting of pedagogical components. The structured/guided/free-play and cognitive-engagement coding was added descriptively during revision and depended on the level of detail in the primary reports. Task progression, complexity, teacher scaffolding, child agency and fidelity were frequently partial or not reported, and the sparse categories did not permit a reliable moderator analysis. Although the protocol allowed exposure from birth through 72 months, the quantitative core was concentrated in older preschool samples: no exposure began before 36 months, and only two studies included children younger than 48 months. The findings should therefore not be generalised to infancy or early toddlerhood. The review archive did not preserve every source-specific search execution record, exact per-database yield or a dedicated trial-register/grey-literature search, and 13 reports were retained in a combined not-retrieved/not-assessable category. Although hard-gate exclusions were not selected according to statistical significance or effect direction, missing studies and selective non-reporting cannot be excluded. The small-study diagnostics were conducted at the minimum study count and should not be interpreted as proof that reporting bias is absent. Harmonised post-test Hedges’ g estimates may not fully account for baseline imbalance in nonrandomised studies. Eight class- or cluster-assigned studies lacked verifiable cluster-adjusted variance for the frozen core outcome. ICC scenarios retained positive effects, but the extreme exclusion analysis reduced the number of studies by approximately one half and widened confidence intervals for inhibition and working memory. Consequently, the primary estimates are directionally robust to plausible ICC assumptions, but their precision remains materially uncertain.

The practical interpretation should remain proportionate to the evidence. Some structured and developmentally appropriate movement- or arts-based programmes may support selected executive-function processes, especially inhibition and working memory, but the current evidence does not justify recommending an activity solely on the assumption of a general cognitive benefit or favouring structured intervention, guided play or free play as a universally superior format. Future trials should prospectively specify the pedagogical model and outcome hierarchy; describe task progression, complexity, feedback, teacher guidance and child agency; monitor implementation fidelity; use adequate comparators and transparent cluster adjustment; and report both task-based executive functions and broader self-regulation at repeated follow-up.

## 5. Conclusions

In early childhood, some structured and guided movement- and arts-based interventions may improve inhibition, working memory and cognitive flexibility, on average. These findings should not be interpreted as evidence that movement, play or arts participation per se produces a uniform cognitive benefit. Pedagogical structure and deliberately embedded executive demands may influence outcomes, but the evidence does not identify a superior delivery model. Age-based and plausible ICC sensitivity analyses did not reverse effect direction, yet substantial statistical and pedagogical heterogeneity, risk of bias and unit-of-analysis uncertainty limit confidence in effect magnitude and transportability. Certainty is Low for all three domains. Broader self-regulation/hot-executive-function and spatial working-memory findings are construct- and model-dependent and do not support additional pooled outcome domains.

## Figures and Tables

**Table 1:** Study selection and analytic levels.

Level	n	Comment
Records identified	6126	Across seven databases
Records screened	4306	After deduplication/final clean-up
Reports sought	181	Full-text retrieval target
Reports not retrieved/not assessable	13	Combined archived retrieval-stage category
Reports assessed at full text	168	Eligibility assessment
Reports excluded after full text	38	Reasons in Table S2
Included reports	130	Report-level count
Unique included studies	125	After companion-report consolidation
Study IDs in core intervention meta-analysis	15	At least one verified core-domain effect
Core executive-function effects	29	Inhibition 10; working memory 10; cognitive flexibility 9
Secondary outcome rows retained narratively/sensitivity	4	ST005, ST096, ST109 and ST072
Secondary duplicate composite excluded	1	ST082 overall EF composite

Note: n = number of records, reports, studies or effects at the stated analytic level.

**Table 2:** Study-design composition and developmental coverage of the core quantitative evidence.

Domain	Total k	Individual Randomisation	Cluster Randomisation	Nonrandomised/Quasi-Experimental	Younger-Inclusive Studies
Inhibition	10	5	2	3	2 (ST076, ST096)
Working memory	10	4	1	5	1 (ST076)
Cognitive flexibility	9	3	2	4	0

Note: Design categories refer to the study-level allocation structure verified from the primary reports. Younger-inclusive studies contained participants younger than 48 months; no core exposure began before 36 months.

**Table 3:** Core executive-function meta-analysis, missing-evidence assessment and certainty.

Domain	k	Hedges’ g (95% HKSJ CI)	Heterogeneity and 95% PI	Exclude Serious RoB	ROB-Me/Grade
Inhibition	10	0.52 (0.15 to 0.90)	I^2^ = 75.0%; τ^2^ = 0.207; PI −0.57 to 1.62	0.47 (0.07 to 0.88); k = 9	Some concerns/Low
Working memory	10	0.42 (0.09 to 0.75)	I^2^ = 67.1%; τ^2^ = 0.134; PI −0.47 to 1.31	0.46 (0.13 to 0.78); k = 8	Some concerns/Low
Cognitive flexibility	9	0.60 (0.22 to 0.98)	I^2^ = 73.5%; τ^2^ = 0.184; PI −0.46 to 1.66	0.56 (0.07 to 1.06); k = 7	Some concerns/Low

Note: CI = confidence interval; g = Hedges’ standardized mean difference; HKSJ = Hartung–Knapp–Sidik–Jonkman adjustment; I^2^ = percentage of observed variance attributable to between-study heterogeneity; k = number of independent effects; PI = prediction interval; RoB = risk of bias. Positive g values favour the intervention.

## Data Availability

The data underlying this review are provided in the article and Supplementary Files. The frozen model-ready dataset contains 29 domain-specific effects from 15 unique Study IDs, with arm sample sizes, effect estimates, variances and risk-of-bias fields. The revised Supplementary Package also includes the complete available search documentation, the original-versus-amended protocol log, the study/effect extraction and selection register, study-level age and allocation-unit audits, pedagogical-format and cognitive-engagement coding, ICC-scenario and extreme-exclusion sensitivity results, the study-by-domain missing-evidence matrix, outcome-specific GRADE profile, secondary-outcome adjudication and code required to reproduce the REML–Hartung–Knapp, age, cluster and SMD-specific modified-precision analyses. The original protocol and complete registration history are publicly accessible from PROSPERO at https://www.crd.york.ac.uk/PROSPERO/view/CRD420261363924 (accessed on 9 August 2026). Historical platform exports and source-specific search execution details that were not preserved are not represented as recoverable.

## Supplementary Materials

The following supporting information can be downloaded at: https://www.mdpi.com/article/10.3390/children13091134/s1, PRISMA 2020 Checklist; Supplementary File S1: Registered and database-adapted search strategies with provenance statement; Table S1: Included-report catalogue and study-level traceability for 130 reports representing 125 studies; Table S2: Full-text exclusions (n = 38); Table S3: Study-level consolidation and synthesis allocation; Table S4: Risk of bias for the 15 core quantitative Study IDs; Table S5: Final evidence-role adjudication for candidate intervention studies and secondary outcomes; Table S6: Frozen 29-effect executive-function dataset; Table S7: SMD-specific modified-precision diagnostics; Table S8: ROB-ME evidence matrix and domain assessments; Table S9: GRADE evidence profile and summary of findings; Table S10: Original-versus-amended protocol and decision history; Table S11: Study/effect extraction, age, design, clustering and outcome-selection register; Table S12: Age- and cluster-adjustment sensitivity analyses; Table S13: Pedagogical-format, cognitive-engagement and implementation-characteristics coding of the 15 core interventions; Dataset S1: Model-ready 29-effect CSV; Datasets S2–S4: Age, clustering and sensitivity audit files; Code S1: Reproducible REML–Hartung–Knapp and sensitivity analyses; Code S2: SMD-specific modified-precision analysis; Code S3: Age and cluster-sensitivity analyses. Figure S1A. Inhibition SMD-specific modified-precision diagnostic. Modified slope *p* = 0.529; k = 10. B. Working-memory SMD-specific modified-precision diagnostic. Modified slope *p* = 0.619; k = 10.

## Abbreviations

Abbreviation	Definition
CI	Confidence Interval
DCCS	Dimensional Change Card Sort
EF	Executive Function
GRADE	Grading of Recommendations Assessment, Development and Evaluation
HKSJ	Hartung–Knapp–Sidik–Jonkman
HTKS	Head-Toes-Knees-Shoulders
MVPA	Moderate-to-Vigorous Physical Activity
PI	Prediction Interval
PRISMA	Preferred Reporting Items for Systematic reviews and Meta-Analyses
REML	Restricted Maximum Likelihood
RoB	Risk of Bias
ROB-ME	Risk Of Bias due to Missing Evidence in systematic reviews with meta-analysis
ROBINS-I	Risk Of Bias In Non-randomized Studies of Interventions
ROBINS-E	Risk Of Bias In Non-randomized Studies of Exposures
SWiM	Synthesis Without Meta-analysis
