# Supplementary material for: Early Childhood Movement- and Arts-Based Experiences and Executive Functions: A Systematic Review and Meta-Analysis
Source: Children (Basel). 2026 Aug 24;13(9):1134. doi: 10.3390/children13091134 (PMC13605301; doi:10.3390/children13091134)
Supplement: Supplementary file 1 [file children-13-01134-s001.zip › children-4490133 PRISMA 2020 Checklist corrected.pdf]

PRISMA 2020 Checklist

Early Childhood Movement- and Arts-Based Experiences and Executive Functions: A Systematic Review and Meta-analysis

Children manuscript children-4490133 | PROSPERO CRD420261363924 | Academic Editor revision, 14 August 2026

A. PRISMA 2020 checklist

Section/topic	Item	Checklist item	Status	Location and implementation note
TITLE	1	Identify the report as a systematic review.	Yes	Title, p. 1.
ABSTRACT	2	See the PRISMA 2020 for Abstracts checklist.	Yes	Abstract, pp. 1-2; PRISMA for Abstracts table below.
INTRODUCTION — Rationale	3	Describe the rationale for the review in the context of existing knowledge.	Yes	Introduction, pp. 2-3.
INTRODUCTION — Objectives	4	Provide an explicit statement of the objective(s) or question(s) the review addresses.	Yes	End of Introduction, p. 3.
METHODS — Eligibility criteria	5	Specify inclusion and exclusion criteria and how studies were grouped for the syntheses.	Yes	Methods 2.2, pp. 3-4; Methods 2.9, p. 6; Supplementary Tables S3, S5, S10 and S11; Supplementary Table S13 (pedagogical-format and cognitive-engagement coding).
METHODS — Information sources	6	Specify all databases, registers, websites, organisations, reference lists and other sources searched or consulted, and the date when each source was last searched.	Yes with archive limitation	Methods 2.3, p. 4. Seven databases and inception-through-April-2026 coverage are reported; exact source-specific historical execution dates and per-source yields were not preserved for every database.
METHODS — Search strategy	7	Present the full search strategies for all databases, registers and websites, including filters and limits.	Yes with provenance limitation	Methods 2.3, p. 4; Supplementary File S1, pp. 1-4. The registered historical strategy and seven database-adapted reproducibility reconstructions are presented with an explicit provenance statement.
METHODS — Selection process	8	Specify the methods used to decide whether a study met the inclusion criteria, including how many reviewers screened each record/report, whether they worked independently, and any automation tools used.	Yes	Methods 2.4, p. 4. Zotero/Rayyan, duplicate independent screening and methodological adjudication are reported.
METHODS — Data collection process	9	Specify the methods used to collect data from reports, including how many reviewers collected data, whether they worked independently, author contact and automation methods.	Yes	Methods 2.5, pp. 4-5. Independent duplicate extraction and consensus/third-reviewer procedures are reported.
METHODS — Data items: outcomes	10a	List and define all outcomes for which data were sought and specify whether all compatible results were sought in each outcome domain.	Yes	Methods 2.2 and 2.5, pp. 3-5; Supplementary Tables S3, S5, S6 and S11.
METHODS — Data items: other variables	10b	List and define all other variables for which data were sought and describe assumptions about missing or unclear information.	Yes	Methods 2.5, pp. 4-5; Supplementary Tables S3-S6 and S11; Supplementary Table S13 (pedagogical-format and cognitive-engagement coding).
METHODS — Risk of bias in studies	11	Specify the methods used to assess risk of bias in the included studies, including tool(s), reviewers and any automation tools.	Yes	Methods 2.6, p. 5; Supplementary Table S4, p. 15. RoB 2, ROBINS-I and ROBINS-E are reported.
METHODS — Effect measures	12	Specify the effect measure(s) used for each outcome in the synthesis or presentation of results.	Yes	Methods 2.5, pp. 4-5. Hedges g is used for core outcomes; lower-is-better metrics are reverse-oriented; source-preferred adjusted or non-convertible estimands are retained narratively.
METHODS — Synthesis methods	13a	Describe the processes used to decide which studies were eligible for each synthesis.	Yes	Methods 2.5-2.9, pp. 4-6; Supplementary Tables S5-S6 and S11; Supplementary Table S13 (pedagogical-format and cognitive-engagement coding).

Section/topic	Item	Checklist item	Status	Location and implementation note
METHODS — Synthesis methods	13b	Describe any methods required to prepare the data for presentation or synthesis, such as handling missing summary statistics or data conversions.	Yes	Methods 2.5, 2.7 and 2.8, pp. 4-6; frozen Dataset S1/Table S6; age and clustering audit in Tables S11-S12.
METHODS — Synthesis methods	13c	Describe methods used to tabulate or visually display results of individual studies and syntheses.	Yes	Methods 2.7-2.9, pp. 5-6; Figures 2-4, pp. 9-11; Supplementary Tables S6-S7 and S11-S12.
METHODS — Synthesis methods	13d	Describe methods used to synthesise results and justify model choices; describe methods for statistical heterogeneity.	Yes	Methods 2.7, p. 5; Results 3.4, pp. 8-11; Table 3, p. 8. REML with Hartung-Knapp inference is reported.
METHODS — Synthesis methods	13e	Describe methods used to explore possible causes of heterogeneity.	Yes - descriptive and sensitivity-based	Methods 2.5 and 2.7-2.9; Results 3.2 and 3.4; Discussion 4.3. Developmental age, design and pedagogical heterogeneity were examined descriptively and by sensitivity analyses; sparse pedagogical categories did not support a defensible moderator meta-analysis; Supplementary Table S13.
METHODS — Synthesis methods	13f	Describe sensitivity analyses conducted to assess robustness.	Yes	Methods 2.7-2.8, pp. 5-6; Results 3.4, pp. 8-11; Table 3; Supplementary Table S12, pp. 29-30. Serious-RoB exclusion, 29 leave-one-study-out analyses, task-selection, age and cluster sensitivities are reported.
METHODS — Reporting bias assessment	14	Describe methods used to assess risk of bias due to missing results in a synthesis.	Yes	Methods 2.6-2.7, p. 5; Results 3.7, pp. 13-14; Supplementary Tables S7-S8, pp. 17-21. ROB-ME and SMD-specific modified-precision diagnostics are reported.
METHODS — Certainty assessment	15	Describe methods used to assess certainty/confidence in the body of evidence.	Yes	Methods 2.6, p. 5; Table 3, p. 8; Supplementary Table S9, pp. 21-22. GRADE is applied to the three core outcomes.
RESULTS — Study selection	16a	Describe results of the search and selection process, ideally using a flow diagram.	Yes	Results 3.1, pp. 6-7; Table 1, p. 7; Figure 1, p. 7.
RESULTS — Study selection	16b	Cite studies that might appear to meet inclusion criteria but were excluded, and explain why.	Yes	Supplementary Table S2, pp. 5-8 (38 full-text exclusions and primary reasons).
RESULTS — Study characteristics	17	Cite each included study and present its characteristics.	Yes	Results 3.2, p. 8; Table 2, p. 8; Supplementary Tables S1, S3 and S11, pp. 1-5, 8-14 and 24-28; Supplementary Table S13 (pedagogical-format and cognitive-engagement coding).
RESULTS — Risk of bias in studies	18	Present assessments of risk of bias for each included study.	Yes	Results 3.3, p. 8; Supplementary Table S4, p. 15.
RESULTS — Results of individual studies	19	For all outcomes, present summary statistics for each study and effect estimates with precision.	Yes	Figures 2-4, pp. 9-11; Supplementary Tables S6 and S11, pp. 16-17 and 24-28.
RESULTS — Results of syntheses	20a	For each synthesis, briefly summarise characteristics and risk of bias among contributing studies.	Yes	Results 3.2-3.3, p. 8; Tables 2 and S3-S4; Supplementary Table S13 (pedagogical-format and cognitive-engagement coding).
RESULTS — Results of syntheses	20b	Present results of all statistical syntheses, including effect estimate, precision and heterogeneity; compare groups when relevant.	Yes	Results 3.4, pp. 8-11; Table 3, p. 8; Figures 2-4.
RESULTS — Results of syntheses	20c	Present results of investigations of possible causes of heterogeneity.	Yes - descriptive/sensitivity assessment	Results 3.2 and 3.4, pp. 8-11; Supplementary Table S12, pp. 29-30. The sparse developmental-age distribution did not support a defensible subgroup meta-analysis; age and clustering were examined through prespecified audit rules and sensitivity scenarios; Supplementary Table S13 (pedagogical-format and cognitive-engagement coding).

Section/topic	Item	Checklist item	Status	Location and implementation note
RESULTS — Results of syntheses	20d	Present results of sensitivity analyses.	Yes	Results 3.4, pp. 8-11; Table 3; Supplementary Tables S5-S6 and S12.
RESULTS — Reporting biases	21	Present assessments of risk of bias due to missing results for each synthesis assessed.	Yes	Results 3.7, pp. 13-14; Supplementary Tables S7-S8, pp. 17-21.
RESULTS — Certainty of evidence	22	Present assessments of certainty/confidence for each outcome assessed.	Yes	Table 3, p. 8; Results 3.7, pp. 13-14; Supplementary Table S9, pp. 21-22.
DISCUSSION — Interpretation	23a	Provide a general interpretation of results in the context of other evidence.	Yes	Discussion 4.1-4.3. Interpretation now distinguishes intervention content, pedagogical format and embedded cognitive demands; Supplementary Table S13.
DISCUSSION — Limitations of evidence	23b	Discuss limitations of the evidence included in the review.	Yes	Discussion 4.3-4.4. Limitations include statistical and pedagogical heterogeneity, low certainty and incomplete reporting of progression, guidance, child agency and fidelity.
DISCUSSION — Limitations of review processes	23c	Discuss limitations of the review processes used.	Yes	Discussion 4.4. The pedagogical coding was descriptive, partly post hoc and constrained by incomplete source reporting; no underpowered moderator meta-analysis was conducted.
DISCUSSION — Implications	23d	Discuss implications for practice, policy and future research.	Yes	Discussion 4.4 and Conclusions. Recommendations now emphasise explicit reporting of pedagogical design, task progression, complexity, guidance, child agency, fidelity and clustering.
OTHER — Registration	24a	Provide registration information, including register name and registration number, or state that the review was not registered.	Yes	Methods 2.1, p. 3; PROSPERO CRD420261363924.
OTHER — Protocol	24b	Indicate where the review protocol can be accessed, or state that a protocol was not prepared.	Yes	Methods 2.1, p. 3; public PROSPERO record and protocol link.
OTHER — Amendments	24c	Describe and explain amendments to information provided at registration or in the protocol.	Yes	Methods 2.1, p. 3; version 1.0, post-completion version 2.0 and peer-review minor amendments; Supplementary Table S10, p. 23.
OTHER — Support	25	Describe sources of financial or non-financial support and the role of funders/sponsors.	Yes	Funding statement, p. 17: no external funding.
OTHER — Competing interests	26	Declare competing interests of review authors.	Yes	Conflicts of Interest, p. 17.
OTHER — Availability	27	Report which data, analytic code and other materials are publicly available and where.	Yes with stated archive limits	Supplementary Materials, p. 16; Data Availability, p. 17; Supplementary File S1; Supplementary Tables S1-S12; Datasets S1-S4; Code S1-S3. Unpreserved historical platform exports and source-specific execution details are not represented as recoverable; Supplementary Table S13 (pedagogical-format and cognitive-engagement coding).

B. PRISMA 2020 for Abstracts checklist

Section/topic	Item	Checklist item	Status	Location and implementation note
TITLE	1	Identify the report as a systematic review.	Yes	Title, p. 1.
BACKGROUND — Objectives	2	Provide an explicit statement of the main objective(s) or question(s).	Yes	Abstract, p. 1.

Section/topic	Item	Checklist item	Status	Location and implementation note
METHODS — Eligibility criteria	3	Specify inclusion and exclusion criteria.	Yes	Abstract, p. 1; detailed criteria in Methods 2.2.
METHODS — Information sources	4	Specify information sources and the date when each was last searched.	Reported with archive limitation	Abstract, p. 1: seven databases searched from inception through April 2026. Exact source-specific execution dates were not preserved for every database; the limitation and complete available search documentation are reported in Supplementary File S1.
METHODS — Risk of bias	5	Specify methods used to assess risk of bias in included studies.	Yes	Abstract, p. 1: RoB 2/ROBINS-I, ROB-ME and GRADE; Supplementary Table S13 (pedagogical-format and cognitive-engagement coding).
METHODS — Synthesis of results	6	Specify methods used to present and synthesise results.	Yes	Abstract, p. 1: random-effects REML with Hartung-Knapp adjustment and one effect per Study ID/domain.
RESULTS — Included studies	7	Give the total number of included studies and participants and summarise relevant characteristics.	Yes with non-duplicative domain totals	Abstract, p. 1: 130 reports/125 studies; 15 Study IDs/29 effects; n=675, 697 and 642 by domain. Participant totals are not summed because samples recur across domains.
RESULTS — Synthesis results	8	Present results for main outcomes, preferably with number of studies/participants, effect estimate and precision.	Yes	Abstract, pp. 1-2: k/domain totals, Hedges g with 95% CI, prediction-interval statement, Serious-RoB sensitivity, age/ICC sensitivity, modified-precision diagnostics, ROB-ME and GRADE.
DISCUSSION — Limitations	9	Provide a brief summary of limitations of the evidence.	Yes	Abstract, pp. 1-2: substantial inconsistency, unit-of-analysis uncertainty and risk-of-bias concerns limit confidence.
DISCUSSION — Interpretation	10	Provide a general interpretation of results and important implications.	Yes	Abstract Conclusions, pp. 1-2.
OTHER — Funding	11	Specify the primary source of funding for the review.	Yes	Abstract, p. 2: no external funding.
OTHER — Registration	12	Provide register name and registration number.	Yes	Abstract, p. 1: PROSPERO CRD420261363924.
