# Supplementary material for: Early Childhood Movement- and Arts-Based Experiences and Executive Functions: A Systematic Review and Meta-Analysis
Source: Children (Basel). 2026 Aug 24;13(9):1134. doi: 10.3390/children13091134 (PMC13605301; doi:10.3390/children13091134)
Supplement: Supplementary file 1 [file children-13-01134-s001.zip › children-4490133 Supplementary Material REVISED.pdf]

Supplementary Material

Early Childhood Movement- and Arts-Based Experiences and Executive Functions: A Systematic Review and Meta-analysis

PROSPERO CRD420261363924 | Revised Tables S1-S13

S1 included-report catalogue; S2 full-text exclusions; S3 study-level consolidation; S4 risk of bias; S5 final evidence-role adjudication; S6 frozen 29-effect dataset; S7 SMD-specific diagnostics; S8 ROB-ME; S9 GRADE; S10 protocol amendment history; S11 extraction, age, design and cluster audit; S12 age and clustering sensitivity analyses; S13 pedagogical-format, cognitive-engagement and implementation-characteristics coding.

Supplementary Table S1. Included-report catalogue and study-level traceability

This table lists all 130 included reports using the final main-manuscript reference numbers. Study IDs consolidate companion reports at the project level, while Unit IDs and SWiM cells preserve design-specific synthesis roles. The 130 reports represent 125 unique studies.

Ref.	Report ID	Study ID	Unit ID	SWiM cell	Author/year	Article title	Journal	DOI	Citation role
11	IR108	ST012	ST012-P	A1 Primary chronic intervention meta-analysis	Biino 2023	Cognitively enriched physical activity may foster motor competence and executive function as early as preschool age: a pilot trial	Phys. Educ. Sport Pedagogy	10.1080/17408989.2021.1990249	Main text
13	IR050	ST072	ST072-P	A1 Primary chronic intervention meta-analysis	Shen 2019	Sustained Effect of Music Training on the Enhancement of Executive Function in Preschool Children	Front. Psychol.	10.3389/fpsyg.2019.01910	Main text
14	IR023	ST047	ST047-I	A2 Other chronic intervention evidence	Bentley 2023	A translational application of music for preschool cognitive development: RCT evidence for improved executive function, self-regulation, and school readiness	Dev. Sci.	10.1111/desc.13358	Main text
15	IR084	ST100	ST100-P	A1 Primary chronic intervention meta-analysis	Suppalarkbunlue 2023	Promoting Inhibitory Control in Preschool Children Through Music-Movement Activities in the Classroom	J. Res. Child. Educ.	10.1080/02568543.2022.2111482	Main text
16	IR079	ST099	ST099-X	B4 Cross-sectional activity/sport/play/arts/context evidence	Bayanova 2022	Difference in Executive Functions Development Level between Two Groups: Preschool Children Who Took Extra Music Classes in Art Schools and Children Who Took Only General Music and Dance Classes Offered by Preschools	Educ. Sci.	10.3390/educsci12020119	Main text
27	IR005	ST005	ST005-P	A1 Primary chronic intervention meta-analysis	Zhang 2024	Recreational gymnastics exercise of moderate intensity enhances executive function in Chinese preschoolers: A randomized controlled trial	PsyCh J.	10.1002/pchj.786	Main text
28	IR034	ST057	ST057-P	A1 Primary chronic intervention meta-analysis	Başarır 2025	Effects of coordination-based training on preschool children's physical fitness, motor competence and inhibition control	BMC Pediatr.	10.1186/s12887-025-05897-x	Main text
29	IR037	ST060	ST060-P	A1 Primary chronic intervention meta-analysis	He 2025	Effects of 12-week aerobic exercise with different frequencies on executive function in preschool children: a cluster randomized controlled trial	Psychol. Health	10.1080/08870446.2024.2309542	Main text
30	IR038	ST061	ST061-P	A1 Primary chronic intervention meta-analysis	Xiong 2017	Effects of Structured Physical Activity Program on Chinese Young Children's Executive Functions and Perceived Physical Competence in a Day Care Center	Biomed Res. Int.	10.1155/2017/5635070	Main text
31	IR055	ST076	ST076-P	A1 Primary chronic intervention meta-analysis	Ltifi 2025	Mini-Trampoline Training Enhances Executive Functions and Motor Skills in Preschoolers	Children	10.3390/children12101405	Main text
32	IR061	ST082	ST082-P	A1 Primary chronic intervention meta-analysis	Bai 2022	Effects of Group-Play Moderate to Vigorous Intensity Physical Activity Intervention on Executive Function and Motor Skills in 4- to 5-Year-Old Preschoolers: A Pilot Cluster Randomized Controlled Trial	Front. Psychol.	10.3389/fpsyg.2022.847785	Main text
33	IR071	ST092	ST092-P	A1 Primary chronic intervention meta-analysis	Xiong 2019	Effects of Exergaming on Preschoolers' Executive Functions and Perceived Competence: A Pilot Randomized Trial	J. Clin. Med.	10.3390/jcm8040469	Main text
34	IR089	ST109	ST109-P	A1 Primary chronic intervention meta-analysis	Miller 2023	Mastery-oriented motor competence intervention improves behavioral but not cognitive self-regulation in head start preschoolers: Randomized controlled trial results	Scand. J. Med. Sci. Sports	10.1111/sms.14294	Main text
35	IR076	ST096	ST096-P	A1 Primary chronic intervention meta-analysis	Vazou 2021	Cognitively Engaging Physical Activity for Targeting Motor, Cognitive, Social, and Emotional Skills in the Preschool Classroom: The Move for Thought preK-K Program	Front. Psychol.	10.3389/fpsyg.2021.729272	Main text
36	IR048	ST070	ST070-P	A1 Primary chronic intervention meta-analysis	Chiu 2026	Effects of an 8-Week Programmed Physical Activity Intervention on Children's Cognitive, Emotional, and Body Movement Development—A Quasi-Experimental Study of a Preschool in Taiwan	Children	10.3390/children13030319	Main text
37	IR077	ST097	ST097-P	A1 Primary chronic intervention meta-analysis	Díaz-Quesada 2025	Effects of a physical activity programme using active methodology on physical and cognitive parameters in children under 6 years of age	Sport Soc.	10.1080/17430437.2025.2539823	Main text

Ref.	Report ID	Study ID	Unit ID	SWiM cell	Author/year	Article title	Journal	DOI	Citation role
38	IR122	ST026	ST026-P	A1 Primary chronic intervention meta-analysis	Ilari 2021	Musical Activities, Prosocial Behaviors, and Executive Function Skills of Kindergarten Children	Music Sci.	10.1177/20592043211054829	Main text
39	IR060	ST081	ST081-P	A1 Primary chronic intervention meta-analysis	Shen 2020	Promotion of Street-Dance Training on the Executive Function in Preschool Children	Front. Psychol.	10.3389/fpsyg.2020.585598	Main text
40	IR058	ST079	ST079-P	A1 Primary chronic intervention meta-analysis	Wu 2026	Differential effects of child-appropriate music styles on executive function in preschool children: a longitudinal experimental study	Front. Psychol.	10.3389/fpsyg.2026.1779286	Main text
41	IR078	ST098	ST098-P	A1 Primary chronic intervention meta-analysis	Çiftçi 2022	The effect of creative drama activities in early childhood on the executive functions of children	Education 3-13	10.1080/03004279.2020.1849343	Main text
42	IR087	ST107	ST107-P	A1 Primary chronic intervention meta-analysis	Hsiao 2025	Using gesture recognition with the memory strategy to improve preschoolers' learning performance, motor skills, and executive function	Educ. Technol. Res. Dev.	10.1007/s11423-025-10471-4	Main text
43	IR093	ST113	ST113-P	A1 Primary chronic intervention meta-analysis	Bugos 2017	The effects of a short-term music program on preschool children's executive functions	Psychol. Music	10.1177/0305735617692666	Main text
44	IR094	ST114	ST114-P	A1 Primary chronic intervention meta-analysis	Degé 2022	The Influence of Music Training on Motoric Inhibition in German Preschool Children	Music. Sci.	10.1177/1029864920938432	Main text
45	IR003	ST003	ST003-I	A2 Other chronic intervention evidence	Giordano 2022	Does Physical Activity Improve Inhibition in Kindergarteners? A Pilot Study	Percept. Mot. Skills	10.1177/00315125221109216	Main text
46	IR114	ST018	ST018-I	A2 Other chronic intervention evidence	Capio 2024	The contributions of motor skill proficiency to cognitive and social development in early childhood	Sci. Rep.	10.1038/s41598-024-79538-1	Main text
47	IR016	ST040	ST040-I	A2 Other chronic intervention evidence	Li et al. 2025	The influence of Chinese martial arts teaching intervention based on embodied cognition theory on attention networks in 5-6-year-old children	J. Exp. Child Psychol.	10.1016/j.jecp.2025.106316	Main text
48	IR019	ST043	ST043-I	A2 Other chronic intervention evidence	Olive et al. 2024	Physical activity intervention improves executive function and language development during early childhood: The Active Early Learning cluster randomized controlled trial	Child Dev.	10.1111/cdev.14014	Main text
49	IR026	ST049	ST049-I	A2 Other chronic intervention evidence	Mulvey 2018	Head, Toes, Knees, SKIP! Improving Preschool Children's Executive Function Through a Motor Competence Intervention	J. Sport Exerc. Psychol.	10.1123/jsep.2018-0007	Main text
50	IR011	ST035	ST035-I	A2 Other chronic intervention evidence	Mavilidi et al. 2023	Effects of Cognitively Engaging Physical Activity on Preschool Children's Cognitive Outcomes	Res. Q. Exerc. Sport	10.1080/02701367.2022.2059435	Main text
51	IR015	ST039	ST039-I	A2 Other chronic intervention evidence	Wen et al. 2018	Effect of Mini-Trampoline Physical Activity on Executive Functions in Preschool Children	BioMed Res. Int.	10.1155/2018/2712803	Main text
52	IR040	ST063	ST063-I	A2 Other chronic intervention evidence	Buene Vabø 2025	Effects of preschool staff professional development in physical activity on self-regulation, executive function and early academic learning in 3-5-year-old children: the active learning Norwegian preschool(er)s (ACTNOW) cluster-randomized controlled trial	J. Sports Sci.	10.1080/02640414.2025.2460886	Main text
53	IR118	ST022	ST022-I	A2 Other chronic intervention evidence	García Núñez 2024	Unlocking abilities: enhancing executive skills through play and movement in preschool physical education	J. Phys. Educ. Sport	10.7752/jpes.2024.01021	Main text
54	IR121	ST025	ST025-I	A2 Other chronic intervention evidence	Chaiakaraphong 2021	Development of musical experiential model to enhance executive functions of early childhood	Kasetsart J. Soc. Sci.	10.34044/j.kjss.2021.42.3.17	Main text
55	IR046	ST068	ST068-I	A2 Other chronic intervention evidence	Brian 2026	Examining the Impact of Young Children's Motor Development on Inhibitory Control and Social Skills	Children	10.3390/children13030318	Main text
56	IR099	ST118	ST118-I	A2 Other chronic intervention evidence	Zach 2015	The effect of physical activity on spatial perception and attention in early childhood	Cogn. Dev.	10.1016/j.cogdev.2015.08.003	Main text
57	IR022	ST046	ST046-I	A3 Acute independent/quasi-experimental evidence	Ureña et al. 2020	Acute Effect of Cognitive Compromise during Physical Exercise on Self-Regulation in Early Childhood Education	Int. J. Environ. Res. Public Health	10.3390/ijerph17249325	Main text
58	IR062	ST083	ST083-I	A3 Acute independent/quasi-experimental evidence	Du 2025	The Effects of Acute Cognitively Engaging Physical Activity on Executive Function in Preschool Children: Evidence from Behavioral and fNIRS Measures	Behav. Sci.	10.3390/bs15121712	Main text
59	IR080	ST101	ST101-I	A3 Acute independent/quasi-experimental evidence	Antrilli 2018	Toddlers on touchscreens: immediate effects of gaming and physical activity on cognitive flexibility of 2.5-year-olds in the US	J. Child. Media	10.1080/17482798.2018.1486332	Main text
60	IR097	ST116	ST116-I	A3 Acute independent/quasi-experimental evidence	Preston 2025	Cognitively engaging physical activity has an immediate impact on preschool children's executive function	Early Child. Res. Q.	10.1016/j.ecresq.2025.02.008	Main text
61	IR001	ST001	ST001-I	A3 Acute independent/quasi-experimental evidence	Koepp 2022	Preschoolers' executive functions following indoor and outdoor free play	Trends Neurosci. Educ.	10.1016/j.tine.2022.100182	Main text
62	IR064	ST085	ST085-I	A4 Acute paired/crossover evidence	Fitamen 2021	Play First Before Doing Your Exercise: Does Acting in a Game-Like Task Improve 5-Year-Olds' Working Memory Performance?	Front. Psychol.	10.3389/fpsyg.2021.659020	Main text
63	IR098	ST117	ST117-I	A4 Acute paired/crossover evidence	Rosiek 2022	A Comparison of the Effects of Outdoor Physical Activity and Indoor Classroom-Based Activities on Measures of Executive Function in Preschoolers	Int. J. Early Child.	10.1007/s13158-022-00318-x	Main text
64	IR103	ST122	ST122-I	A4 Acute paired/crossover evidence	Tandon 2018	Short term impact of physical activity vs. sedentary behavior on preschoolers' cognitive functions	Ment. Health Phys. Act.	10.1016/j.mhpa.2018.06.004	Main text
65	IR104	ST123	ST123-I	A4 Acute paired/crossover evidence	Zhang 2020	Differential effects of acute physical activity on executive function in preschoolers with high and low habitual physical activity levels	Ment. Health Phys. Act.	10.1016/j.mhpa.2020.100326	Main text

Ref.	Report ID	Study ID	Unit ID	SWiM cell	Author/year	Article title	Journal	DOI	Citation role
66	IR007	ST007	ST007-L	B1 Longitudinal observational evidence	Taylor 2021	Adherence to 24-h movement behavior guidelines and psychosocial functioning in young children: a longitudinal analysis	Int. J. Behav. Nutr. Phys. Act.	10.1186/s12966-021-01185-w	Main text
67	IR110	ST014	ST014-L	B1 Longitudinal observational evidence	Howard 2018	Children's sports participation and self-regulation: Bi-directional longitudinal associations	Early Child. Res. Q.	10.1016/j.ecresq.2017.09.006	Main text
68	IR090	ST110	ST110-L	B1 Longitudinal observational evidence	Bryant 2024	Testing Longitudinal Relations among Preschool Sport and Kindergarten Executive Function and Academic Outcomes	J. Child Fam. Stud.	10.1007/s10826-023-02671-4	Main text
69	IR117	ST021	ST021-L	B1 Longitudinal observational evidence	Verswijveren 2020	Longitudinal associations of sedentary time and physical activity duration and patterns with cognitive development in early childhood	Ment. Health Phys. Act.	10.1016/j.mhpa.2020.100340	Main text
70	IR027	ST050	ST050-L	B1 Longitudinal observational evidence	McNeill 2020	Longitudinal associations of physical activity and modified organized sport participation with executive function and psychosocial health in preschoolers	J. Sports Sci.	10.1080/02640414.2020.1803037	Main text
71	IR035	ST058	ST058-L	B1 Longitudinal observational evidence	López-Vicente 2017	Are Early Physical Activity and Sedentary Behaviors Related to Working Memory at 7 and 14 Years of Age?	J. Pediatr.	10.1016/j.jpeds.2017.05.079	Main text
72	IR036	ST059	ST059-L	B1 Longitudinal observational evidence	Becker 2025	Connections among family socioeconomic status, aerobic fitness, executive function, and the positive experiences of childhood physical activity	J. Exp. Child Psychol.	10.1016/j.jecp.2024.106147	Main text
73	IR041	ST064	ST064-L	B1 Longitudinal observational evidence	Li 2026	Outdoor physical activity from birth to preschool and its independent and interactive associations with children's executive function and behavioral problems	Eur. Child Adolesc. Psychiatry	10.1007/s00787-025-02836-0	Main text
74	IR067	ST088	ST088-L	B1 Longitudinal observational evidence	Tandon 2020	The relationship of gross motor and physical activity environments in child care settings with early learning outcomes	Early Child Dev. Care	10.1080/03004430.2018.1485670	Main text
75	IR082	ST103	ST103-L	B1 Longitudinal observational evidence	C. Liu 2026	Infant Motor Competence Predicts Executive Functions in Preschoolers: The Role of Sleep	Behav. Sci.	10.3390/bs16020288	Main text
76	IR086	ST106	ST106-L	B1 Longitudinal observational evidence	Chou 2022	Chinese preschool children's physical fitness, motor competence, executive functioning, and receptive language, math, and science performance in Kindergarten	Child. Youth Serv. Rev.	10.1016/j.childyouth.2022.106397	Main text
77	IR004	ST004	ST004-X	B2 Cross-sectional 24-hour movement/guideline evidence	Lau 2024	24-Hour movement behaviors and executive functions in preschoolers: A compositional and isotemporal reallocation analysis	Child Dev.	10.1111/cdev.14013	Main text
78	IR017	ST041	ST041-X	B2 Cross-sectional 24-hour movement/guideline evidence	Bezerra et al. 2021	24-hour movement behaviour and executive function in preschoolers: a compositional and isotemporal reallocation analysis	Eur. J. Sport Sci.	10.1080/17461391.2020.1795274	Main text
79	IR033	ST056	ST056-X	B2 Cross-sectional 24-hour movement/guideline evidence	Chang 2025	Relationship between 24-hour movement behaviors and executive function in preschool children based on compositional data analysis	Acta Psychol.	10.1016/j.actpsy.2025.105394	Main text
80	IR012	ST036	ST036-X	B2 Cross-sectional 24-hour movement/guideline evidence	Batista Albuquerque Goulart Lemos et al. 2026	24h movement behaviors and self-regulation in preschoolers: Cross-sectional associations using compositional and isotemporal analyses	Psychol. Sport Exerc.	10.1016/j.psychsport.2025.103002	Main text
81	IR020	ST044	ST044-X	B2 Cross-sectional 24-hour movement/guideline evidence	Kuzik et al. 2020	Movement behaviours and physical, cognitive, and social-emotional development in preschool-aged children: Cross-sectional associations using compositional analyses	PLoS ONE	10.1371/journal.pone.0237945	Main text
82	IR028	ST051	ST051-X	B2 Cross-sectional 24-hour movement/guideline evidence	Zahran 2024	Optimal levels of sleep, sedentary behaviour, and physical activity needed to support cognitive function in children of the early years	BMC Pediatr.	10.1186/s12887-024-05186-z	Main text
83	IR010	ST010	ST010-X	B2 Cross-sectional 24-hour movement/guideline evidence	Abdeta 2025	Associations Between 24-h Movement Behaviours and Health in 3- and 4-Year-Old Children From a Low-Income Country: The SUNRISE Ethiopia Study	Child Care Health Dev.	10.1111/cch.70096	Main text
84	IR042	ST065	ST065-X	B2 Cross-sectional 24-hour movement/guideline evidence	Byambaa 2026	Compositional analysis of the associations between 24-hour movement behaviours, motor skills, and executive function in Mongolian preschool children	J. Sports Sci.	10.1080/02640414.2025.2574192	Main text
85	IR115	ST019	ST019-X	B2 Cross-sectional 24-hour movement/guideline evidence	Carson 2017	Associations of subjectively and objectively measured sedentary behavior and physical activity with cognitive development in the early years	Ment. Health Phys. Act.	10.1016/j.mhpa.2017.05.003	Main text
86	IR123	ST027	ST027-X	B2 Cross-sectional 24-hour movement/guideline evidence	Tanaka 2020	Relationship between adherence to WHO 24-Hour Movement Guidelines for the Early Years and motor skills or cognitive function in preschool children: SUNRISE pilot study	Jpn. J. Phys. Fit. Sports Med.	10.7600/jspfsm.69.327	Main text
87	IR045	ST067	ST067-X	B2 Cross-sectional 24-hour movement/guideline evidence	Draper 2020	Understanding the influence of 24-hour movement behaviours on the health and development of preschool children from low-income South African settings: the SUNRISE pilot study	S. Afr. J. Sports Med.	10.17159/2078-516x/2020/v32i1a8415	Main text
88	IR056	ST077	ST077-X	B2 Cross-sectional 24-hour movement/guideline evidence	Munambah 2021	24 hour movement behaviours and the health and development of pre-school children from Zimbabwean settings: the SUNRISE pilot study	S. Afr. J. Sports Med.	10.17159/2078-516x/2021/v33i1a10864	Main text
89	IR074	ST094	ST094-X	B2 Cross-sectional 24-hour movement/guideline evidence	Li 2025	Mediating Effects of Executive Function on the Relationship between Sleep Problems and Emotional and Behavioral Problems among Preschoolers: Physical Activity as a Protective Factor	Child Psychiatry Hum. Dev.	10.1007/s10578-025-01849-9	Main text
90	IR119	ST023	ST023-X	B3 Cross-sectional motor competence/fitness evidence	Zhang 2024	The Association Between Motor Competence and Inhibitory Control in Preschool Children	Children	10.3390/children11121537	Main text
91	IR047	ST069	ST069-X	B3 Cross-sectional motor competence/fitness evidence	Han 2022	Association between fundamental motor skills and executive function in preschool children: A cross-sectional study	Front. Psychol.	10.3389/fpsyg.2022.978994	Main text
92	IR065	ST086	ST086-X	B3 Cross-sectional motor competence/fitness evidence	Luo 2023	Association of physical activity and fitness with executive function among preschoolers	Int. J. Clin. Health Psychol.	10.1016/j.ijchp.2023.100400	Main text

Ref.	Report ID	Study ID	Unit ID	SWiM cell	Author/year	Article title	Journal	DOI	Citation role
93	IR073	ST093	ST093-X	B3 Cross-sectional motor competence/fitness evidence	Liu 2022	Relationship Between Gross Motor Skills and Inhibitory Control in Preschool Children: A Pilot Study	Front. Hum. Neurosci.	10.3389/fnhum.2022.848230	Main text
94	IR092	ST112	ST112-X	B3 Cross-sectional motor competence/fitness evidence	Kong 2025	The relationship between fundamental motor skills and executive function in children: a stratified analysis by age and sex	Front. Psychol.	10.3389/fpsyg.2025.1669915	Main text
95	IR101	ST120	ST120-X	B3 Cross-sectional motor competence/fitness evidence	Veldman 2023	Associations of gross motor skills with self-regulation and executive function in preschool-aged children	Australas. J. Early Child.	10.1177/18369391231175524	Main text
96	IR100	ST119	ST119-X	B3 Cross-sectional motor competence/fitness evidence	Maurer 2019	Towards a better understanding of the association between motor skills and executive functions in 5- to 6-year-olds: The impact of motor task difficulty	Hum. Mov. Sci.	10.1016/j.humov.2019.06.010	Main text
97	IR013	ST037	ST037-X	B3 Cross-sectional motor competence/fitness evidence	Fernandes et al. 2022	Association between executive functions and gross motor skills in overweight/obese and eutrophic preschoolers: cross-sectional study	BMC Pediatr.	10.1186/s12887-022-03553-2	Main text
98	IR039	ST062	ST062-X	B3 Cross-sectional motor competence/fitness evidence	Starrett 2026	Examining the Relationship Between Actual and Perceived Motor Competence Profiles With Executive Function	Res. Q. Exerc. Sport	10.1080/02701367.2025.2557377	Main text
99	IR075	ST095	ST095-X	B3 Cross-sectional motor competence/fitness evidence	Chichinina 2025	Thinking, Feeling, and Moving in Kindergarten Children: How Motor Competence Shapes Executive Function Skills and Emotion Comprehension in Girls	Children	10.3390/children12101381	Main text
100	IR081	ST102	ST102-X	B3 Cross-sectional motor competence/fitness evidence	J. Liu 2026	Motor coordination mediates the relationship between physical activity and executive function among preschoolers	Int. J. Sport Exerc. Psychol.	10.1080/1612197x.2025.2487881	Main text
101	IR095	ST115	ST115-X	B3 Cross-sectional motor competence/fitness evidence	Li 2025	Mediation of executive functions in the relationship between motor skills and psychosocial health in preschool children	J. Exerc. Sci. Fit.	10.1016/j.jesf.2025.04.002	Main text
102	IR091	ST111	ST111-X	B3 Cross-sectional motor competence/fitness evidence	Capio 2023	Association of working memory with gross motor skills in early childhood	Int. J. Sport Exerc. Psychol.	10.1080/1612197x.2022.2098355	Main text
103	IR105	ST124	ST124-X	B3 Cross-sectional motor competence/fitness evidence	Zhang 2018	Motor Skills and Executive Function Contribute to Early Achievement in East Asia and the Pacific	Early Educ. Dev.	10.1080/10409289.2018.1510204	Main text
104	IR129	ST033	ST033-X	B4 Cross-sectional activity/sport/play/arts/context evidence	Slot 2017	Preschoolers' cognitive and emotional self-regulation in pretend play: Relations with executive functions and quality of play	Infant Child Dev.	10.1002/icd.2038	Main text
105	IR059	ST080	ST080-X	B4 Cross-sectional activity/sport/play/arts/context evidence	Wang 2025	Effects of physical activity on executive function and its subdomains in children aged 5–6	Front. Psychol.	10.3389/fpsyg.2025.1651806	Main text
106	IR057	ST078	ST078-X	B4 Cross-sectional activity/sport/play/arts/context evidence	Aguayo 2021	Association of Family Nutrition and Physical Activity with Preschooler's Working Memory: A Cross-Sectional Study among Mexican Children	Children	10.3390/children8060506	Main text
107	IR085	ST105	ST105-X	B4 Cross-sectional activity/sport/play/arts/context evidence	Bryant 2021	The Cognitive Benefits of Participating in Structured Sports for Preschoolers	Early Educ. Dev.	10.1080/10409289.2020.1799619	Main text
108	IR025	ST016	ST016-X	B4 Cross-sectional activity/sport/play/arts/context evidence	Cook 2019	Associations of physical activity and gross motor skills with executive function in preschool children from low-income South African settings	Dev. Sci.	10.1111/desc.12820	Main text
109	IR112	ST016	ST016-X	B4 Cross-sectional activity/sport/play/arts/context evidence	Cook 2021	Associations between South African preschoolers' routine physical activity, self-regulation and psychosocial well-being	Ment. Health Phys. Act.	10.1016/j.mhpa.2021.100383	Main text
110	IR006	ST006	ST006-X	B4 Cross-sectional activity/sport/play/arts/context evidence	Strait 2012	Musical training during early childhood enhances the neural encoding of speech in noise	Brain Lang.	10.1016/j.bandl.2012.09.001	Main text
111	IR124	ST028	ST028-X	B4 Cross-sectional activity/sport/play/arts/context evidence	Dolgikh 2022	The Relationship between Teacher Evaluation of Children's Musical Abilities and Executive Functions Indicators in Preschool Age	Russ. Psychol. J.	10.21702/rpj.2022.4.5	Main text
112	IR063	ST084	ST084-X	B4 Cross-sectional activity/sport/play/arts/context evidence	Yakushina 2025	Football training vs. martial arts training: How are they related to executive function skills in 5-6-year-old boys?	Front. Psychol.	10.3389/fpsyg.2025.1668266	Main text
113	IR083	ST104	ST104-X	B4 Cross-sectional activity/sport/play/arts/context evidence	Chichinina 2022	The Relationship between Executive Functions and Dance Classes in Preschool Age Children	Educ. Sci.	10.3390/educsci12110788	Main text
114	IR053	ST075	ST075-X	B4 Cross-sectional activity/sport/play/arts/context evidence	Lucendo-Noriega 2026	Music and physical activity in early childhood: the ambiguous role of the at-home context and extracurricular activities	Front. Psychol.	10.3389/fpsyg.2026.1729705	Main text
115	IR088	ST108	ST108-X	B4 Cross-sectional activity/sport/play/arts/context evidence	Willoughby 2018	Testing the association between physical activity and executive function skills in early childhood	Early Child. Res. Q.	10.1016/j.ecresq.2018.03.004	Main text
116	IR002	ST002	ST002-I	A2 Other chronic intervention evidence	Linnavalli 2018	Music playschool enhances children's linguistic skills	Sci. Rep.	10.1038/s41598-018-27126-5	Main-text index + Supplementary Table S1

Ref.	Report ID	Study ID	Unit ID	SWiM cell	Author/year	Article title	Journal	DOI	Citation role
117	IR008	ST008	ST008-L	B1 Longitudinal observational evidence	Yang 2024	Daily Physical Activity, Sports Participation, and Executive Function in Children	JAMA Netw. Open	10.1001/jamanetworkopen.2024.49879	Main-text index + Supplementary Table S1
118	IR009	ST009	ST009-I	A2 Other chronic intervention evidence	Zhang 2022	Physical activity intervention promotes working memory and motor competence in preschool children	Front. Public Health	10.3389/fpubh.2022.984887	Main-text index + Supplementary Table S1
119	IR014	ST038	ST038-I	A2 Other chronic intervention evidence	Kosokabe et al. 2021	Self-directed dramatic and music play programs enhance executive function in Japanese children	Trends Neurosci. Educ.	10.1016/j.tine.2021.100158	Main-text index + Supplementary Table S1
120	IR018	ST042	ST042-I	A2 Other chronic intervention evidence	Kosokabe et al. 2025	Self-directed music play to improve executive function in young children using NIRS	Sci. Rep.	10.1038/s41598-025-10984-1	Main-text index + Supplementary Table S1
121	IR021	ST045	ST045-I	A2 Other chronic intervention evidence	Rauscher et al. 1997	Music training causes long-term enhancement of preschool children's spatial-temporal reasoning	Neurol. Res.	10.1080/01616412.1997.11740765	Main-text index + Supplementary Table S1
122	IR024	ST048	ST048-X	B2 Cross-sectional 24-hour movement/guideline evidence	Phillips 2024	Associations Between Childcare Physical Activity and Sedentary Time and Early Childhood Developmental Outcomes: A Compositional and Isotemporal Substitution Analysis	Child Care Health Dev.	10.1111/cch.70011	Main-text index + Supplementary Table S1
123	IR029	ST052	ST052-I	A2 Other chronic intervention evidence	Moreno 2011	Short-term music training enhances verbal intelligence and executive function	Psychol. Sci.	10.1177/0956797611416999	Main-text index + Supplementary Table S1
124	IR030	ST053	ST053-L	B1 Longitudinal observational evidence	Gashaj 2021	The effect of video games, exergames and board games on executive functions in kindergarten and 2nd grade: An explorative longitudinal study	Trends Neurosci. Educ.	10.1016/j.tine.2021.100162	Main-text index + Supplementary Table S1
125	IR031	ST054	ST054-L	B1 Longitudinal observational evidence	Padmapriya 2024	The prospective associations of 24-hour movement behaviors and domain-specific activities with executive function and academic achievement among school-aged children in Singapore	Front. Public Health	10.3389/fpubh.2024.1412634	Main-text index + Supplementary Table S1
126	IR032	ST055	ST055-X	B3 Cross-sectional motor competence/fitness evidence	Button 2026	Relationships Among Activity, Motor Skill Performance and Executive Function in Preschool Children: Observational Report	Child Care Health Dev.	10.1111/cch.70263	Main-text index + Supplementary Table S1
127	IR043	ST066	ST066-I	A2 Other chronic intervention evidence	Carson 2022	The impact of new government childcare accreditation standards on children's in-care physical activity and sedentary time	BMC Public Health	10.1186/s12889-022-12888-5	Main-text index + Supplementary Table S1
128	IR044	ST063	ST063-L	B1 Longitudinal observational evidence	Vabø 2025	Multivariate longitudinal associations between 24-hour movement behaviours, self-regulation, executive function and academic skills in 3-5-year-old children from the ACTNOW study	BMC Public Health	10.1186/s12889-025-24605-z	Main-text index + Supplementary Table S1
129	IR049	ST071	ST071-X	B2 Cross-sectional 24-hour movement/guideline evidence	Ltifi 2026	Associations Between Body Mass Index, Movement Behaviors, Motor Skills, Inhibition and Visuospatial Working Memory in Preschool Children: A Cross-Sectional Study Based on WHO References	Children	10.3390/children13020306	Main-text index + Supplementary Table S1
130	IR051	ST073	ST073-X	B4 Cross-sectional activity/sport/play/arts/context evidence	Ghanamah 2025	The Impact of Physical Activity and Screen Time on Motor Creativity in Kindergarteners	Children	10.3390/children12020116	Main-text index + Supplementary Table S1
131	IR052	ST074	ST074-X	B2 Cross-sectional 24-hour movement/guideline evidence	Wang 2026	The Goldilocks day for preschoolers' health outcomes: A compositional data analysis of 24-H movement behaviors on weekdays and weekends	J. Exerc. Sci. Fit.	10.1016/j.jesf.2025.11.004	Main-text index + Supplementary Table S1
132	IR054	ST024	ST024-X	B3 Cross-sectional motor competence/fitness evidence	Zheng 2023	Differential features of early childhood motor skill development and working memory processing: evidence from fNIRS	Front. Behav. Neurosci.	10.3389/fnbeh.2023.1279648	Main-text index + Supplementary Table S1
133	IR066	ST087	ST087-X	B2 Cross-sectional 24-hour movement/guideline evidence	Ltifi 2025	Exploring urban-rural differences in 24-h movement behaviours among Tunisian preschoolers: Insights from the SUNRISE study	Sports Med. Health Sci.	10.1016/j.smhs.2024.03.004	Main-text index + Supplementary Table S1
134	IR068	ST089	ST089-X	B3 Cross-sectional motor competence/fitness evidence	Ma 2024	Relationships between gross motor skills, psychological resilience, executive function, and emotional regulation among Chinese rural preschoolers: A moderated mediation model	Heliyon	10.1016/j.heliyon.2024.e38039	Main-text index + Supplementary Table S1
135	IR069	ST090	ST090-X	B4 Cross-sectional activity/sport/play/arts/context evidence	Chen 2025	Physical activity and executive function in Chinese preschool children: the mediating role of sleep problems	Front. Psychol.	10.3389/fpsyg.2025.1606776	Main-text index + Supplementary Table S1
136	IR070	ST091	ST091-I	A2 Other chronic intervention evidence	Yu 2026	Effects of orienteering exercise on spatial visualization ability in preschool children aged 4-5 years: Behavioral and fNIRS evidence	Dev. Cogn. Neurosci.	10.1016/j.dcn.2026.101725	Main-text index + Supplementary Table S1
137	IR072	ST063	ST063-X	B2 Cross-sectional 24-hour movement/guideline evidence	Vabø 2022	The Multivariate Physical Activity Signatures Associated With Self-Regulation, Executive Function, and Early Academic Learning in 3–5-Year-Old Children	Front. Psychol.	10.3389/fpsyg.2022.842271	Main-text index + Supplementary Table S1
138	IR096	ST050	ST050-X	B2 Cross-sectional 24-hour movement/guideline evidence	McNeill 2018	Physical activity and modified organized sport among preschool children: Associations with cognitive and psychosocial health	Ment. Health Phys. Act.	10.1016/j.mhpa.2018.07.001	Main-text index + Supplementary Table S1
139	IR102	ST121	ST121-X	B3 Cross-sectional motor competence/fitness evidence	Zhilyaeva 2025	Assessment of motor skills associations with executive functioning in children of senior preschool age	Psychol. Sci. Educ.	10.17759/pse.2025300302	Main-text index + Supplementary Table S1
140	IR106	ST125	ST125-X	B4 Cross-sectional activity/sport/play/arts/context evidence	Becker 2014	Physical Activity, Self-Regulation, and Early Academic Achievement in Preschool Children	Early Educ. Dev.	10.1080/10409289.2013.780505	Main-text index + Supplementary Table S1
141	IR107	ST011	ST011-L	B1 Longitudinal observational evidence	Becker 2021	Run fast and sit still: Connections among aerobic fitness, physical activity, and sedentary time with executive function during pre-kindergarten	Early Child. Res. Q.	10.1016/j.ecresq.2021.04.007	Main-text index + Supplementary Table S1
142	IR109	ST013	ST013-L	B1 Longitudinal observational evidence	Zhang 2022	Longitudinal associations of subjectively-measured physical activity and screen time with cognitive development in young children	Ment. Health Phys. Act.	10.1016/j.mhpa.2022.100447	Main-text index + Supplementary Table S1

Ref.	Report ID	Study ID	Unit ID	SWiM cell	Author/year	Article title	Journal	DOI	Citation role
143	IR111	ST015	ST015-X	B2 Cross-sectional 24-hour movement/guideline evidence	McGowan 2023	Meeting 24-hour movement behavior guidelines in young children: Improved quantity estimation and self-regulation	Early Educ. Dev.	10.1080/10409289.2022.2056694	Main-text index + Supplementary Table S1
144	IR113	ST017	ST017-X	B3 Cross-sectional motor competence/fitness evidence	Michel 2022	Fine motor skill automatization and working memory in children with and without potential fine motor impairments: An explorative study	Hum. Mov. Sci.	10.1016/j.humov.2022.102968	Main-text index + Supplementary Table S1
145	IR116	ST020	ST020-L	B1 Longitudinal observational evidence	Colliver 2022	Free play predicts self-regulation years later: Longitudinal evidence from a large Australian sample of toddlers and preschoolers	Early Child. Res. Q.	10.1016/j.ecresq.2021.11.011	Main-text index + Supplementary Table S1
146	IR120	ST024	ST024-X	B3 Cross-sectional motor competence/fitness evidence	Zheng 2024	Differential characteristics of motor development levels inhibitory control and cognitive flexibility processing in preschool children	Chin. J. Sch. Health	10.16835/j.cnki.1000-9817.2024060	Main-text index + Supplementary Table S1
147	IR125	ST029	ST029-X	B4 Cross-sectional activity/sport/play/arts/context evidence	Qu 2020	Study on the association between physical activity and executive function in preschoolers	Chin. J. Child Health Care	10.11852/zgetbjzz2019-1873	Main-text index + Supplementary Table S1
148	IR126	ST030	ST030-X	B4 Cross-sectional activity/sport/play/arts/context evidence	Bain 1978	The cognitive flexibility claim in the bilingual and music education research traditions	J. Res. Music Educ.	10.2307/3344881	Main-text index + Supplementary Table S1
149	IR127	ST031	ST031-X	B3 Cross-sectional motor competence/fitness evidence	Gandotra 2023	An exploratory study of the relationship between motor skills and indicators of cognitive and socio-emotional development in preschoolers	Eur. J. Dev. Psychol.	10.1080/17405629.2022.2028617	Main-text index + Supplementary Table S1
150	IR128	ST032	ST032-I	A2 Other chronic intervention evidence	Caracci 2022	The positive learning transfer from a musical play early-learning system® to young children's linguistic and spatial skills	Music Educ. Res.	10.1080/14613808.2022.2076820	Main-text index + Supplementary Table S1
151	IR130	ST034	ST034-L	B1 Longitudinal observational evidence	Cameron 2012	Fine Motor Skills and Executive Function Both Contribute to Kindergarten Achievement	Child Dev.	10.1111/j.1467-8624.2012.01768.x	Main-text index + Supplementary Table S1

Supplementary Table S2

Full-text reports excluded after eligibility assessment (n = 38)

Early Childhood Movement- and Arts-Based Experiences and Executive Functions: A Systematic Review and Meta-analysis
PROSPERO CRD420261363924

PRISMA category reconciliation

Full-text exclusion category	n
Wrong exposure/outcome or no extractable data	15
Wrong population/age or exposure window	13
Wrong publication type/design	9
Duplicate report	1
Total excluded after full-text assessment	38

Retrieval number corresponds to the source numbering retained in the project screening record. Reports not retrieved or not assessable (n = 13) were retrieval-stage losses and are not included in this full-text exclusion table. The archived project log did not preserve a reliable report-level split within that combined category, so no retrospective subdivision was introduced.

A. Wrong exposure/outcome or no extractable data (n = 15)

No.	Retrieval no.	Report	DOI / identifier	Specific full-text exclusion reason
1	5	Schmithorst, V.J. et al. The effect of musical training on the neural correlates of math processing: a functional magnetic resonance imaging study in humans. Neuroscience Letters. 2004.	10.1016/j.neulet.2003.10.037	The report assessed neural activation during mathematical processing; it did not provide an eligible executive-function or visuospatial performance outcome.
2	10	Delisle Nystrom, C. et al. International Study of Movement Behaviors in the Early Years (SUNRISE): Results from SUNRISE Sweden's Pilot and COVID-19 Study. International Journal of Environmental Research and Public Health. 2020.	10.3390/ijerph17228491	SUNRISE feasibility/COVID-19 movement-behaviour report; no eligible executive-function or visuospatial outcome was reported.

No.	Retrieval no.	Report	DOI / identifier	Specific full-text exclusion reason
3	11	Zarghani, N.H. et al. International study of 24-h movement behaviors of the early years (SUNRISE): A pilot study from Iran. Child: Care, Health and Development. 2024.	10.1111/cch.13269	SUNRISE pilot report; the cognitive outcome data required by the review protocol were absent or not extractable.
4	17	Jordan, T.M. et al. From Tones to Tunes: Emerging Associations Between Tonal Language Immersion Education and Musical Pitch Perception in Children. Developmental Science. 2026.	10.1111/desc.70201	The exposure was tonal-language immersion and the outcome was musical pitch perception; the report did not provide an eligible movement/arts exposure-to-executive-function or visuospatial contrast.
5	24	Tanaka, C. et al. Accelerometer-Measured Physical Activity and Sedentary Time among Children in Japan before and during COVID-19: A Cross-Sectional and Longitudinal Analysis. International Journal of Environmental Research and Public Health. 2023.	10.3390/ijerph20021130	The report described physical activity and sedentary time before/during COVID-19 but did not include an eligible executive-function or visuospatial outcome.
6	81	Escolano-Perez, E. et al. Teacher-Rated Executive Functions, Gender and Relative Age: Independent and Interactive Effects on Observed Fundamental Motor Skills in Kindergarteners. Frontiers in Psychology. 2022.	10.3389/fpsyg.2022.848525	The analytic direction was executive functions to motor skills; it did not evaluate an eligible movement- or arts-based exposure in relation to a cognitive outcome.
7	82	Mule, D. et al. Correlation between Language Development and Motor Skills, Physical Activity, and Leisure Time Behaviour in Preschool-Aged Children. Children. 2022.	10.3390/children9030431	The cognitive outcome was language development; no eligible executive-function or visuospatial outcome was extractable.
8	84	Chang, Z. et al. The role of 24-h movement behaviors in preschool children's learning ability: the mediating effect of inhibitory control. Frontiers in Psychology. 2025.	10.3389/fpsyg.2025.1673960	Inhibitory control was used as a mediator of learning ability, and no directly extractable exposure-to-executive-function estimate met the prespecified analytic framework.
9	87	Brandon, D. et al. Summer Success: SKIPing to Motor Competence for Disadvantaged Preschoolers. Children. 2025.	10.3390/children12050578	The intervention report focused on motor competence and did not provide an eligible executive-function or visuospatial outcome.
10	88	Bazzano, A.N. et al. Yoga and Mindfulness for Social-Emotional Development and Resilience in 3-5 Year-Old Children: Non-Randomized, Controlled Intervention. Psychology Research and Behavior Management. 2023.	10.2147/PRBM.S385413	The outcomes were social-emotional development and resilience; no eligible core executive-function or visuospatial outcome was reported.
11	115	Chichinina, E. et al. Executive function skills predict motor competence in preschool children. BMC Pediatrics. 2025.	10.1186/s12887-025-05582-z	The analytic direction was executive functions to motor competence; no eligible movement/arts exposure-to-executive-function association was available.
12	141	Lundy, A. et al. Effects of Active Outdoor Play on Preschool Children's On-Task Classroom Behavior. Early Childhood Education Journal. 2021.	10.1007/s10643-020-01086-w	The outcome was on-task classroom behaviour rather than a prespecified executive-function or visuospatial measure.
13	149	Martzog, P. et al. Screen media are associated with fine motor skill development in preschool children. Early Childhood Research Quarterly. 2022.	10.1016/j.ecresq.2022.03.010	The exposure was screen media and the outcome was fine-motor development; the report did not contain an eligible exposure-cognitive-outcome pair.
14	151	Rodriguez, A.N. et al. Educational play-based movement integration program in Early Childhood Education: evaluation and analysis. SPORTIS. 2020.	10.17979/sportis.2020.6.3.6158	The programme evaluation did not report an extractable prespecified executive-function or visuospatial outcome.
15	175	Drake, J.E. Two Kinds of Visual Art Prodigies: Characteristics and Future Trajectories. Creativity Research Journal. 2025.	Not reported in retrieval list	The report described characteristics and trajectories of visual-art prodigies but did not provide an eligible executive-function or visuospatial outcome linked to a prespecified arts exposure.

B. Wrong population/age or exposure window (n = 13)

No.	Retrieval no.	Report	DOI / identifier	Specific full-text exclusion reason
16	4	Yaffe, D. et al. Short playful interactions improve executive functions in children. Scientific Reports. 2025.	10.1038/s41598-025-07028-z	Participants were 6-10 years old; the exposure was not confined to the prespecified birth-to-6-year window, and no eligible subgroup was extractable.
17	13	Chang, Y.-K. et al. The impacts of coordinative exercise on executive function in kindergarten children: An ERP study. Experimental Brain Research. 2013.	10.1007/s00221-012-3360-9	Participants were 6-7.5 years old (mean approximately 7 years); the intervention was delivered outside the prespecified <=6-year exposure window.
18	27	Oberer, N. et al. Motor skills in kindergarten: Internal structure, cognitive correlates and relationships to background variables. Human Movement Science. 2017.	10.1016/j.humov.2017.02.002	The kindergarten sample had a mean age above 6 years (approximately 6.45 years), with no separately extractable <=6-year subgroup.
19	46	Mierau, A. et al. Acute exercise induces cortical inhibition and reduces arousal in response to visual stimulation in young children. International Journal of Developmental Neuroscience. 2014.	10.1016/j.ijdevneu.2013.12.009	The analysed child sample extended beyond the protocol's <=6-year window, and no separable eligible early-childhood subgroup was reported.
20	47	Vandenbroucke, L. et al. Longitudinal Associations Between Objectively Measured Physical Activity and Development of Executive Functioning Across the Transition to First Grade. Journal of Physical Activity & Health. 2016.	10.1123/jpah.2015-0708	Physical activity exposure was measured in the middle of first grade, after the eligible early-childhood exposure window.
21	53	Byman, A. et al. Associations of executive functions with physical activity and sedentary time in children aged 5-7 years: a multi-methodological pilot study. Child Neuropsychology. 2026.	10.1080/09297049.2025.2509911	Mixed 5-7-year-old sample with no separately extractable subgroup meeting the <=6-year criterion.

No.	Retrieval no.	Report	DOI / identifier	Specific full-text exclusion reason
22	83	Bulten, R. et al. Effect of cognitively engaging physical activity on executive functions in children. Frontiers in Psychology. 2022.	10.3389/fpsyg.2022.841192	The acute intervention was conducted in school-aged children outside the prespecified <=6-year exposure window.
23	93	Fisher, A. et al. Effects of a physical education intervention on cognitive function in young children: randomized controlled pilot study. BMC Pediatrics. 2011.	10.1186/1471-2431-11-97	The primary-school sample had a mean age of 6.2 years; the intervention therefore fell outside the protocol's <=6-year threshold.
24	102	Chen, J.J. et al. The relationship between early musical training and executive functions: Validation of effects of the sensitive period. Psychology of Music. 2022.	10.1177/030573562097 8690	The school-aged sample used a broader early-training/sensitive-period definition and did not provide a separable exposure cohort restricted to <=6 years/72 months.
25	112	van Stryp, O. et al. The effect of active brain-breaks on fundamental movement skills and executive functioning of Grade one children in Cape Town, South Africa. Early Child Development and Care. 2024.	10.1080/03004430.2023.2282360	The intervention was delivered to Grade-one children, after the eligible early-childhood exposure window.
26	121	Joret, M.-E. et al. Cognitive inhibitory control in children following early childhood music education. Musicae Scientiae. 2017.	10.1177/1029864916655477	Participants were 9-12 years old; the analysed music-training comparison was outside the prespecified early-childhood exposure window.
27	139	Becker, D.R. et al. Open-Skilled Sport, Sport Intensity, Executive Function, and Academic Achievement in Grade School Children. Early Education and Development. 2018.	10.1080/10409289.2018.1479079	The study involved grade-school children and current sport exposure outside the <=6-year window.
28	148	Vasilopoulos, F. et al. Investigation of the associations between physical activity, self-regulation and educational outcomes in childhood. PLOS ONE. 2021.	10.1371/journal.pone.0250984	The secondary cohort analyses assessed children at ages 7, 11 and 14 years; no eligible <=6-year exposure subgroup was available.

C. Wrong publication type/design (n = 9)

No.	Retrieval no.	Report	DOI / identifier	Specific full-text exclusion reason
29	44	Rudd, J.R. et al. Physical Education Pedagogies Built upon Theories of Movement Learning: How Can Environmental Constraints Be Manipulated to Improve Children's Executive Function and Self-Regulation Skills? International Journal of Environmental Research and Public Health. 2019.	10.3390/ijerph16091630	Conceptual/pedagogical article; it did not report original participant-level exposure-outcome data.
30	56	Trainor, L.J. Understanding the benefits of musical training: effects on oscillatory brain activity. Annals of the New York Academy of Sciences. 2009.	10.1111/j.1749-6632.2009.04589.x	Narrative/theoretical review rather than a primary empirical study.
31	113	Yetti, E. et al. The Influence of the Dance Creativity on Executive Functions of Early Childhood. Proceedings of the International Conference on Arts and Design Education (ICADE 2018). 2019.	10.2991/icade-18.2019.59	Conference-proceedings paper; excluded under the final published-journal-article criterion.
32	127	Mossmann, J.B. et al. Evaluation of the Usability and Playability of an Exergame for Executive Functions Stimulation and Its Development Process. Engineering Psychology and Cognitive Ergonomics: Performance, Emotion and Situation Awareness, EPCE 2017. 2017.	10.1007/978-3-319-58472-0_14	Conference book chapter focused on usability/playability and development; not an eligible published journal primary study.
33	146	McGowan, A.L. et al. Infusing Physical Activity into Early Childhood Classrooms: Guidance for Best Practices. Early Childhood Education Journal. 2024.	10.1007/s10643-023-01532-5	Best-practice/guidance article; no original eligible participant study was reported.
34	158	Campollo-Urkiza, A. Development of a Programme of Musical Activities for the Contribution of Unplugged Computational Thinking in Early Childhood Education. Revista Electronica Educare. 2023.	10.15359/ree.27-3.17180	Qualitative didactic programme-development report; the design did not provide an analytically comparable eligible exposure-outcome study for this review.
35	159	Perea, A. et al. Analysis of behaviour in sports through polar coordinate analysis with MATLAB. Quality & Quantity. 2012.	10.1007/s11135-011-9435-z	Methodological research note on polar-coordinate analysis in sport; not a child exposure-outcome study.
36	168	Suhartini, B.; Kushartanti, B.M.W. Development of Physical Activity Models Based on Motor Perception for Kindergarten Children. International Journal of Human Movement and Sports Sciences. 2022.	10.13189/saj.2022.100102	Model-development study rather than an eligible executive-function/visuospatial exposure-outcome design.
37	181	McGrath, K. Effects of Cognitively Engaging Physical Activity on Preschool Children's Cognitive Outcomes. JOPERD: The Journal of Physical Education, Recreation & Dance. 2024.	No DOI reported in retrieval list	Research-into-practice/secondary summary of another study; not an original primary report.

D. Duplicate report (n = 1)

No.	Retrieval no.	Report	DOI / identifier	Specific full-text exclusion reason
38	165	Zhang, J. et al. Effects of moderate-intensity gymnastics on executive function in children aged 5 to 6 years. Chinese Journal of School Health. 2024.	10.16835/j.cnki.1000-9817.2024082	Same or overlapping moderate-intensity gymnastics trial as the retained PsyCh Journal report (Zhang et al., 2024; DOI 10.1002/pchj.786); counted only once after report-level consolidation.

Notes

- The unit of reporting is the report, not the unique study.
- Only one principal exclusion reason is shown per report, in accordance with PRISMA reporting practice.
- The category totals reproduce the frozen project flow exactly: 15 + 13 + 9 + 1 = 38.
- Reason wording was standardized to align with the registered eligibility criteria: exposure initiated, delivered or measured at age 6 years or younger; eligible movement-, physical activity-, motor-, arts-, music-, dance-, drama- or mixed movement–arts exposure; and an eligible executive-function or visuospatial outcome.

Supplementary Table S3. Study-level consolidation and synthesis allocation

The 130 included reports were consolidated into 125 Study IDs. This table preserves report- and unit-level traceability while applying the final analytical hierarchy. No harmonised country or sample-size field was inferred when it was not preserved in the submitted report catalogue.

Study ID	Final ref(s)	Author/year	Report ID(s)	Unit ID(s)	SWiM cell(s)	Reports	Final synthesis allocation	Key note
ST001	61	Koepp 2022	IR001	ST001-I	A3	1	Acute independent/quasi-experimental evidence — structured narrative synthesis	
ST002	116	Linnavalli 2018	IR002	ST002-I	A2	1	Other chronic intervention evidence — structured narrative synthesis	
ST003	45	Giordano 2022	IR003	ST003-I	A2	1	Other chronic intervention evidence — structured narrative synthesis	
ST004	77	Lau 2024	IR004	ST004-X	B2	1	Cross-sectional 24-hour movement/guideline evidence — structured narrative synthesis	
ST005	27	Zhang 2024	IR005	ST005-P	A1	1	Core quantitative intervention — Inhibition; Working memory; Cognitive flexibility	Core inhibition/working-memory/flexibility effects retained; hot-EF delay-gratification tasks reported narratively.
ST006	110	Strait 2012	IR006	ST006-X	B4	1	Cross-sectional activity/sport/play/arts/context evidence — structured narrative synthesis	
ST007	66	Taylor 2021	IR007	ST007-L	B1	1	Longitudinal observational evidence — structured narrative synthesis	
ST008	117	Yang 2024	IR008	ST008-L	B1	1	Longitudinal observational evidence — structured narrative synthesis	
ST009	118	Zhang 2022	IR009	ST009-I	A2	1	Other chronic intervention evidence — structured narrative synthesis	
ST010	83	Abdeta 2025	IR010	ST010-X	B2	1	Cross-sectional 24-hour movement/guideline evidence — structured narrative synthesis	
ST011	141	Becker 2021	IR107	ST011-L	B1	1	Longitudinal observational evidence — structured narrative synthesis	
ST012	11	Biino 2023	IR108	ST012-P	A1	1	Eligible chronic-intervention candidate; non-model-ready; excluded from meta-analysis and retained in the evidence map.	
ST013	142	Zhang 2022	IR109	ST013-L	B1	1	Longitudinal observational evidence — structured narrative synthesis	
ST014	67	Howard 2018	IR110	ST014-L	B1	1	Longitudinal observational evidence — structured narrative synthesis	
ST015	143	McGowan 2023	IR111	ST015-X	B2	1	Cross-sectional 24-hour movement/guideline evidence — structured narrative synthesis	
ST016	108; 109	Cook 2019; Cook 2021	IR025; IR112	ST016-X	B4	2	Cross-sectional activity/sport/play/arts/context evidence — structured narrative synthesis	Companion reports consolidated at Study ID level.
ST017	144	Michel 2022	IR113	ST017-X	B3	1	Cross-sectional motor-competence/fitness evidence — structured narrative synthesis	
ST018	46	Capio 2024	IR114	ST018-I	A2	1	Other chronic intervention evidence — structured narrative synthesis	
ST019	85	Carson 2017	IR115	ST019-X	B2	1	Cross-sectional 24-hour movement/guideline evidence — structured narrative synthesis	
ST020	145	Colliver 2022	IR116	ST020-L	B1	1	Longitudinal observational evidence — structured narrative synthesis	
ST021	69	Verswijveren 2020	IR117	ST021-L	B1	1	Longitudinal observational evidence — structured narrative synthesis	

Study ID	Final ref(s)	Author/year	Report ID(s)	Unit ID(s)	SWiM cell(s)	Reports	Final synthesis allocation	Key note
ST022	53	García Núñez 2024	IR118	ST022-I	A2	1	Other chronic intervention evidence — structured narrative synthesis	
ST023	90	Zhang 2024	IR119	ST023-X	B3	1	Cross-sectional motor-competence/fitness evidence — structured narrative synthesis	
ST024	132; 146	Zheng 2023; Zheng 2024	IR054; IR120	ST024-X	B3	2	Cross-sectional motor-competence/fitness evidence — structured narrative synthesis	Companion reports consolidated at Study ID level.
ST025	54	Chaiakaraphong 2021	IR121	ST025-I	A2	1	Other chronic intervention evidence — structured narrative synthesis	
ST026	38	Ilari 2021	IR122	ST026-P	A1	1	Core quantitative intervention — Working memory; Cognitive flexibility	
ST027	86	Tanaka 2020	IR123	ST027-X	B2	1	Cross-sectional 24-hour movement/guideline evidence — structured narrative synthesis	
ST028	111	Dolgikh 2022	IR124	ST028-X	B4	1	Cross-sectional activity/sport/play/arts/context evidence — structured narrative synthesis	
ST029	147	Qu 2020	IR125	ST029-X	B4	1	Cross-sectional activity/sport/play/arts/context evidence — structured narrative synthesis	
ST030	148	Bain 1978	IR126	ST030-X	B4	1	Cross-sectional activity/sport/play/arts/context evidence — structured narrative synthesis	
ST031	149	Gandotra 2023	IR127	ST031-X	B3	1	Cross-sectional motor-competence/fitness evidence — structured narrative synthesis	
ST032	150	Caracci 2022	IR128	ST032-I	A2	1	Other chronic intervention evidence — structured narrative synthesis	
ST033	104	Slot 2017	IR129	ST033-X	B4	1	Cross-sectional activity/sport/play/arts/context evidence — structured narrative synthesis	
ST034	151	Cameron 2012	IR130	ST034-L	B1	1	Longitudinal observational evidence — structured narrative synthesis	
ST035	50	Mavilidi et al. 2023	IR011	ST035-I	A2	1	Other chronic intervention evidence — structured narrative synthesis	
ST036	80	Batista Albuquerque Goulart Lemos et al. 2026	IR012	ST036-X	B2	1	Cross-sectional 24-hour movement/guideline evidence — structured narrative synthesis	
ST037	97	Fernandes et al. 2022	IR013	ST037-X	B3	1	Cross-sectional motor-competence/fitness evidence — structured narrative synthesis	
ST038	119	Kosokabe et al. 2021	IR014	ST038-I	A2	1	Other chronic intervention evidence — structured narrative synthesis	
ST039	51	Wen et al. 2018	IR015	ST039-I	A2	1	Other chronic intervention evidence — structured narrative synthesis	
ST040	47	Li et al. 2025	IR016	ST040-I	A2	1	Other chronic intervention evidence — structured narrative synthesis	
ST041	78	Bezerra et al. 2021	IR017	ST041-X	B2	1	Cross-sectional 24-hour movement/guideline evidence — structured narrative synthesis	
ST042	120	Kosokabe et al. 2025	IR018	ST042-I	A2	1	Other chronic intervention evidence — structured narrative synthesis	
ST043	48	Olive et al. 2024	IR019	ST043-I	A2	1	Other chronic intervention evidence — structured narrative synthesis	
ST044	81	Kuzik et al. 2020	IR020	ST044-X	B2	1	Cross-sectional 24-hour movement/guideline evidence — structured narrative synthesis	
ST045	121	Rauscher et al. 1997	IR021	ST045-I	A2	1	Other chronic intervention evidence — structured narrative synthesis	
ST046	57	Ureña et al. 2020	IR022	ST046-I	A3	1	Acute independent/quasi-experimental evidence — structured narrative synthesis	
ST047	14	Bentley 2023	IR023	ST047-I	A2	1	Other chronic intervention evidence — structured narrative synthesis	
ST048	122	Phillips 2024	IR024	ST048-X	B2	1	Cross-sectional 24-hour movement/guideline evidence — structured narrative synthesis	
ST049	49	Mulvey 2018	IR026	ST049-I	A2	1	Other chronic intervention evidence — structured narrative synthesis	
ST050	70; 138	McNeill 2020; McNeill 2018	IR027; IR096	ST050-L; ST050-X	B1; B2	2	Longitudinal observational evidence — structured narrative synthesis; Cross-sectional 24-hour movement/guideline evidence — structured narrative synthesis	Companion reports consolidated at Study ID level.

Study ID	Final ref(s)	Author/year	Report ID(s)	Unit ID(s)	SWiM cell(s)	Reports	Final synthesis allocation	Key note
ST051	82	Zahran 2024	IR028	ST051-X	B2	1	Cross-sectional 24-hour movement/guideline evidence — structured narrative synthesis	
ST052	123	Moreno 2011	IR029	ST052-I	A2	1	Other chronic intervention evidence — structured narrative synthesis	
ST053	124	Gashaj 2021	IR030	ST053-L	B1	1	Longitudinal observational evidence — structured narrative synthesis	
ST054	125	Padmapriya 2024	IR031	ST054-L	B1	1	Longitudinal observational evidence — structured narrative synthesis	
ST055	126	Button 2026	IR032	ST055-X	B3	1	Cross-sectional motor-competence/fitness evidence — structured narrative synthesis	
ST056	79	Chang 2025	IR033	ST056-X	B2	1	Cross-sectional 24-hour movement/guideline evidence — structured narrative synthesis	
ST057	28	Başarır 2025	IR034	ST057-P	A1	1	Core quantitative intervention — Inhibition	
ST058	71	López-Vicente 2017	IR035	ST058-L	B1	1	Longitudinal observational evidence — structured narrative synthesis	
ST059	72	Becker 2025	IR036	ST059-L	B1	1	Longitudinal observational evidence — structured narrative synthesis	
ST060	29	He 2025	IR037	ST060-P	A1	1	Eligible chronic-intervention candidate; non-model-ready; excluded from meta-analysis and retained in the evidence map.	
ST061	30	Xiong 2017	IR038	ST061-P	A1	1	Core quantitative intervention — Cognitive flexibility	
ST062	98	Starrett 2026	IR039	ST062-X	B3	1	Cross-sectional motor-competence/fitness evidence — structured narrative synthesis	
ST063	52; 128; 137	Buene Vabø 2025; Vabø 2025; Vabø 2022	IR040; IR044; IR072	ST063-I; ST063-L; ST063-X	A2; B1; B2	3	Other chronic intervention evidence — structured narrative synthesis; Longitudinal observational evidence — structured narrative synthesis; Cross-sectional 24-hour movement/guideline evidence — structured narrative synthesis	Companion reports consolidated at Study ID level.
ST064	73	Li 2026	IR041	ST064-L	B1	1	Longitudinal observational evidence — structured narrative synthesis	
ST065	84	Byambaa 2026	IR042	ST065-X	B2	1	Cross-sectional 24-hour movement/guideline evidence — structured narrative synthesis	
ST066	127	Carson 2022	IR043	ST066-I	A2	1	Other chronic intervention evidence — structured narrative synthesis	
ST067	87	Draper 2020	IR045	ST067-X	B2	1	Cross-sectional 24-hour movement/guideline evidence — structured narrative synthesis	
ST068	55	Brian 2026	IR046	ST068-I	A2	1	Other chronic intervention evidence — structured narrative synthesis	
ST069	91	Han 2022	IR047	ST069-X	B3	1	Cross-sectional motor-competence/fitness evidence — structured narrative synthesis	
ST070	36	Chiu 2026	IR048	ST070-P	A1	1	Core quantitative intervention — Working memory	
ST071	129	Ltifi 2026	IR049	ST071-X	B2	1	Cross-sectional 24-hour movement/guideline evidence — structured narrative synthesis	
ST072	13	Shen 2019	IR050	ST072-P	A1	1	Core quantitative intervention — Inhibition; Working memory; Cognitive flexibility	Core inhibition/working-memory/flexibility effects retained; Dot Matrix treated as a working-memory task-selection sensitivity.
ST073	130	Ghanamah 2025	IR051	ST073-X	B4	1	Cross-sectional activity/sport/play/arts/context evidence — structured narrative synthesis	
ST074	131	Wang 2026	IR052	ST074-X	B2	1	Cross-sectional 24-hour movement/guideline evidence — structured narrative synthesis	
ST075	114	Lucendo-Noriega 2026	IR053	ST075-X	B4	1	Cross-sectional activity/sport/play/arts/context evidence — structured narrative synthesis	
ST076	31	Ltifi 2025	IR055	ST076-P	A1	1	Core quantitative intervention — Inhibition; Working memory	
ST077	88	Munambah 2021	IR056	ST077-X	B2	1	Cross-sectional 24-hour movement/guideline evidence — structured narrative synthesis	
ST078	106	Aguayo 2021	IR057	ST078-X	B4	1	Cross-sectional activity/sport/play/arts/context evidence — structured narrative synthesis	
ST079	40	Wu 2026	IR058	ST079-P	A1	1	Core quantitative intervention — Inhibition; Working memory; Cognitive flexibility	

Study ID	Final ref(s)	Author/year	Report ID(s)	Unit ID(s)	SWiM cell(s)	Reports	Final synthesis allocation	Key note
ST080	105	Wang 2025	IR059	ST080-X	B4	1	Cross-sectional activity/sport/play/arts/context evidence — structured narrative synthesis	
ST081	39	Shen 2020	IR060	ST081-P	A1	1	Core quantitative intervention — Inhibition; Working memory; Cognitive flexibility	
ST082	32	Bai 2022	IR061	ST082-P	A1	1	Core quantitative intervention — Inhibition; Working memory; Cognitive flexibility	Core component effects retained; deterministic global EF composite excluded to prevent double counting.
ST083	58	Du 2025	IR062	ST083-I	A3	1	Acute independent/quasi-experimental evidence — structured narrative synthesis	
ST084	112	Yakushina 2025	IR063	ST084-X	B4	1	Cross-sectional activity/sport/play/arts/context evidence — structured narrative synthesis	
ST085	62	Fitamen 2021	IR064	ST085-I	A4	1	Acute paired/crossover evidence — structured narrative synthesis	
ST086	92	Luo 2023	IR065	ST086-X	B3	1	Cross-sectional motor-competence/fitness evidence — structured narrative synthesis	
ST087	133	Ltifi 2025	IR066	ST087-X	B2	1	Cross-sectional 24-hour movement/guideline evidence — structured narrative synthesis	
ST088	74	Tandon 2020	IR067	ST088-L	B1	1	Longitudinal observational evidence — structured narrative synthesis	
ST089	134	Ma 2024	IR068	ST089-X	B3	1	Cross-sectional motor-competence/fitness evidence — structured narrative synthesis	
ST090	135	Chen 2025	IR069	ST090-X	B4	1	Cross-sectional activity/sport/play/arts/context evidence — structured narrative synthesis	
ST091	136	Yu 2026	IR070	ST091-I	A2	1	Other chronic intervention evidence — structured narrative synthesis	
ST092	33	Xiong 2019	IR071	ST092-P	A1	1	Core quantitative intervention — Cognitive flexibility	
ST093	93	Liu 2022	IR073	ST093-X	B3	1	Cross-sectional motor-competence/fitness evidence — structured narrative synthesis	
ST094	89	Li 2025	IR074	ST094-X	B2	1	Cross-sectional 24-hour movement/guideline evidence — structured narrative synthesis	
ST095	99	Chichinina 2025	IR075	ST095-X	B3	1	Cross-sectional motor-competence/fitness evidence — structured narrative synthesis	
ST096	35	Vazou 2021	IR076	ST096-P	A1	1	Core quantitative intervention — Inhibition	Core inhibition effect retained; teacher-rated behavioural control reported using the source-preferred adjusted model.
ST097	37	Díaz-Quesada 2025	IR077	ST097-P	A1	1	Eligible chronic-intervention candidate; non-model-ready; excluded from meta-analysis and retained in the evidence map.	
ST098	41	Çiftçi 2022	IR078	ST098-P	A1	1	Eligible chronic-intervention candidate; non-model-ready; excluded from meta-analysis and retained in the evidence map.	
ST099	16	Bayanova 2022	IR079	ST099-X	B4	1	Cross-sectional activity/sport/play/arts/context evidence — structured narrative synthesis	
ST100	15	Suppalarkbunlue 2023	IR084	ST100-P	A1	1	Core quantitative intervention — Inhibition; Working memory; Cognitive flexibility	
ST101	59	Antrilli 2018	IR080	ST101-I	A3	1	Acute independent/quasi-experimental evidence — structured narrative synthesis	
ST102	100	J. Liu 2026	IR081	ST102-X	B3	1	Cross-sectional motor-competence/fitness evidence — structured narrative synthesis	
ST103	75	C. Liu 2026	IR082	ST103-L	B1	1	Longitudinal observational evidence — structured narrative synthesis	
ST104	113	Chichinina 2022	IR083	ST104-X	B4	1	Cross-sectional activity/sport/play/arts/context evidence — structured narrative synthesis	
ST105	107	Bryant 2021	IR085	ST105-X	B4	1	Cross-sectional activity/sport/play/arts/context evidence — structured narrative synthesis	
ST106	76	Chou 2022	IR086	ST106-L	B1	1	Longitudinal observational evidence — structured narrative synthesis	
ST107	42	Hsiao 2025	IR087	ST107-P	A1	1	Core quantitative intervention — Working memory	
ST108	115	Willoughby 2018	IR088	ST108-X	B4	1	Cross-sectional activity/sport/play/arts/context evidence — structured narrative synthesis	

Study ID	Final ref(s)	Author/year	Report ID(s)	Unit ID(s)	SWiM cell(s)	Reports	Final synthesis allocation	Key note
ST109	34	Miller 2023	IR089	ST109-P	A1	1	Secondary intervention evidence — narrative only.	Behavioural self-regulation reported narratively using the source-preferred hurdle-model OR; historical WM/CF effects excluded.
ST110	68	Bryant 2024	IR090	ST110-L	B1	1	Longitudinal observational evidence — structured narrative synthesis	
ST111	102	Capio 2023	IR091	ST111-X	B3	1	Cross-sectional motor-competence/fitness evidence — structured narrative synthesis	
ST112	94	Kong 2025	IR092	ST112-X	B3	1	Cross-sectional motor-competence/fitness evidence — structured narrative synthesis	
ST113	43	Bugos 2017	IR093	ST113-P	A1	1	Eligible chronic-intervention candidate; non-model-ready; excluded from meta-analysis and retained in the evidence map.	
ST114	44	Degé 2022	IR094	ST114-P	A1	1	Core quantitative intervention — Inhibition	
ST115	101	Li 2025	IR095	ST115-X	B3	1	Cross-sectional motor-competence/fitness evidence — structured narrative synthesis	
ST116	60	Preston 2025	IR097	ST116-I	A3	1	Acute independent/quasi-experimental evidence — structured narrative synthesis	
ST117	63	Rosiek 2022	IR098	ST117-I	A4	1	Acute paired/crossover evidence — structured narrative synthesis	
ST118	56	Zach 2015	IR099	ST118-I	A2	1	Other chronic intervention evidence — structured narrative synthesis	
ST119	96	Maurer 2019	IR100	ST119-X	B3	1	Cross-sectional motor-competence/fitness evidence — structured narrative synthesis	
ST120	95	Veldman 2023	IR101	ST120-X	B3	1	Cross-sectional motor-competence/fitness evidence — structured narrative synthesis	
ST121	139	Zhilyaeva 2025	IR102	ST121-X	B3	1	Cross-sectional motor-competence/fitness evidence — structured narrative synthesis	
ST122	64	Tandon 2018	IR103	ST122-I	A4	1	Acute paired/crossover evidence — structured narrative synthesis	
ST123	65	Zhang 2020	IR104	ST123-I	A4	1	Acute paired/crossover evidence — structured narrative synthesis	
ST124	103	Zhang 2018	IR105	ST124-X	B3	1	Cross-sectional motor-competence/fitness evidence — structured narrative synthesis	
ST125	140	Becker 2014	IR106	ST125-X	B4	1	Cross-sectional activity/sport/play/arts/context evidence — structured narrative synthesis	

Supplementary Table S4. Risk of bias for the 15 core quantitative Study IDs

RoB 2 or ROBINS-I was applied according to study design. The table reports the final study-level overall judgment used for the core models. Studies judged Serious remained in the primary analysis and were excluded in the prespecified risk-of-bias sensitivity analysis.

Study ID	Ref.	Author/year	Core domain(s)	Overall judgment	Primary model	Sensitivity action
ST005	27	Zhang 2024	Inhibition; Working memory; Cognitive flexibility	Some concerns	Included in primary model	Retained in sensitivity
ST057	28	Başarır 2025	Inhibition	Some concerns	Included in primary model	Retained in sensitivity
ST076	31	Ltifi 2025	Inhibition; Working memory	Some concerns	Included in primary model	Retained in sensitivity
ST082	32	Bai 2022	Inhibition; Working memory; Cognitive flexibility	Some concerns	Included in primary model	Retained in sensitivity
ST092	33	Xiong 2019	Cognitive flexibility	Some concerns	Included in primary model	Retained in sensitivity
ST096	35	Vazou 2021	Inhibition	Some concerns	Included in primary model	Retained in sensitivity
ST114	44	Degé 2022	Inhibition	Some concerns	Included in primary model	Retained in sensitivity
ST026	38	Ilari 2021	Working memory; Cognitive flexibility	Moderate	Included in primary model	Retained in sensitivity
ST072	13	Shen 2019	Inhibition; Working memory; Cognitive flexibility	Moderate	Included in primary model	Retained in sensitivity
ST081	39	Shen 2020	Inhibition; Working memory; Cognitive flexibility	Moderate	Included in primary model	Retained in sensitivity
ST100	15	Suppalarkbunlue 2023	Inhibition; Working memory; Cognitive flexibility	Moderate	Included in primary model	Retained in sensitivity

Study ID	Ref.	Author/year	Core domain(s)	Overall judgment	Primary model	Sensitivity action
ST107	42	Hsiao 2025	Working memory	Moderate	Included in primary model	Retained in sensitivity
ST061	30	Xiong 2017	Cognitive flexibility	Serious	Included in primary model	Excluded from Serious-RoB sensitivity
ST070	36	Chiu 2026	Working memory	Serious	Included in primary model	Excluded from Serious-RoB sensitivity
ST079	40	Wu 2026	Inhibition; Working memory; Cognitive flexibility	Serious	Included in primary model	Excluded from Serious-RoB sensitivity

Supplementary Table S5. Final evidence-role adjudication for candidate intervention studies and secondary outcomes

This table supersedes the historical 21-study/49-effect quantitative-core classification. The final core contains 15 unique Study IDs and 29 effects. Five candidate projects were excluded because a unique model-ready effect and variance could not be verified without assumptions; one project was retained only as secondary narrative evidence.

Study ID	Ref.	Author/year	Final status	Core domain(s)	Final secondary/evidence role	Rationale	Quantitative action
ST012	11	Biino 2023	Hard-gate exclusion from meta-analysis	None	Evidence map / SWiM traceability only	A unique model-ready effect and variance could not be verified without assumption-based reconstruction.	Exclude from all core models.
ST072	13	Shen 2019	Core quantitative + adjudicated secondary role	Inhibition; Working memory; Cognitive flexibility	Describe as visuospatial working memory; optional substitution sensitivity leaves the working-memory inference unchanged.	Visuospatial working memory; Yes — alternative task within the same study and domain.	Remove the separate visuospatial outcome and retain the task only as a working-memory selection sensitivity.
ST100	15	Suppalarkbunlue 2023	Core quantitative intervention	Inhibition; Working memory; Cognitive flexibility	No separate secondary outcome retained.	One verified effect per prespecified domain and immediate-post timepoint.	Retain core domain effect(s).
ST005	27	Zhang 2024	Core quantitative + adjudicated secondary role	Inhibition; Working memory; Cognitive flexibility	Report mixed delay-gratification findings; do not quote g=0.66 as a global effect.	Hot executive function / delay gratification; Yes — one favourable task had been selected from multiple hot-EF tasks.	Retire the single favourable row and the global/self-regulation pool.
ST057	28	Başarır 2025	Core quantitative intervention	Inhibition	No separate secondary outcome retained.	One verified effect per prespecified domain and immediate-post timepoint.	Retain core domain effect(s).
ST060	29	He 2025	Hard-gate exclusion from meta-analysis	None	Evidence map / SWiM traceability only	A unique model-ready effect and variance could not be verified without assumption-based reconstruction.	Exclude from all core models.
ST061	30	Xiong 2017	Core quantitative intervention	Cognitive flexibility	No separate secondary outcome retained.	One verified effect per prespecified domain and immediate-post timepoint.	Retain core domain effect(s).
ST076	31	Ltifi 2025	Core quantitative intervention	Inhibition; Working memory	No separate secondary outcome retained.	One verified effect per prespecified domain and immediate-post timepoint.	Retain core domain effect(s).
ST082	32	Bai 2022	Core quantitative + adjudicated secondary role	Inhibition; Working memory; Cognitive flexibility	Do not report as a separate outcome; retain the component effects only.	Overall task-based EF composite; Yes — double-counts evidence already represented in the core.	Remove from every secondary model and from the evidence-count numerator.
ST092	33	Xiong 2019	Core quantitative intervention	Cognitive flexibility	No separate secondary outcome retained.	One verified effect per prespecified domain and immediate-post timepoint.	Retain core domain effect(s).
ST109	34	Miller 2023	Secondary narrative only	None	Behavioural self-regulation: OR 2.98 [1.53, 5.81]; cognitive self-regulation outcomes null.	Source-preferred zero-inflated hurdle model is not commensurate with SMD pooling.	Retire historical raw SMD; no core contribution.
ST096	35	Vazou 2021	Core quantitative + adjudicated secondary role	Inhibition	Report the adjusted null/inconclusive behavioural-control result.	Teacher-rated behavioural control; Historical CI is not reproduced by the full-sample raw post-test means.	Retire the historical SMD from pooling.
ST070	36	Chiu 2026	Core quantitative intervention	Working memory	No separate secondary outcome retained.	One verified effect per prespecified domain and immediate-post timepoint.	Retain core domain effect(s).
ST097	37	Díaz-Quesada 2025	Hard-gate exclusion from meta-analysis	None	Evidence map / SWiM traceability only	A unique model-ready effect and variance could not be verified without assumption-based reconstruction.	Exclude from all core models.
ST026	38	Ilari 2021	Core quantitative intervention	Working memory; Cognitive flexibility	No separate secondary outcome retained.	One verified effect per prespecified domain and immediate-post timepoint.	Retain core domain effect(s).
ST081	39	Shen 2020	Core quantitative intervention	Inhibition; Working memory; Cognitive flexibility	No separate secondary outcome retained.	One verified effect per prespecified domain and immediate-post timepoint.	Retain core domain effect(s).
ST079	40	Wu 2026	Core quantitative intervention	Inhibition; Working memory; Cognitive flexibility	No separate secondary outcome retained.	One verified effect per prespecified domain and immediate-post timepoint.	Retain core domain effect(s).
ST098	41	Çiftçi 2022	Hard-gate exclusion from meta-analysis	None	Evidence map / SWiM traceability only	A unique model-ready effect and variance could not be verified without assumption-based reconstruction.	Exclude from all core models.
ST107	42	Hsiao 2025	Core quantitative intervention	Working memory	No separate secondary outcome retained.	One verified effect per prespecified domain and immediate-post timepoint.	Retain core domain effect(s).
ST113	43	Bugos 2017	Hard-gate exclusion from meta-analysis	None	Evidence map / SWiM traceability only	A unique model-ready effect and variance could not be verified without assumption-based reconstruction.	Exclude from all core models.

Study ID	Ref.	Author/year	Final status	Core domain(s)	Final secondary/evidence role	Rationale	Quantitative action
ST114	44	Degé 2022	Core quantitative intervention	Inhibition	No separate secondary outcome retained.	One verified effect per prespecified domain and immediate-post timepoint.	Retain core domain effect(s).

Supplementary Table S6. Frozen model-ready 29-effect executive-function dataset

One immediate-post effect was retained per Study ID and core executive-function domain. Positive Hedges g values favour the intervention. Participants recur across domains; domain totals must not be summed to obtain a unique overall participant count.

Effect ID	Study/ref.	Author/year	Domain	Selected outcome	Timepoint	nI/nC	g	95% CI	RoB	Weight
ST005_I	ST005 [27]	Zhang 2024	Inhibition	Stop Signal Task accuracy	Immediate post (12 weeks)	31/32	-0.382	-0.881 to 0.116	Some concerns	10.4%
ST057_I	ST057 [28]	Başarır 2025	Inhibition	Go/No-Go response time	Immediate post (8 weeks)	26/25	0.529	-0.030 to 1.088	Some concerns	9.8%
ST072_I	ST072 [13]	Shen 2019	Inhibition	Day/Night Stroop score	Immediate post (T2)	30/31	1.311	0.756 to 1.866	Moderate	9.8%
ST076_I	ST076 [31]	Ltifi 2025	Inhibition	Inhibition score	Immediate post (12 weeks)	27/27	0.314	-0.223 to 0.850	Some concerns	10.0%
ST079_I	ST079 [40]	Wu 2026	Inhibition	Peg Tapping score	Immediate post	98/17	0.976	0.446 to 1.506	Serious	10.1%
ST081_I	ST081 [39]	Shen 2020	Inhibition	Go/No-Go score	Immediate post	30/30	0.904	0.372 to 1.436	Moderate	10.0%
ST082_I	ST082 [32]	Bai 2022	Inhibition	Silly Sound Stroop behavioral-performance score	Immediate post	30/32	0.956	0.429 to 1.482	Some concerns	10.1%
ST096_I	ST096 [35]	Vazou 2021	Inhibition	Cool EF / Day–Night accuracy	Immediate post	53/52	-0.017	-0.400 to 0.366	Some concerns	11.5%
ST100_I	ST100 [15]	Suppalarkbunlue 2023	Inhibition	Cat–Mouse no-go accuracy	Immediate post	39/40	0.261	-0.182 to 0.704	Moderate	10.9%
ST114_I	ST114 [44]	Degé 2022	Inhibition	NEPSY-II Statue score	Immediate post	11/14	0.549	-0.257 to 1.354	Some concerns	7.5%
ST005_WM	ST005 [27]	Zhang 2024	Working memory	Keep Track Task score	Immediate post (12 weeks)	31/32	0.627	0.121 to 1.134	Some concerns	10.1%
ST026_WM	ST026 [38]	Ilari 2021	Working memory	Spin the Pots score (16 − errors)	Immediate post (5 weeks)	51/52	-0.162	-0.549 to 0.224	Moderate	11.7%
ST070_WM	ST070 [36]	Chiu 2026	Working memory	Adapted working-memory task total score (age strata aggregated)	Immediate post (8 weeks)	19/14	-0.490	-1.190 to 0.211	Serious	7.7%
ST072_WM	ST072 [13]	Shen 2019	Working memory	Backward Digit Span score	Immediate post (T2)	30/31	0.884	0.357 to 1.411	Moderate	9.8%
ST076_WM	ST076 [31]	Ltifi 2025	Working memory	Working-memory score	Immediate post (12 weeks)	27/27	0.113	-0.421 to 0.646	Some concerns	9.7%
ST079_WM	ST079 [40]	Wu 2026	Working memory	Rotating Can score	Immediate post	98/17	0.797	0.272 to 1.322	Serious	9.8%
ST081_WM	ST081 [39]	Shen 2020	Working memory	Backward Digit Span score	Immediate post	30/30	0.651	0.131 to 1.170	Moderate	9.9%
ST082_WM	ST082 [32]	Bai 2022	Working memory	Empty House behavioral-performance score	Immediate post	30/32	0.844	0.323 to 1.364	Some concerns	9.9%
ST100_WM	ST100 [15]	Suppalarkbunlue 2023	Working memory	Missing Scan Task score	Immediate post	39/40	0.163	-0.278 to 0.605	Moderate	11.0%
ST107_WM	ST107 [42]	Hsiao 2025	Working memory	Chinese Working Memory Test for Young Children	Immediate post (360 min total)	35/32	0.683	0.190 to 1.177	Moderate	10.3%
ST005_CF	ST005 [27]	Zhang 2024	Cognitive flexibility	Dots Task accuracy	Immediate post (12 weeks)	31/32	0.694	0.185 to 1.203	Some concerns	11.2%
ST026_CF	ST026 [38]	Ilari 2021	Cognitive flexibility	Dimensional Change Card Sort score	Immediate post (5 weeks)	51/52	0.422	0.031 to 0.812	Moderate	12.6%
ST061_CF	ST061 [30]	Xiong 2017	Cognitive flexibility	Preschool Executive Function Measure total score	Immediate post (3 months)	19/20	1.067	0.394 to 1.741	Serious	9.3%
ST072_CF	ST072 [13]	Shen 2019	Cognitive flexibility	Dimensional Change Card Sort score	Immediate post (T2)	30/31	0.927	0.398 to 1.456	Moderate	11.0%
ST079_CF	ST079 [40]	Wu 2026	Cognitive flexibility	Dimensional Change Card Sort score	Immediate post	98/17	0.525	0.005 to 1.044	Serious	11.1%
ST081_CF	ST081 [39]	Shen 2020	Cognitive flexibility	Dimensional Change Card Sort score	Immediate post	30/30	0.914	0.382 to 1.447	Moderate	10.9%
ST082_CF	ST082 [32]	Bai 2022	Cognitive flexibility	DCCS shifting behavioral-performance score	Immediate post	30/32	1.080	0.545 to 1.614	Some concerns	10.9%
ST092_CF	ST092 [33]	Xiong 2019	Cognitive flexibility	DCCS executive-function score	Immediate post (8 weeks)	30/30	0.499	-0.015 to 1.013	Some concerns	11.1%
ST100_CF	ST100 [15]	Suppalarkbunlue 2023	Cognitive flexibility	DCCS border score	Immediate post	39/40	-0.497	-0.944 to -0.049	Moderate	11.9%

Supplementary Table S7. SMD-specific small-study diagnostics

For domains with k >= 10, Hedges g was regressed on the sample-size precision term sqrt(1/nI + 1/nC) using REML meta-regression with Hartung-Knapp inference. Conventional SMD-versus-standard-error Egger regression was not used because the SMD and its usual standard error are mathematically correlated.

Domain	k	Fixed g	Random g	Difference	Modified slope	95% CI	p	Residual tau2	Interpretation
Inhibition	10	0.464	0.525	0.061	2.379	-5.964 to 10.722	0.529	0.216	No detected small-study association; low power.
Working memory	10	0.400	0.419	0.019	-2.197	-11.983 to 7.589	0.619	0.162	No detected small-study association; low power.
Cognitive flexibility	9	0.546	0.604	0.058	Not tested	k<10	—	—	Formal asymmetry test withheld.

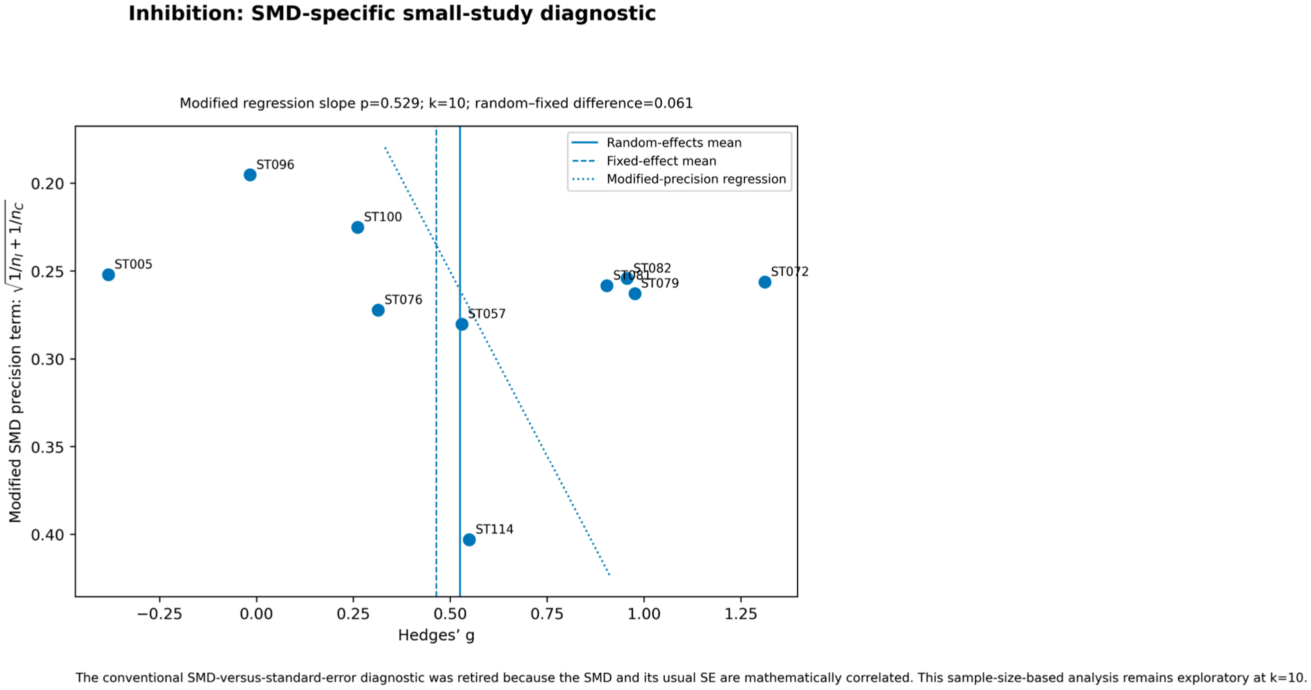

Figure S1A. Inhibition SMD-specific modified-precision diagnostic. Modified slope p=0.529; k=10.

Working memory: SMD-specific small-study diagnostic

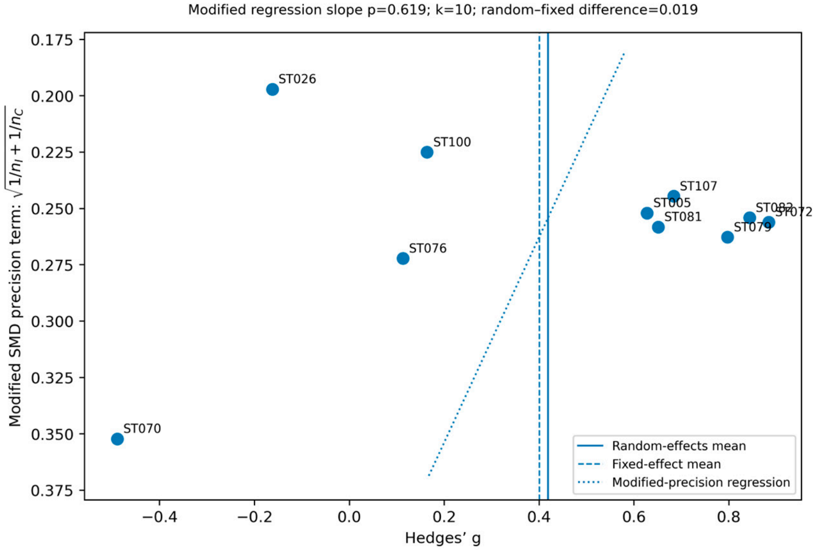

The conventional SMD-versus-standard-error diagnostic was retired because the SMD and its usual SE are mathematically correlated. This sample-size-based analysis remains exploratory at k=10.

Figure S1B. Working-memory SMD-specific modified-precision diagnostic. Modified slope p=0.619; k=10.

Supplementary Table S8. ROB-ME evidence matrix and domain assessments

Study ID	Ref.	Author/year	Inhibition	Working memory	Cognitive flexibility
ST005	27	Zhang 2024	A — available and included	A — available and included	A — available and included
ST012	11	Biino 2023	P — partial/non-model-ready; hard-gate exclusion	P — partial/non-model-ready; hard-gate exclusion	P — partial/non-model-ready; hard-gate exclusion
ST026	38	Ilari 2021	NE — no eligible result for this domain	A — available and included	A — available and included
ST057	28	Başarır 2025	A — available and included	NE — no eligible result for this domain	NE — no eligible result for this domain
ST060	29	He 2025	P — partial/non-model-ready; hard-gate exclusion	P — partial/non-model-ready; hard-gate exclusion	P — partial/non-model-ready; hard-gate exclusion
ST061	30	Xiong 2017	NE — no eligible result for this domain	NE — no eligible result for this domain	A — available and included
ST070	36	Chiu 2026	NE — no eligible result for this domain	A — available and included	NE — no eligible result for this domain
ST072	13	Shen 2019	A — available and included	A — available and included	A — available and included
ST076	31	Ltifi 2025	A — available and included	A — available and included	NE — no eligible result for this domain
ST079	40	Wu 2026	A — available and included	A — available and included	A — available and included
ST081	39	Shen 2020	A — available and included	A — available and included	A — available and included
ST082	32	Bai 2022	A — available and included	A — available and included	A — available and included
ST092	33	Xiong 2019	NE — no eligible result for this domain	NE — no eligible result for this domain	A — available and included
ST096	35	Vazou 2021	A — available and included	NE — no eligible result for this domain	NE — no eligible result for this domain
ST097	37	Díaz-Quesada 2025	P — partial/non-model-ready; hard-gate exclusion	P — partial/non-model-ready; hard-gate exclusion	P — partial/non-model-ready; hard-gate exclusion
ST098	41	Çiftçi 2022	P — partial/non-model-ready; hard-gate exclusion	P — partial/non-model-ready; hard-gate exclusion	P — partial/non-model-ready; hard-gate exclusion

Study ID	Ref.	Author/year	Inhibition	Working memory	Cognitive flexibility
ST100	15	Suppalarkbunlue 2023	A — available and included	A — available and included	A — available and included
ST107	42	Hsiao 2025	NE — no eligible result for this domain	A — available and included	NE — no eligible result for this domain
ST109	34	Miller 2023	NE — no eligible result for this domain	P — partial/non-model-ready; hard-gate exclusion	P — partial/non-model-ready; hard-gate exclusion
ST113	43	Bugos 2017	P — partial/non-model-ready; hard-gate exclusion	NE — no eligible result for this domain	NE — no eligible result for this domain
ST114	44	Degé 2022	A — available and included	NE — no eligible result for this domain	NE — no eligible result for this domain

Table S8.1. ROB-ME assessment — Inhibition

Signalling question	Final response	Supporting evidence
Q1. Among identified eligible studies, were any results unavailable likely because of the P value, magnitude, or direction?	Probably no	5 of 15 eligible candidate results were excluded because an exact, unique model-ready effect and variance could not be reconstructed without assumptions. Exclusion was not conditioned on statistical significance or direction, and preserved provisional directions were not uniformly unfavourable.
Q2. If Q1 is Yes/Probably yes, would inclusion of those omitted results likely change the synthesis notably?	Not applicable	Q1 was not answered Yes/Probably yes. No assumption-based impact model was fabricated.
Q3. Were there identified studies for which it was unclear whether an eligible result had been generated?	No	Domain eligibility and result generation could be classified for all 21 candidate projects. The hard-gate problem was incomplete model-ready reporting, not uncertainty over whether the domain was assessed.
Q4. If Q3 is Yes/Probably yes, would inclusion of the potentially omitted results likely change the synthesis notably?	Not applicable	Q3 was answered No.
Q5. Do review circumstances indicate potential for eligible studies not being identified because of their results?	Probably yes	The review was not restricted to an inception cohort. Seven bibliographic databases were searched, but a preserved dedicated trial-register/grey-literature search was unavailable; 13 reports were not retrieved or not assessable, and exact source-specific execution records were not preserved.
Q6. If Q5 is Yes/Probably yes, is it likely that unidentified studies had results eligible for this domain-specific synthesis?	No information	Unidentified intervention studies could plausibly contain executive-function outcomes, but there is no reliable basis to determine whether they generated a result eligible for this exact domain and timepoint.
Q7. Does the observed pattern suggest that missing studies/results were systematically different from those observed?	Probably no	The SMD-specific modified-precision regression was not statistically notable (slope p=0.529); the random- and fixed-effect estimates differed by 0.061. With k=10 and mixed randomized/non-randomized designs, this is low-power supportive evidence only.
Q8. Do appropriate sensitivity analyses indicate that the pooled estimate was biased because of missing evidence?	No information	No validated selection model or assumption-based missing-result analysis was justified. Leave-one-study-out and risk-of-bias sensitivity analyses address influence and study quality, not missing evidence itself.

Table S8.2. ROB-ME assessment — Working memory

Signalling question	Final response	Supporting evidence
Q1. Among identified eligible studies, were any results unavailable likely because of the P value, magnitude, or direction?	Probably no	5 of 15 eligible candidate results were excluded because an exact, unique model-ready effect and variance could not be reconstructed without assumptions. Exclusion was not conditioned on statistical significance or direction, and preserved provisional directions were not uniformly unfavourable.
Q2. If Q1 is Yes/Probably yes, would inclusion of those omitted results likely change the synthesis notably?	Not applicable	Q1 was not answered Yes/Probably yes. No assumption-based impact model was fabricated.
Q3. Were there identified studies for which it was unclear whether an eligible result had been generated?	No	Domain eligibility and result generation could be classified for all 21 candidate projects. The hard-gate problem was incomplete model-ready reporting, not uncertainty over whether the domain was assessed.
Q4. If Q3 is Yes/Probably yes, would inclusion of the potentially omitted results likely change the synthesis notably?	Not applicable	Q3 was answered No.
Q5. Do review circumstances indicate potential for eligible studies not being identified because of their results?	Probably yes	The review was not restricted to an inception cohort. Seven bibliographic databases were searched, but a preserved dedicated trial-register/grey-literature search was unavailable; 13 reports were not retrieved or not assessable, and exact source-specific execution records were not preserved.
Q6. If Q5 is Yes/Probably yes, is it likely that unidentified studies had results eligible for this domain-specific synthesis?	No information	Unidentified intervention studies could plausibly contain executive-function outcomes, but there is no reliable basis to determine whether they generated a result eligible for this exact domain and timepoint.
Q7. Does the observed pattern suggest that missing studies/results were systematically different from those observed?	Probably no	The SMD-specific modified-precision regression was not statistically notable (slope p=0.619); the random- and fixed-effect estimates differed by 0.019. With k=10 and mixed randomized/non-randomized designs, this is low-power supportive evidence only.
Q8. Do appropriate sensitivity analyses indicate that the pooled estimate was biased because of missing evidence?	No information	No validated selection model or assumption-based missing-result analysis was justified. Leave-one-study-out and risk-of-bias sensitivity analyses address influence and study quality, not missing evidence itself.

Table S8.3. ROB-ME assessment — Cognitive flexibility

Signalling question	Final response	Supporting evidence
Q1. Among identified eligible studies, were any results unavailable likely because of the P value, magnitude, or direction?	Probably no	5 of 14 eligible candidate results were excluded because an exact, unique model-ready effect and variance could not be reconstructed without assumptions. Exclusion was not conditioned on statistical significance or direction, and preserved provisional directions were not uniformly unfavourable.

Signalling question	Final response	Supporting evidence
Q2. If Q1 is Yes/Probably yes, would inclusion of those omitted results likely change the synthesis notably?	Not applicable	Q1 was not answered Yes/Probably yes. No assumption-based impact model was fabricated.
Q3. Were there identified studies for which it was unclear whether an eligible result had been generated?	No	Domain eligibility and result generation could be classified for all 21 candidate projects. The hard-gate problem was incomplete model-ready reporting, not uncertainty over whether the domain was assessed.
Q4. If Q3 is Yes/Probably yes, would inclusion of the potentially omitted results likely change the synthesis notably?	Not applicable	Q3 was answered No.
Q5. Do review circumstances indicate potential for eligible studies not being identified because of their results?	Probably yes	The review was not restricted to an inception cohort. Seven bibliographic databases were searched, but a preserved dedicated trial-register/grey-literature search was unavailable; 13 reports were not retrieved or not assessable, and exact source-specific execution records were not preserved.
Q6. If Q5 is Yes/Probably yes, is it likely that unidentified studies had results eligible for this domain-specific synthesis?	No information	Unidentified intervention studies could plausibly contain executive-function outcomes, but there is no reliable basis to determine whether they generated a result eligible for this exact domain and timepoint.
Q7. Does the observed pattern suggest that missing studies/results were systematically different from those observed?	No information	No formal asymmetry test was undertaken because k=9<10. The random- and fixed-effect estimates differed by 0.058, but this cannot exclude systematically missing evidence.
Q8. Do appropriate sensitivity analyses indicate that the pooled estimate was biased because of missing evidence?	No information	No validated selection model or assumption-based missing-result analysis was justified. Leave-one-study-out and risk-of-bias sensitivity analyses address influence and study quality, not missing evidence itself.

Supplementary Table S9. GRADE evidence profile and Summary of Findings

GRADE was applied to the three core quantitative outcomes. The ratings below were finalized through structured review-team evidence matrices and methodologist adjudication; no duplicate independent assessment is claimed.

Outcome	Participants (studies)	Effect	Risk of bias	Inconsistency	Indirectness	Imprecision	Publication bias	Certainty
Inhibition	675 (10)	g=0.52 (0.15 to 0.90)	Serious (−1)	Serious (−1)	Not serious	Not serious	Not downgraded	Low ⨁⨁◯◯
Working memory	697 (10)	g=0.42 (0.09 to 0.75)	Serious (−1)	Serious (−1)	Not serious	Not serious	Not downgraded	Low ⨁⨁◯◯
Cognitive flexibility	642 (9)	g=0.60 (0.22 to 0.98)	Serious (−1)	Serious (−1)	Not serious	Not serious	Not downgraded	Low ⨁⨁◯◯

Table S9.1. Detailed GRADE judgments — Inhibition

GRADE domain	Judgment	Rationale
Risk of bias	Serious (−1)	Random-effects weight: Some concerns 59.2%, Moderate 30.8%, Serious 10.1%. Excluding Serious judgments retained a favourable estimate (g=0.47, 95% CI 0.07 to 0.88); therefore the limitation was judged serious, not very serious.
Inconsistency	Serious (−1)	I^2=75.0%, τ^2=0.207, and the 95% prediction interval (-0.57 to 1.62) crossed the null. Leave-one-study-out analyses retained the direction of the pooled mean, so inconsistency was not rated very serious.
Indirectness	Not serious	The evidence directly addressed eligible early-childhood interventions, comparators and the prespecified domain. Task variation was handled using SMDs and one selected effect per Study ID/domain/timepoint.
Imprecision	Not serious	675 participants exceeded the approximate 400-participant caution threshold for an SMD of 0.20, and the HKSJ interval (0.15 to 0.90) excluded the null. Prediction-interval uncertainty was counted under inconsistency and was not double-counted.
Publication bias	Not downgraded	ROB-ME was Some concerns, but no high-likelihood directional missingness was demonstrated. The modified SMD diagnostic did not detect a small-study association (p=0.529). Concern remains explicit but did not meet the threshold for a separate GRADE downgrade.
Overall certainty	Low ⨁⨁◯◯	Structured movement- and arts-based interventions may improve inhibition on average, but confidence in the magnitude and transportability of the effect is limited.

Table S9.2. Detailed GRADE judgments — Working memory

GRADE domain	Judgment	Rationale
Risk of bias	Serious (−1)	Random-effects weight: Some concerns 29.7%, Moderate 52.7%, Serious 17.6%. Excluding Serious judgments retained a favourable estimate (g=0.46, 95% CI 0.13 to 0.78); therefore the limitation was judged serious, not very serious.
Inconsistency	Serious (−1)	I^2=67.1%, τ^2=0.134, and the 95% prediction interval (-0.47 to 1.31) crossed the null. Leave-one-study-out analyses retained the direction of the pooled mean, so inconsistency was not rated very serious.
Indirectness	Not serious	The evidence directly addressed eligible early-childhood interventions, comparators and the prespecified domain. Task variation was handled using SMDs and one selected effect per Study ID/domain/timepoint.
Imprecision	Not serious	697 participants exceeded the approximate 400-participant caution threshold for an SMD of 0.20, and the HKSJ interval (0.09 to 0.75) excluded the null. Prediction-interval uncertainty was counted under inconsistency and was not double-counted.
Publication bias	Not downgraded	ROB-ME was Some concerns, but no high-likelihood directional missingness was demonstrated. The modified SMD diagnostic did not detect a small-study association (p=0.619). Concern remains explicit but did not meet the threshold for a separate GRADE downgrade.

GRADE domain	Judgment	Rationale
Overall certainty	Low ⊕⊕◯◯	Structured movement- and arts-based interventions may improve working memory on average, but confidence in the magnitude and transportability of the effect is limited.

Table S9.3. Detailed GRADE judgments — Cognitive flexibility

GRADE domain	Judgment	Rationale
Risk of bias	Serious (−1)	Random-effects weight: Some concerns 33.2%, Moderate 46.4%, Serious 20.4%. Excluding Serious judgments retained a favourable estimate (g=0.56, 95% CI 0.07 to 1.06); therefore the limitation was judged serious, not very serious.
Inconsistency	Serious (−1)	I^2=73.5%, τ^2=0.184, and the 95% prediction interval (-0.46 to 1.66) crossed the null. Leave-one-study-out analyses retained the direction of the pooled mean, so inconsistency was not rated very serious.
Indirectness	Not serious	The evidence directly addressed eligible early-childhood interventions, comparators and the prespecified domain. Task variation was handled using SMDs and one selected effect per Study ID/domain/timepoint.
Imprecision	Not serious	642 participants exceeded the approximate 400-participant caution threshold for an SMD of 0.20, and the HKSJ interval (0.22 to 0.98) excluded the null. Prediction-interval uncertainty was counted under inconsistency and was not double-counted.
Publication bias	Not downgraded	ROB-ME was Some concerns, but no high-likelihood directional missingness was demonstrated. Formal asymmetry testing was not performed because k<10. Concern remains explicit but did not meet the threshold for a separate GRADE downgrade.
Overall certainty	Low ⊕⊕◯◯	Structured movement- and arts-based interventions may improve cognitive flexibility on average, but confidence in the magnitude and transportability of the effect is limited.

Supplementary Table S10. Original-versus-amended protocol and methodological decision history

The original protocol is publicly available through PROSPERO and has not been retrospectively modified. This table distinguishes prospectively registered content, post-completion amendments and peer-review sensitivity analyses.

Element	Version 1.0 / original protocol	Later amendment or final implementation	Timing/classification	Editorial consequence
Review question and age window	Central question; exposure from birth through 72 months; later outcomes allowed.	Core question and age window unchanged.	Prospectively registered.	Report as prespecified.
Exposure taxonomy	E1 movement/physical activity; E2 arts; E3 mixed exposure.	Same framework retained; Slot classified as contextual E2 dramatic/sociodramatic play.	Prospectively registered; later operational application.	Explain contextual, narrative-only Slot role.
Information sources	PubMed, Web of Science/SCI, Scopus; supplementary Google Scholar/citation checking planned.	Seven formal databases: PubMed/MEDLINE, Web of Science, Scopus, ERIC, PSICODOC, CINAHL, SPORTDiscus.	Version 2.0 post-completion amendment.	Do not present the seven-source implementation as fully prospective.
Outcome priority	Executive function and visuospatial outcomes broadly eligible.	Inhibition, working memory and cognitive flexibility became the quantitative core; global/self-regulation exploratory; visuospatial evidence narrative/sensitivity.	Feasibility/construct decision formalised during finalisation.	Decision based on construct and data compatibility, not result magnitude.
Effect-selection hierarchy	General SMD synthesis planned.	One verified immediate-post effect per Study ID/domain/time; deterministic composites excluded; alternative tasks reserved for sensitivity.	Operational hierarchy finalised during extraction and reproducibility audit.	Provide row-level rationale in Table S11.
Synthesis model	Random-effects synthesis with heterogeneity and prediction intervals when feasible.	REML-Hartung-Knapp, 15 Study IDs/29 effects, three core domains.	Analytic refinement and post-submission version-control correction.	Historical 21/49 file identified as candidate matrix.
Reporting bias	Funnel/Egger/rank-correlation approach described in v2.0.	ROB-ME plus SMD-specific modified-precision diagnostic; no formal test at k<10.	Post-registration methodological correction.	Conventional SMD-vs-SE regression retired.
Age and clustering	Not specified beyond broad age eligibility and design-specific RoB.	Study-level age audit, younger-inclusive exclusion, ICC scenarios and extreme cluster exclusion.	Peer-review sensitivity analyses.	Report as post-completion robustness analyses.
Protocol availability and timeline	Protocol uploaded but initially withheld until anticipated end date.	Original protocol made public; end date corrected to 10 July 2026.	Minor amendments during peer-review revision.	Public accessibility verified through PROSPERO.

Supplementary Table S11. Study/effect extraction, developmental age, design, clustering and outcome-selection register

Table S11A provides study-level age and unit-of-analysis information. Table S11B identifies the selected quantitative effect and selection rule for every row in the frozen 29-effect dataset.

Table S11A. Study-level age, design and clustering audit

Study ID	Author/year	Domains	Exposure age	Age band	Design/allocation	Cluster adjustment status	Final audit decision	DOI
ST005	Zhang 2024	Inhibition; Working memory; Cognitive flexibility	Intervention 66.27 (SD 3.12) months; control 66.79 (SD 3.34) months	48–72	Individually randomized controlled trial; allocation: Child	Not applicable;	Retain without cluster correction	10.1002/pchj.786
ST026	Ilari 2021	Working memory; Cognitive flexibility	Mean 4.7 years (SD 0.47), range 4–6 years	48–72	Individual random assignment within multi-class school sample; allocation: Child	Class ICCs reported; variance sensitivity required; ICC .08 for WM/inhibition; .04 for CF; DE 2.294/1.647	Retain; apply observed-ICC variance inflation in sensitivity	10.1177/20592043211054829
ST057	Başarır 2025	Inhibition	Reported age 5–6 years; mean 6.03 years (SD 0.30)	48–72 (upper-window borderline)	Individually randomized controlled trial; allocation: Child	Not applicable;	Retain without cluster correction	10.1186/s12887-025-05897-x
ST061	Xiong 2017	Cognitive flexibility	Age 4–5 years; mean 4.67 years	48–72	Class-assigned controlled intervention / quasi-experimental; allocation: Intact class	No cluster-adjusted estimate or ICC reported; Scenario ICC .01/.05/.10	Retain primary effect with explicit unit-of-analysis limitation; include scenario/exclusion sensitivity	10.1155/2017/5635070
ST070	Chiu 2026	Working memory	Preschool sample aged 4–6 years	48–72	Classroom quasi-experimental intervention; allocation: Existing classroom	No cluster adjustment reported; Scenario ICC .01/.05/.10	Retain primary effect with limitation; include scenario/exclusion sensitivity	10.3390/children13030319
ST072	Shen 2019	Inhibition; Working memory; Cognitive flexibility	Music 51.39 (SD 4.27) months; control 50.35 (SD 3.38) months	48–72	Natural-class controlled intervention; allocation: Intact class	No cluster adjustment reported; Scenario ICC .01/.05/.10	Retain primary effects with limitation; include scenario/exclusion sensitivity	10.3389/fpsyg.2019.01910
ST076	Ltifi 2025	Inhibition; Working memory	Mean 3.87 years (SD 0.47) = 46.44 months	Younger-inclusive / mixed around 48 months	Individually randomized controlled trial; allocation: Child	Not applicable;	Retain without cluster correction	10.3390/children12101405
ST079	Wu 2026	Inhibition; Working memory; Cognitive flexibility	Mean 59.02 months (SD 3.47), age 4–5 years	48–72	Individually randomized multi-arm trial with classroom context; allocation: Child	Source reports classroom random-effect sensitivity confirming results; Source sensitivity; no additional inflation imposed	Retain; source-level classroom sensitivity considered adequate	10.3389/fpsyg.2026.1779286
ST081	Shen 2020	Inhibition; Working memory; Cognitive flexibility	Dance 52.40 (SD 3.95) months; control 55.68 (SD 4.23) months	48–72	Natural-class controlled intervention; allocation: Intact class	No cluster adjustment reported; Scenario ICC .01/.05/.10	Retain primary effects with limitation; include scenario/exclusion sensitivity	10.3389/fpsyg.2020.585598
ST082	Bai 2022	Inhibition; Working memory; Cognitive flexibility	Age 4–5 years; intervention mean 4.16 (SD 0.29), control 4.70 (SD 0.43) years	48–72	Pilot cluster-randomized controlled trial; allocation: Class	Repeated-measures ANOVA without cluster adjustment; ICC not reported; Scenario ICC .01/.05/.10	Retain with major unit-of-analysis limitation; include scenario/exclusion sensitivity	10.3389/fpsyg.2022.847785
ST092	Xiong 2019	Cognitive flexibility	Age 4–5 years	48–72	Cluster-randomized pilot trial; allocation: Class	No cluster adjustment reported; Scenario ICC .01/.05/.10	Retain primary effect with limitation; include scenario/exclusion sensitivity	10.3390/jcm8040469
ST096	Vazou 2021	Inhibition	Age 3–5 years; mean 4.22 years = 50.64 months	Younger-inclusive mixed 36–60	Cluster/classroom-randomized feasibility trial; allocation: Classroom	Core Day–Night SMD derived from descriptive data; cluster-adjusted source estimate unavailable; Scenario ICC .01/.05/.10	Retain with unit-of-analysis limitation; include scenario/exclusion sensitivity	10.3389/fpsyg.2021.729272
ST100	Suppalarkbunlue 2023	Inhibition; Working memory; Cognitive flexibility	Age 4–5 years; intervention mean 4.36 (SD 0.42), control 4.49 (SD 0.29) years	48–72	Classroom quasi-experimental intervention; allocation: Classroom/centre grouping	No cluster adjustment reported; Scenario ICC .01/.05/.10; m=20 assumption	Retain with explicit source/cluster-count limitation; include scenario/exclusion sensitivity	10.1080/02568543.2022.2111482
ST107	Hsiao 2025	Working memory	Kindergarten level 3, ages 5–6 years	48–72	Quasi-experimental individual allocation within multi-class sample; allocation: Child	No class random effect reported; groups were formed at child level; Residual class-context limitation; not in strict cluster-exclusion set	Retain; disclose residual class-context concern	10.1007/s11423-025-10471-4
ST114	Degé 2022	Inhibition	Mean 71.44 months (SD 4.91)	48–72 (predominantly 60–72)	Individually randomized active-comparator trial; allocation: Child	Not applicable;	Retain without cluster correction	10.1177/1029864920938432

Table S11B. Effect-level extraction and outcome-selection register

Effect ID	Study ID/ref.	Author/year	Domain	Selected outcome	Timepoint	Contrast	nI/nC	g	95% CI	Age/design note	Selection rule applied	Alternative/secondary handling	DOI
ST005_I	ST005 / 27	Zhang 2024	Inhibition	Stop Signal Task accuracy	Immediate post (12 weeks)	Recreational gymnastics vs usual outdoor/no organized sport	31/32	-0.38	-0.88 to 0.12	Intervention 66.27 (SD 3.12) months; control 66.79 (SD 3.34) months; Individually randomized controlled trial	Direct core-domain construct; immediate post; one effect per Study ID/domain/time; independent within domain; not selected by magnitude or significance.	Hot-EF/delay-gratification tasks retained narratively; no single favourable task selected as a global effect.	10.1002/pchj.786
ST057_I	ST057 / 28	Başarır 2025	Inhibition	Go/No-Go response time	Immediate post (8 weeks)	Coordination-based training vs routine programme	26/25	0.53	-0.03 to 1.09	Reported age 5–6 years; mean 6.03 years (SD 0.30); Individually randomized controlled trial	Direct core-domain construct; immediate post; one effect per Study ID/domain/time; independent within domain; not selected by magnitude or significance.		10.1186/s12887-025-05897-x
ST072_I	ST072 / 13	Shen 2019	Inhibition	Day/Night Stroop score	Immediate post (T2)	Music training vs free-play control	30/31	1.31	0.76 to 1.87	Music 51.39 (SD 4.27) months; control 50.35 (SD 3.38) months; Natural-class controlled intervention	Direct core-domain construct; immediate post; one effect per Study ID/domain/time; independent within domain; not selected by magnitude or significance.	Dot Matrix retained only as working-memory task-selection sensitivity.	10.3389/fpsyg.2019.01910
ST076_I	ST076 / 31	Ltifi 2025	Inhibition	Inhibition score	Immediate post (12 weeks)	Mini-trampoline training vs usual physical activity	27/27	0.31	-0.22 to 0.85	Mean 3.87 years (SD 0.47) = 46.44 months; Individually randomized controlled trial	Direct core-domain construct; immediate post; one effect per Study ID/domain/time; independent within domain; not selected by magnitude or significance.		10.3390/children12101405
ST079_I	ST079 / 40	Wu 2026	Inhibition	Peg Tapping score	Immediate post	Aggregated music-style intervention vs control	98/17	0.98	0.45 to 1.51	Mean 59.02 months (SD 3.47), age 4–5 years; Individually randomized multi-arm trial with classroom context	Direct core-domain construct; immediate post; one effect per Study ID/domain/time; independent within domain; not selected by magnitude or significance.		10.3389/fpsyg.2026.1779286
ST081_I	ST081 / 39	Shen 2020	Inhibition	Go/No-Go score	Immediate post	Street-dance training vs free play/no training	30/30	0.90	0.37 to 1.44	Dance 52.40 (SD 3.95) months; control 55.68 (SD 4.23) months; Natural-class controlled intervention	Direct core-domain construct; immediate post; one effect per Study ID/domain/time; independent within domain; not selected by magnitude or significance.		10.3389/fpsyg.2020.585598
ST082_I	ST082 / 32	Bai 2022	Inhibition	Silly Sound Stroop behavioral-performance score	Immediate post	Group-play MVPA vs usual exercise	30/32	0.96	0.43 to 1.48	Age 4–5 years; intervention mean 4.16 (SD 0.29), control 4.70 (SD 0.43) years; Pilot cluster-randomized controlled trial	Direct core-domain construct; immediate post; one effect per Study ID/domain/time; independent within domain; not selected by magnitude or significance.	Overall EF composite excluded as a deterministic duplicate of the three component domains.	10.3389/fpsyg.2022.847785
ST096_I	ST096 / 35	Vazou 2021	Inhibition	Cool EF / Day–Night accuracy	Immediate post	Cognitively engaging physical activity vs routine curriculum	53/52	-0.02	-0.40 to 0.37	Age 3–5 years; mean 4.22 years = 50.64 months; Cluster/classroom-randomized feasibility trial	Direct core-domain construct; immediate post; one effect per Study ID/domain/time; independent within domain; not selected by magnitude or significance.	Teacher-rated behavioural control retained as source-preferred cluster-adjusted narrative MD.	10.3389/fpsyg.2021.729272
ST100_I	ST100 / 15	Suppalarkbunlue 2023	Inhibition	Cat–Mouse no-go accuracy	Immediate post	Music–movement activities vs active classroom comparator	39/40	0.26	-0.18 to 0.70	Age 4–5 years; intervention mean 4.36 (SD 0.42), control 4.49 (SD 0.29) years; Classroom quasi-experimental intervention	Direct core-domain construct; immediate post; one effect per Study ID/domain/time; independent within domain; not selected		10.1080/02568543.2022.2111482

Effect ID	Study ID/ref.	Author/year	Domain	Selected outcome	Timepoint	Contrast	nI/nC	g	95% CI	Age/design note	Selection rule applied	Alternative/secondary handling	DOI
											by magnitude or significance.		
ST114_I	ST114 / 44	Degé 2022	Inhibition	NEPSY-II Statue score	Immediate post	Music training vs active sports control	11/14	0.55	-0.26 to 1.35	Mean 71.44 months (SD 4.91); Individually randomized active-comparator trial	Direct core-domain construct; immediate post; one effect per Study ID/domain/time; independent within domain; not selected by magnitude or significance.		10.1177/1029864920938432
ST005_WM	ST005 / 27	Zhang 2024	Working memory	Keep Track Task score	Immediate post (12 weeks)	Recreational gymnastics vs usual outdoor/no organized sport	31/32	0.63	0.12 to 1.13	Intervention 66.27 (SD 3.12) months; control 66.79 (SD 3.34) months; Individually randomized controlled trial	Direct core-domain construct; immediate post; one effect per Study ID/domain/time; independent within domain; not selected by magnitude or significance.	Hot-EF/delay-gratification tasks retained narratively; no single favourable task selected as a global effect.	10.1002/pchj.786
ST026_WM	ST026 / 38	Ilari 2021	Working memory	Spin the Pots score (16 – errors)	Immediate post (5 weeks)	Musical activities vs control	51/52	-0.16	-0.55 to 0.22	Mean 4.7 years (SD 0.47), range 4–6 years; Individual random assignment within multi-class school sample	Direct core-domain construct; immediate post; one effect per Study ID/domain/time; independent within domain; not selected by magnitude or significance.		10.1177/20592043211054829
ST070_WM	ST070 / 36	Chiu 2026	Working memory	Adapted working-memory task total score (age strata aggregated)	Immediate post (8 weeks)	Programmed physical activity vs usual unstructured gross-motor activity	19/14	-0.49	-1.19 to 0.21	Preschool sample aged 4–6 years; Classroom quasi-experimental intervention	Direct core-domain construct; immediate post; one effect per Study ID/domain/time; independent within domain; not selected by magnitude or significance.		10.3390/children13030319
ST072_WM	ST072 / 13	Shen 2019	Working memory	Backward Digit Span score	Immediate post (T2)	Music training vs free-play control	30/31	0.88	0.36 to 1.41	Music 51.39 (SD 4.27) months; control 50.35 (SD 3.38) months; Natural-class controlled intervention	Direct core-domain construct; immediate post; one effect per Study ID/domain/time; independent within domain; not selected by magnitude or significance.	Dot Matrix retained only as working-memory task-selection sensitivity.	10.3389/fpsyg.2019.01910
ST076_WM	ST076 / 31	Ltifi 2025	Working memory	Working-memory score	Immediate post (12 weeks)	Mini-trampoline training vs usual physical activity	27/27	0.11	-0.42 to 0.65	Mean 3.87 years (SD 0.47) = 46.44 months; Individually randomized controlled trial	Direct core-domain construct; immediate post; one effect per Study ID/domain/time; independent within domain; not selected by magnitude or significance.		10.3390/children12101405
ST079_WM	ST079 / 40	Wu 2026	Working memory	Rotating Can score	Immediate post	Aggregated music-style intervention vs control	98/17	0.80	0.27 to 1.32	Mean 59.02 months (SD 3.47), age 4–5 years; Individually randomized multi-arm trial with classroom context	Direct core-domain construct; immediate post; one effect per Study ID/domain/time; independent within domain; not selected by magnitude or significance.		10.3389/fpsyg.2026.1779286
ST081_WM	ST081 / 39	Shen 2020	Working memory	Backward Digit Span score	Immediate post	Street-dance training vs free play/no training	30/30	0.65	0.13 to 1.17	Dance 52.40 (SD 3.95) months; control 55.68 (SD 4.23) months; Natural-class controlled intervention	Direct core-domain construct; immediate post; one effect per Study ID/domain/time; independent within domain; not selected by magnitude or significance.		10.3389/fpsyg.2020.585598
ST082_WM	ST082 / 32	Bai 2022	Working memory	Empty House behavioral-performance score	Immediate post	Group-play MVPA vs usual exercise	30/32	0.84	0.32 to 1.36	Age 4–5 years; intervention mean 4.16 (SD 0.29), control 4.70 (SD 0.43) years; Pilot cluster-randomized controlled trial	Direct core-domain construct; immediate post; one effect per Study ID/domain/time; independent within domain; not selected	Overall EF composite excluded as a deterministic duplicate of the three component domains.	10.3389/fpsyg.2022.847785

Effect ID	Study ID/ref.	Author/year	Domain	Selected outcome	Timepoint	Contrast	nI/nC	g	95% CI	Age/design note	Selection rule applied	Alternative/secondary handling	DOI
											by magnitude or significance.		
ST100_WM	ST100 / 15	Suppalarkbunlue 2023	Working memory	Missing Scan Task score	Immediate post	Music–movement activities vs active classroom comparator	39/40	0.16	-0.28 to 0.61	Age 4–5 years; intervention mean 4.36 (SD 0.42), control 4.49 (SD 0.29) years; Classroom quasi-experimental intervention	Direct core-domain construct; immediate post; one effect per Study ID/domain/time; independent within domain; not selected by magnitude or significance.		10.1080/02568543.2022.2111482
ST107_WM	ST107 / 42	Hsiao 2025	Working memory	Chinese Working Memory Test for Young Children	Immediate post (360 min total)	Gesture-recognition physical learning vs traditional activity learning	35/32	0.68	0.19 to 1.18	Kindergarten level 3, ages 5–6 years; Quasi-experimental individual allocation within multi-class sample	Direct core-domain construct; immediate post; one effect per Study ID/domain/time; independent within domain; not selected by magnitude or significance.		10.1007/s11423-025-10471-4
ST005_CF	ST005 / 27	Zhang 2024	Cognitive flexibility	Dots Task accuracy	Immediate post (12 weeks)	Recreational gymnastics vs usual outdoor/no organized sport	31/32	0.69	0.18 to 1.20	Intervention 66.27 (SD 3.12) months; control 66.79 (SD 3.34) months; Individually randomized controlled trial	Direct core-domain construct; immediate post; one effect per Study ID/domain/time; independent within domain; not selected by magnitude or significance.	Hot-EF/delay-gratification tasks retained narratively; no single favourable task selected as a global effect.	10.1002/pchj.786
ST026_CF	ST026 / 38	Ilari 2021	Cognitive flexibility	Dimensional Change Card Sort score	Immediate post (5 weeks)	Musical activities vs control	51/52	0.42	0.03 to 0.81	Mean 4.7 years (SD 0.47), range 4–6 years; Individual random assignment within multi-class school sample	Direct core-domain construct; immediate post; one effect per Study ID/domain/time; independent within domain; not selected by magnitude or significance.		10.1177/20592043211054829
ST061_CF	ST061 / 30	Xiong 2017	Cognitive flexibility	Preschool Executive Function Measure total score	Immediate post (3 months)	Structured physical activity vs usual unstructured recess	19/20	1.07	0.39 to 1.74	Age 4–5 years; mean 4.67 years; Class-assigned controlled intervention / quasi-experimental	Direct core-domain construct; immediate post; one effect per Study ID/domain/time; independent within domain; not selected by magnitude or significance.		10.1155/2017/5635070
ST072_CF	ST072 / 13	Shen 2019	Cognitive flexibility	Dimensional Change Card Sort score	Immediate post (T2)	Music training vs free-play control	30/31	0.93	0.40 to 1.46	Music 51.39 (SD 4.27) months; control 50.35 (SD 3.38) months; Natural-class controlled intervention	Direct core-domain construct; immediate post; one effect per Study ID/domain/time; independent within domain; not selected by magnitude or significance.	Dot Matrix retained only as working-memory task-selection sensitivity.	10.3389/fpsyg.2019.01910
ST079_CF	ST079 / 40	Wu 2026	Cognitive flexibility	Dimensional Change Card Sort score	Immediate post	Aggregated music-style intervention vs control	98/17	0.52	0.01 to 1.04	Mean 59.02 months (SD 3.47), age 4–5 years; Individually randomized multi-arm trial with classroom context	Direct core-domain construct; immediate post; one effect per Study ID/domain/time; independent within domain; not selected by magnitude or significance.		10.3389/fpsyg.2026.1779286
ST081_CF	ST081 / 39	Shen 2020	Cognitive flexibility	Dimensional Change Card Sort score	Immediate post	Street-dance training vs free play/no training	30/30	0.91	0.38 to 1.45	Dance 52.40 (SD 3.95) months; control 55.68 (SD 4.23) months; Natural-class controlled intervention	Direct core-domain construct; immediate post; one effect per Study ID/domain/time; independent within domain; not selected by magnitude or significance.		10.3389/fpsyg.2020.585598
ST082_CF	ST082 / 32	Bai 2022	Cognitive flexibility	DCCS shifting behavioral-performance score	Immediate post	Group-play MVPA vs usual exercise	30/32	1.08	0.55 to 1.61	Age 4–5 years; intervention mean 4.16 (SD 0.29), control 4.70 (SD 0.43) years; Pilot cluster-randomized controlled trial	Direct core-domain construct; immediate post; one effect per Study ID/domain/time; independent within domain; not selected	Overall EF composite excluded as a deterministic duplicate of the three component domains.	10.3389/fpsyg.2022.847785

Effect ID	Study ID/ref.	Author/year	Domain	Selected outcome	Timepoint	Contrast	nI/nC	g	95% CI	Age/design note	Selection rule applied	Alternative/secondary handling	DOI
											by magnitude or significance.		
ST092_CF	ST092 / 33	Xiong 2019	Cognitive flexibility	DCCS executive-function score	Immediate post (8 weeks)	Exergaming vs traditional physical activity	30/30	0.50	-0.02 to 1.01	Age 4–5 years; Cluster-randomized pilot trial	Direct core-domain construct; immediate post; one effect per Study ID/domain/time; independent within domain; not selected by magnitude or significance.		10.3390/jcm8040469
ST100_CF	ST100 / 15	Suppalarkbunlue 2023	Cognitive flexibility	DCCS border score	Immediate post	Music–movement activities vs active classroom comparator	39/40	-0.50	-0.94 to -0.05	Age 4–5 years; intervention mean 4.36 (SD 0.42), control 4.49 (SD 0.29) years; Classroom quasi-experimental intervention	Direct core-domain construct; immediate post; one effect per Study ID/domain/time; independent within domain; not selected by magnitude or significance.		10.1080/02568543.2022.2111482

Supplementary Table S12. Age and cluster-adjustment sensitivity analyses

Table S12A reports developmental-age exclusions. Table S12B reports variance-inflation and extreme-exclusion scenarios for class- or cluster-assigned studies.

Table S12A. Age-at-exposure sensitivity analyses

Scenario	Domain	Excluded Study IDs	k	Hedges g	95% HKSJ CI	p	Interpretation
Primary frozen model	Inhibition		10	0.52	0.15 to 0.90	0.011	Reference model
Exclude younger-inclusive studies	Inhibition	ST076; ST096	8	0.63	0.18 to 1.08	0.013	Positive pooled estimate retained; no evidence that younger-inclusive studies drive the result.
Primary frozen model	Working memory		10	0.42	0.09 to 0.75	0.018	Reference model
Exclude younger-inclusive studies	Working memory	ST076	9	0.45	0.09 to 0.82	0.021	Positive pooled estimate retained; no evidence that younger-inclusive studies drive the result.
Primary frozen model	Cognitive flexibility		9	0.60	0.22 to 0.98	0.006	Reference model
Exclude younger-inclusive studies	Cognitive flexibility		9	0.60	0.22 to 0.98	0.006	Positive pooled estimate retained; no evidence that younger-inclusive studies drive the result.
Exclude upper-window borderline study	Inhibition	ST057	9	0.53	0.10 to 0.95	0.021	Effect remains positive and statistically compatible with the primary result.

Table S12B. Cluster-adjustment sensitivity analyses

Scenario	Domain	ICC assumption	Studies adjusted/excluded	k	Hedges g	95% HKSJ CI	95% PI	Interpretation
Primary frozen model	Inhibition			10	0.52	0.15 to 0.90	-0.57 to 1.62	Reference model
Primary frozen model	Working memory			10	0.42	0.09 to 0.75	-0.47 to 1.31	Reference model
Primary frozen model	Cognitive flexibility			9	0.60	0.22 to 0.98	-0.46 to 1.66	Reference model
Observed class ICC for ST026 only	Inhibition	ST026: .08 WM; .04 CF	ST026 where applicable	10	0.52	0.15 to 0.90	-0.57 to 1.62	Observed source-level class correlation does not materially change the pooled result.
Observed class ICC for ST026 only	Working memory	ST026: .08 WM; .04 CF	ST026 where applicable	10	0.44	0.11 to 0.76	-0.39 to 1.27	Observed source-level class correlation does not materially change the pooled result.
Observed class ICC for ST026 only	Cognitive flexibility	ST026: .08 WM; .04 CF	ST026 where applicable	9	0.61	0.22 to 0.99	-0.46 to 1.67	Observed source-level class correlation does not materially change the pooled result.
Variance-inflation scenario ICC=0.01	Inhibition	0.01 for eight unadjusted class/cluster-assigned studies; observed ICC for ST026	ST026; ST061; ST070; ST072; ST081; ST082; ST092; ST096; ST100 (as applicable)	10	0.51	0.14 to 0.89	-0.56 to 1.59	Pooled effect remains positive and statistically significant under the prespecified scenario.
Variance-inflation scenario ICC=0.01	Working memory	0.01 for eight unadjusted class/cluster-assigned studies; observed ICC for ST026	ST026; ST061; ST070; ST072; ST081; ST082; ST092; ST096; ST100 (as applicable)	10	0.43	0.11 to 0.75	-0.37 to 1.23	Pooled effect remains positive and statistically significant under the prespecified scenario.
Variance-inflation scenario ICC=0.01	Cognitive flexibility	0.01 for eight unadjusted class/cluster-assigned studies; observed ICC for ST026	ST026; ST061; ST070; ST072; ST081; ST082; ST092; ST096; ST100 (as applicable)	9	0.60	0.22 to 0.98	-0.43 to 1.63	Pooled effect remains positive and statistically significant under the prespecified scenario.
Variance-inflation scenario ICC=0.05	Inhibition	0.05 for eight unadjusted class/cluster-assigned studies; observed ICC for ST026	ST026; ST061; ST070; ST072; ST081; ST082; ST092; ST096; ST100 (as applicable)	10	0.48	0.11 to 0.85	-0.52 to 1.48	Pooled effect remains positive and statistically significant under the prespecified scenario.
Variance-inflation scenario ICC=0.05	Working memory	0.05 for eight unadjusted class/cluster-assigned studies; observed ICC for ST026	ST026; ST061; ST070; ST072; ST081; ST082; ST092; ST096; ST100 (as applicable)	10	0.41	0.09 to 0.73	-0.31 to 1.14	Pooled effect remains positive and statistically significant under the prespecified scenario.
Variance-inflation scenario ICC=0.05	Cognitive flexibility	0.05 for eight unadjusted class/cluster-assigned studies; observed ICC for ST026	ST026; ST061; ST070; ST072; ST081; ST082; ST092; ST096; ST100 (as applicable)	9	0.57	0.20 to 0.94	-0.28 to 1.42	Pooled effect remains positive and statistically significant under the prespecified scenario.
Variance-inflation scenario ICC=0.10	Inhibition	0.10 for eight unadjusted class/cluster-assigned studies; observed ICC for ST026	ST026; ST061; ST070; ST072; ST081; ST082; ST092; ST096; ST100 (as applicable)	10	0.45	0.08 to 0.81	-0.50 to 1.39	Pooled effect remains positive and statistically significant under the prespecified scenario.
Variance-inflation scenario ICC=0.10	Working memory	0.10 for eight unadjusted class/cluster-assigned studies; observed ICC for ST026	ST026; ST061; ST070; ST072; ST081; ST082; ST092; ST096; ST100 (as applicable)	10	0.41	0.09 to 0.72	-0.28 to 1.09	Pooled effect remains positive and statistically significant under the prespecified scenario.
Variance-inflation scenario ICC=0.10	Cognitive flexibility	0.10 for eight unadjusted class/cluster-assigned studies; observed ICC for ST026	ST026; ST061; ST070; ST072; ST081; ST082; ST092; ST096; ST100 (as applicable)	9	0.53	0.21 to 0.86	0.07 to 1.00	Pooled effect remains positive and statistically significant under the prespecified scenario.
Extreme exclusion of eight unadjusted class/cluster-assigned studies	Inhibition	Not applicable	Excluded: ST072; ST081; ST082; ST096; ST100	5	0.38	-0.26 to 1.02	-1.00 to 1.77	Positive point estimate retained; inhibition/working-memory precision becomes insufficient after halving k.

Scenario	Domain	ICC assumption	Studies adjusted/excluded	k	Hedges g	95% HKSJ CI	95% PI	Interpretation
Extreme exclusion of eight unadjusted class/cluster-assigned studies	Working memory	Not applicable	Excluded: ST070; ST072; ST081; ST082; ST100	5	0.39	-0.13 to 0.92	-0.73 to 1.51	Positive point estimate retained; inhibition/working-memory precision becomes insufficient after halving k.
Extreme exclusion of eight unadjusted class/cluster-assigned studies	Cognitive flexibility	Not applicable	Excluded: ST061; ST072; ST081; ST082; ST092; ST100	3	0.52	0.18 to 0.87	0.18 to 0.87	Positive effect retained, but only three studies remain.

Supplementary File S1. Search Documentation and Reproducibility Strategies

Early Childhood Movement- and Arts-Based Experiences and Executive Functions: A Systematic Review and Meta-analysis

PROSPERO CRD420261363924 | Revised peer-review package, 9 August 2026

Provenance statement. The historical PROSPERO appendix preserved complete draft strings for PubMed/MEDLINE, Web of Science and Scopus and supplementary Google Scholar queries. The completed review used seven formal databases. Complete date-stamped platform exports, exact source-specific execution dates and per-database yields were not preserved for every source. Accordingly, the seven final database strings below are labelled database-adapted reproducibility reconstructions. They reproduce the registered conceptual blocks and final fields/controlled vocabulary but are not represented as verbatim historical execution logs. Searches covered database inception through April 2026 without date or language restrictions.

S1.1. Registered historical strategy

The following strings reproduce the search strategy uploaded with the original PROSPERO record. The historical appendix is also publicly available through PROSPERO.

PubMed/MEDLINE (registered draft)

```
(
("Motor Activity"[MeSH] OR "Exercise"[MeSH] OR "Play and Playthings"[MeSH] OR "Sports"[MeSH]
OR "physical activity"[tiab] OR exercise[tiab] OR sport*[tiab] OR "active play"[tiab] OR movement[tiab]
OR "motor practice"[tiab] OR "motor development"[tiab])
OR
("Music"[MeSH] OR "Art"[MeSH] OR "Dancing"[MeSH] OR "Theater"[MeSH]
OR "music training"[tiab] OR "music lesson*"[tiab] OR "music education"[tiab]
OR "arts participation"[tiab] OR "arts education"[tiab] OR "visual arts"[tiab] OR "performing arts"[tiab]
OR theatre[tiab] OR theater[tiab] OR drama[tiab] OR dance[tiab] OR "creative arts"[tiab])
)
AND
(
"Executive Function"[MeSH] OR "Inhibition, Psychological"[MeSH] OR "Memory, Short-Term"[MeSH]
OR "Cognition"[MeSH] OR "Spatial Navigation"[MeSH]
OR "executive function*"[tiab] OR "executive control"[tiab] OR inhibit*[tiab]
OR "working memory"[tiab] OR "cognitive flexib*"[tiab] OR "cognitive control"[tiab]
OR visuospatial[tiab] OR "visuo-spatial"[tiab] OR "spatial skill*"[tiab] OR "spatial abilit*"[tiab]
OR "spatial reasoning"[tiab] OR "mental rotation"[tiab] OR "spatial memory"[tiab] OR "spatial cognition"[tiab]
OR planning[tiab]
)
AND
(
"Child, Preschool"[MeSH] OR "Infant"[MeSH] OR "Child Development"[MeSH]
OR preschool*[tiab] OR "pre-school*"[tiab] OR "early childhood"[tiab] OR toddler*[tiab]
OR kindergarten*[tiab] OR infant*[tiab] OR "young children"[tiab] OR "early years"[tiab]
OR "0-6 year*"[tiab] OR "under 6"[tiab] OR "under six"[tiab] OR "nursery school"[tiab]
OR daycare[tiab] OR "day care"[tiab]
)
NOT (animals[mh] NOT humans[mh])
```

Web of Science Core Collection (registered draft)

```
TS=((
"physical activity" OR exercise OR "active play" OR "motor practice" OR "motor development"
OR sport* OR movement OR "physical education"
OR "music training" OR "music lesson*" OR "music education"
OR "arts participation" OR "arts education" OR "visual arts" OR "performing arts"
OR theatre OR theater OR drama OR dance OR "creative arts"
)
AND (
"executive function*" OR "executive control" OR inhibit* OR "working memory"
OR "cognitive flexib*" OR "cognitive control"
OR visuospatial OR "visuo-spatial" OR "spatial skill*" OR "spatial abilit*"
OR "spatial reasoning" OR "mental rotation" OR "spatial memory" OR "spatial cognition" OR planning
)
AND (
preschool* OR "pre-school*" OR "early childhood" OR toddler* OR kindergarten*
OR infant* OR "young children" OR "early years" OR "0-6 year*" OR "under 6"
OR "nursery school" OR daycare OR "day care"
))
```

Scopus (registered draft)

```
TITLE-ABS-KEY((
"physical activity" OR exercise OR "active play" OR "motor practice" OR "motor development"
OR sport* OR movement OR "physical education"
OR "music training" OR "music lesson*" OR "music education"
OR "arts participation" OR "arts education" OR "visual arts" OR "performing arts"
OR theatre OR theater OR drama OR dance OR "creative arts"
)
AND (
"executive function*" OR "executive control" OR inhibit* OR "working memory"
OR "cognitive flexib*" OR "cognitive control"
OR visuospatial OR "visuo-spatial" OR "spatial skill*" OR "spatial abilit*"
OR "spatial reasoning" OR "mental rotation" OR "spatial memory" OR "spatial cognition" OR planning
)
AND (
preschool* OR "pre-school*" OR "early childhood" OR toddler* OR kindergarten*
OR infant* OR "young children" OR "early years" OR "0-6 year*" OR "under 6"
OR "nursery school" OR daycare OR "day care"
))
```

Google Scholar supplementary query (registered draft; not retained as a formal final source)

"early childhood" preschool (visuospatial OR "mental rotation" OR "spatial reasoning" OR "executive function*" OR "working memory") ("physical activity" OR sport OR exercise OR "active play" OR "music training" OR "music lessons" OR "arts education" OR dance OR theatre OR drama)

S1.2. Final seven-database reproducibility reconstructions

No date or language restrictions were used. Exact source-specific execution dates and yields were not preserved and are therefore not imputed.

PubMed/MEDLINE

```
(
("Motor Activity"[MeSH] OR "Exercise"[MeSH] OR "Play and Playthings"[MeSH] OR "Sports"[MeSH]
OR "physical activity"[tiab] OR exercise[tiab] OR sport*[tiab] OR "active play"[tiab] OR movement[tiab]
OR "motor practice"[tiab] OR "motor development"[tiab])
OR
("Music"[MeSH] OR "Art"[MeSH] OR "Dancing"[MeSH] OR "Theater"[MeSH]
```

OR "music training"[tiab] OR "music lesson*"[tiab] OR "music education"[tiab]
OR "arts participation"[tiab] OR "arts education"[tiab] OR "visual arts"[tiab] OR "performing arts"[tiab]
OR theatre[tiab] OR theater[tiab] OR drama[tiab] OR dance[tiab] OR "creative arts"[tiab])
)
AND
(
"Executive Function"[MeSH] OR "Inhibition, Psychological"[MeSH] OR "Memory, Short-Term"[MeSH]
OR "Cognition"[MeSH] OR "Spatial Navigation"[MeSH]
OR "executive function*"[tiab] OR "executive control"[tiab] OR inhibit*[tiab]
OR "working memory"[tiab] OR "cognitive flexib*"[tiab] OR "cognitive control"[tiab]
OR visuospatial[tiab] OR "visuo-spatial"[tiab] OR "spatial skill*"[tiab] OR "spatial abilit*"[tiab]
OR "spatial reasoning"[tiab] OR "mental rotation"[tiab] OR "spatial memory"[tiab] OR "spatial cognition"[tiab]
OR planning[tiab]
)
AND
(
"Child, Preschool"[MeSH] OR "Infant"[MeSH] OR "Child Development"[MeSH]
OR preschool*[tiab] OR "pre-school*"[tiab] OR "early childhood"[tiab] OR toddler*[tiab]
OR kindergarten*[tiab] OR infant*[tiab] OR "young children"[tiab] OR "early years"[tiab]
OR "0-6 year*"[tiab] OR "under 6"[tiab] OR "under six"[tiab] OR "nursery school"[tiab]
OR daycare[tiab] OR "day care"[tiab]
)
NOT (animals[mh] NOT humans[mh])

Web of Science Core Collection (SCIE/SSCI)

TS=((
"physical activity" OR exercise OR "active play" OR "motor practice" OR "motor development"
OR sport* OR movement OR "physical education"
OR "music training" OR "music lesson*" OR "music education"
OR "arts participation" OR "arts education" OR "visual arts" OR "performing arts"
OR theatre OR theater OR drama OR dance OR "creative arts"
)
AND (
"executive function*" OR "executive control" OR inhibit* OR "working memory"
OR "cognitive flexib*" OR "cognitive control"
OR visuospatial OR "visuo-spatial" OR "spatial skill*" OR "spatial abilit*"
OR "spatial reasoning" OR "mental rotation" OR "spatial memory" OR "spatial cognition" OR planning
)
AND (
preschool* OR "pre-school*" OR "early childhood" OR toddler* OR kindergarten*
OR infant* OR "young children" OR "early years" OR "0-6 year*" OR "under 6"
OR "nursery school" OR daycare OR "day care"
))

Scopus

TITLE-ABS-KEY((
"physical activity" OR exercise OR "active play" OR "motor practice" OR "motor development"
OR sport* OR movement OR "physical education"
OR "music training" OR "music lesson*" OR "music education"
OR "arts participation" OR "arts education" OR "visual arts" OR "performing arts"
OR theatre OR theater OR drama OR dance OR "creative arts"
)
AND (
"executive function*" OR "executive control" OR inhibit* OR "working memory"
OR "cognitive flexib*" OR "cognitive control"
OR visuospatial OR "visuo-spatial" OR "spatial skill*" OR "spatial abilit*"
OR "spatial reasoning" OR "mental rotation" OR "spatial memory" OR "spatial cognition" OR planning
)

AND (
preschool* OR "pre-school*" OR "early childhood" OR toddler* OR kindergarten*
OR infant* OR "young children" OR "early years" OR "0-6 year*" OR "under 6"
OR "nursery school" OR daycare OR "day care"
))

ERIC (EBSCOhost reconstruction)

TX (preschool* OR pre-school* OR "early childhood" OR toddler* OR kindergarten* OR infant* OR "young child*" OR "early years" OR "0-6 year*" OR "under 6" OR "under six" OR "nursery school" OR daycare OR "day care") AND TX (("physical activity" OR exercise OR "active play" OR "motor practice" OR "motor development" OR sport* OR movement OR "physical education") OR ("music training" OR "music lesson*" OR "music education" OR "arts participation" OR "arts education" OR "visual arts" OR "performing arts" OR theatre OR theater OR drama OR dance OR "creative arts")) AND TX ("executive function*" OR "executive control" OR inhibit* OR "working memory" OR "cognitive flexib*" OR "cognitive control" OR visuospatial OR "visuo-spatial" OR "spatial skill*" OR "spatial abilit*" OR "spatial reasoning" OR "mental rotation" OR "spatial memory" OR "spatial cognition" OR planning)

CINAHL (EBSCOhost reconstruction)

(MH "Child, Preschool+" OR MH "Infant+" OR TX (preschool* OR pre-school* OR "early childhood" OR toddler* OR kindergarten* OR infant* OR "young child*" OR "early years" OR "0-6 year*" OR "under 6" OR "under six" OR "nursery school" OR daycare OR "day care")) AND ((MH "Motor Activity+" OR MH "Exercise+" OR MH "Play and Playthings+" OR TX (("physical activity" OR exercise OR "active play" OR "motor practice" OR "motor development" OR sport* OR movement OR "physical education") OR ("music training" OR "music lesson*" OR "music education" OR "arts participation" OR "arts education" OR "visual arts" OR "performing arts" OR theatre OR theater OR drama OR dance OR "creative arts"))) AND (MH "Executive Function+" OR MH "Memory, Short-Term+" OR TX ("executive function*" OR "executive control" OR inhibit* OR "working memory" OR "cognitive flexib*" OR "cognitive control" OR visuospatial OR "visuo-spatial" OR "spatial skill*" OR "spatial abilit*" OR "spatial reasoning" OR "mental rotation" OR "spatial memory" OR "spatial cognition" OR planning)))

SPORTDiscus (EBSCOhost reconstruction)

TX (preschool* OR pre-school* OR "early childhood" OR toddler* OR kindergarten* OR infant* OR "young child*" OR "early years" OR "0-6 year*" OR "under 6" OR "under six" OR "nursery school" OR daycare OR "day care") AND TX (("physical activity" OR exercise OR "active play" OR "motor practice" OR "motor development" OR sport* OR movement OR "physical education") OR ("music training" OR "music lesson*" OR "music education" OR "arts participation" OR "arts education" OR "visual arts" OR "performing arts" OR theatre OR theater OR drama OR dance OR "creative arts")) AND TX ("executive function*" OR "executive control" OR inhibit* OR "working memory" OR "cognitive flexib*" OR "cognitive control" OR visuospatial OR "visuo-spatial" OR "spatial skill*" OR "spatial abilit*" OR "spatial reasoning" OR "mental rotation" OR "spatial memory" OR "spatial cognition" OR planning)

PSICODOC (title/abstract/keywords reconstruction)

(preschool* OR pre-school* OR "early childhood" OR toddler* OR kindergarten* OR infant* OR "young child*" OR "early years" OR "0-6 year*" OR "under 6" OR "under six" OR "nursery school" OR daycare OR "day care") AND (("physical activity" OR exercise OR "active play" OR "motor practice" OR "motor development" OR sport* OR movement OR "physical education") OR ("music training" OR "music lesson*" OR "music education" OR "arts participation" OR "arts education" OR "visual arts" OR "performing arts" OR theatre OR theater OR drama OR dance OR "creative arts")) AND ("executive function*" OR "executive control" OR inhibit* OR "working memory" OR "cognitive flexib*" OR "cognitive control" OR visuospatial OR "visuo-spatial" OR "spatial skill*" OR "spatial abilit*" OR "spatial reasoning" OR "mental rotation" OR "spatial memory" OR "spatial cognition" OR planning)

S1.3. Search-record limitations

- The combined database identification count was 6,126 records. Per-database yields were not preserved for every source and were not retrospectively apportioned.
- The archive retained 13 reports in a combined retrieval-stage category (not retrieved or not assessable). A report-level split was unavailable and was not reconstructed.
- Reference lists and relevant reviews were used for contextual checking. They did not contribute additional eligible records to the final PRISMA identification flow.
- Google Scholar appeared in the original registered draft but was not retained as a formal source in the completed PRISMA flow.

Supplementary Table S13. Pedagogical format, cognitive-engagement and implementation-characteristics coding of the 15 core interventions

Coding definitions. Structured interventions were adult-designed programmes with predefined goals, scheduled sessions, explicit instructions and a planned sequence or progression. Guided play retained meaningful child agency within an adult-defined goal, environment or task constraints, with scaffolding when required. Free/unstructured play was predominantly child-initiated and child-directed without a standardised instructional sequence or planned progression. Cognitively engaging/enriched indicated deliberate executive demands beyond physical or artistic execution alone, including rule maintenance/updating, inhibition, switching, sequencing, changing-cue response, problem solving, dual-task coordination or decision making. NR = not reported. Coding was descriptive and was not used for a post hoc moderator meta-analysis.

Summary: all 15 core interventions were adult-designed; 12 were primarily structured and 3 were guided-play/structured hybrids. No free/unstructured-play intervention contributed to the quantitative core. Reporting of progression, child agency, scaffolding and fidelity was uneven.

Study ID/ref.	Content family	Primary pedagogical format	Guidance / child agency	Cognitive-engagement features	Progression / complexity	Fidelity reporting	Interpretive coding note
ST005 [27]	Movement/motor: recreational gymnastics	Structured adult-led	Instructor-led; limited child choice reported	Rule following, sequencing and response control embedded; not formally labelled cognitively enriched	Planned 12-week programme; progression partly reported	Dose reported; fidelity NR	Structured motor programme; cognitive enrichment not assumed from the label alone.
ST026 [38]	Arts/mixed: music, instruments and movement	Structured play-based arts	Teacher-led fixed sequence with child improvisation and some choice	Explicit inhibition, working-memory and flexibility demands through songs, gestures, instruments and switching	Seven-component lesson sequence; teacher repertoire choice within components	Teacher training and curriculum reported; adherence partial	Structured rather than free play; contains guided-play elements.
ST057 [28]	Movement/motor: coordination training	Guided-play/structured hybrid	Adult-designed game format with open-ended child responses	Spatial-temporal control, motor planning and response inhibition	Open-ended tasks explicitly progressed from simple to complex	Frequency/duration reported; fidelity NR	Guided-play elements delivered within a structured programme.
ST061 [30]	Movement/motor: structured physical activity	Structured adult-led	Intact-class adult-directed programme	Executive demands not sufficiently operationalised in the report	Three-month programme; progression NR	Fidelity NR	Do not label cognitively enriched without source detail.
ST070 [36]	Movement/motor: programmed physical activity	Structured adult-led	Classroom-led programmed sessions	Working-memory relevance plausible; explicit cognitive-load features insufficiently reported	Eight-week programmed intervention; progression partial	Dose reported; fidelity partial	More structured than the comparator's unstructured gross-motor activity.
ST072 [13]	Arts: music training	Structured adult-led arts	Teacher-directed lessons; limited agency reported	Sequencing, memory, inhibition and flexible rule use embedded	Twelve-week curriculum; progression partly described	Attendance/dose reported; fidelity partial	Free play was the comparator, not the intervention.
ST076 [31]	Movement/motor: mini-trampoline	Structured adult-led	Instructor-supervised; limited child choice	Motor planning and balance demands; cognitive enrichment not explicitly operationalised	Twelve-week planned protocol; progression partial	Dose/adherence reported; fidelity partial	Structured exercise, not guided or free play.
ST079 [40]	Arts: rhythm and child-appropriate music styles	Structured adult-led arts	Daily instructor-led clapping synchronised to assigned music	Timing, cue response, rule maintenance and updating explicitly engaged	Standardised 10-min weekday schedule; complexity varied by musical style	Exposure standardised; fidelity partial	Cognitively engaging through rhythmic synchronisation and cue response.
ST081 [39]	Arts/movement: street-dance training	Structured adult-led arts-movement	Instructor-led choreography; limited-to-moderate agency	Programme explicitly integrated inhibition, working memory and flexibility through sequencing and switching	Twenty-four sessions over eight weeks; planned progression	Dose reported; fidelity partial	Highly structured dance rather than free dance.
ST082 [32]	Movement/motor: group-play MVPA	Guided play	Adult-set group tasks with peer interaction and child agency	Rules, inhibition, switching and coordination embedded	Planned intervention; progression partial	Dose reported; fidelity partial	Guided play, not unstructured free play.
ST092 [33]	Movement/motor: exergaming	Structured digital play	Game/system rules with adult supervision	Rapid cue response, decision making, inhibition and switching	Standardised game levels/progression	Dose reported; fidelity partial	Playful delivery within a structured digital task.
ST096 [35]	Movement/motor + learning: Move for Thought	Guided-play/structured hybrid	Educator-scaffolded playful activities with child participation	Explicit integration of motor, academic, executive and social-emotional demands	Eight-week curriculum with planned activity progression	Feasibility/usability and dose reported; fidelity partial	Explicit cognitively enriched programme.

Study ID/ref.	Content family	Primary pedagogical format	Guidance / child agency	Cognitive-engagement features	Progression / complexity	Fidelity reporting	Interpretive coding note
ST100 [15]	Mixed arts-movement: music-movement	Structured adult-led mixed programme	Classroom-led participatory activities	Rhythm, no-go response, sequence memory and switching explicitly engaged	Planned classroom sequence; progression partial	Dose reported; fidelity partial	Structured mixed intervention rather than free play.
ST107 [42]	Movement-cognitive: gesture recognition plus memory strategy	Structured technology-supported	Adult/system-guided tasks; constrained choice	Explicit memory strategy, gesture-response mapping and updating	Standardised 360-min sequence	Implementation protocol reported; fidelity partial	Explicit cognitively enriched learning activity.
ST114 [44]	Arts: music training	Structured adult-led arts	Instructor-led; active sports comparator	Timing, rule following and motor inhibition embedded; enrichment not formally labelled	Planned training; progression partial	Dose reported; fidelity NR/partial	Structured music training, not free play.
